# Supplementary material for: Design, Synthesis and Bioactivity of Novel Pyrimidine Sulfonate Esters Containing Thioether Moiety
Source: Int J Mol Sci. 2023 Feb 28;24(5):4691. doi: 10.3390/ijms24054691 (PMC10003536; doi:10.3390/ijms24054691)
Supplement: Supplementary file 1 [file ijms-24-04691-s001.zip › ijms-2216026-supplementary.pdf]

Design, synthesis, and bioactivity of novel pyrimidine sulfonate esters containing thioether moiety

Changkun Li, Youhua Liu, Xiaoli Ren, Yanni Tan, Linhong Jin * and Xia Zhou
State Key Laboratory Breeding Base of Green Pesticide and Agricultural Bioengineering, Key Laboratory of Green Pesticide and Agricultural Bioengineering, Ministry of Education, Guizhou University, Guiyang 550025, China
* Correspondence: lhjin@gzu.edu.cn; Tel.: +86-851-3620-521

Table S1. Crystallographic data of compound A_{10}.

**parameters**	**Data**
chemical formula	$C_{12}H_{10}ClFN_2O_3S_2$
formula weight	348.79
crystal system	Monoclinic
Space group	P2(1)/c
a (Å)	11.2173(3)
b (Å)	11.3085(3)
c (Å)	12.7427(3)
α (deg)	90
β (deg)	112.5070(10)
γ (deg)	90
V (Å)	1493.30(7)
Z	4
ρ_{calc}g/cm^3	1.551
μ/mm^{-1}	5.095
F (0 0 0)	712.0
Crystal size/mm^3	0.14 x 0.12 x 0.1
Radiation	CuKα (λ = 1.54178)
2Θ range for data collection°	10.848 to 144.58
Index ranges	-13 < h < 13, -13 < k < 13, -13 <I < 15
Reflections collected	13655
Independent reflections	2922 [R_{int} = 0.0546, R_{sigma} = 0.0410)
Data/restraints/parameters	2922/0/192
Goodness-of-fit on F^2	1.062

Table S2 In Vitro inhibition rate(%)[a] of title compound A_1-A_{33} against *Xoo, Xac, Psa* and Rs.

**Compd.**	***Xoo***		***Xac***		***Psa***		**Rs**	
	**100 μg/mL**	**50 μg/mL**	**100 μg/mL**	**50 μg/mL**	**100 μg/mL**	**50 μg/mL**	**100 μg/mL**	**50 μg/mL**
**A_1**	86±2.39	65±1.04	51±4.27	47±3.48	35±1.73	16±3.75	16±1.22	7±1.78
**A_2**	73±3.55	69±2.80	42±1.42	21±1.25	14±3.50	9±4.15	13±1.17	9±2.82
**A_3**	83±1.60	68±1.29	51±2.39	47±1.56	9±2.01	5±1.04	0±1.41	0±1.25
**A_4**	59±0.95	50±2.49	33±2.29	23±3.42	8±0.15	0±2.62	13±3.14	10±1.06
**A_5**	100±0.40	100±0.23	51±1.56	46±1.90	29±0.58	22±1.75	16±3.86	9±0.37
**A_6**	61±3.00	49±1.51	48±3.32	41±1.99	22±1.95	13±2.33	1±2.36	0±3.26
**A_7**	83±1.20	78±1.42	35±1.49	19±1.12	9±0.91	6±2.46	9±2.70	6±2.16
**A_8**	62±4.61	36±0.83	39±4.57	25±4.45	24±3.59	9±2.64	16±2.49	14±2.23
**A_9**	36±1.04	24±1.20	14±1.68	5±1.49	19±2.79	11±2.46	20±2.52	12±4.89
**A_{10}**	69±1.89	55±2.34	13±1.27	0±1.81	12±3.53	6±1.44	16±4.44	9±3.43
**A_{11}**	48±1.25	36±3.88	22±3.63	7±1.46	16±0.93	9±1.18	18±1.03	0±0.47
**A_{12}**	62±1.25	38±1.33	29±2.17	12±2.21	8±0.45	4±2.74	0±1.69	0±1.78
**A_{13}**	65±0.47	44±2.53	5±1.61	0±3.06	12±2.54	4±1.72	15±3.59	8±3.53
**A_{14}**	68±0.45	46±0.52	36±3.08	24±1.03	15±0.45	9±1.55	9±1.24	0±1.07
**A_{15}**	58±1.71	43±1.72	26±2.76	4±1.15	22±2.02	13±1.76	12±3.18	5±2.99

**A_{16}**	65±2.75	50±0.82	20±3.15	8±4.34	21±1.72	8±2.48	34±0.47	16±2.91
**A_{17}**	57±1.33	51±0.13	40±2.07	10±4.42	0±1.39	0±0.76	10±0.47	4±0.42
**A_{18}**	53±1.02	33±1.93	20±1.64	7±2.52	12±2.17	8±1.42	6±0.87	0±1.90
**A_{19}**	25±1.64	19±1.25	43±3.71	25±2.43	9±1.01	0±1.98	8±3.34	0±2.16
**A_{20}**	75±3.18	63±0.91	49±1.60	35±1.82	12±2.17	8±1.42	12±3.10	6±4.34
**A_{21}**	73±1.37	69±2.62	28±1.03	15±0.70	7±3.37	0±4.06	0±1.50	0±0.27
**A_{22}**	61±1.40	57±2.72	42±1.77	19±1.61	8±4.36	4±3.66	23±1.73	17±0.71
**A_{23}**	69±2.02	58±1.07	21±0.82	16±1.31	17±1.33	5±1.83	15±1.83	10±1.13
**A_{24}**	45±2.39	42±0.99	30±2.56	23±1.48	30±3.44	13±3.86	27±1.46	16±1.44
**A_{25}**	66±0.26	61±3.10	23±1.81	10±1.48	11±3.36	4±2.65	15±4.88	5±2.63
**A_{26}**	58±3.78	34±1.12	34±1.39	12±3.40	1±3.14	0±2.07	10±2.16	0±1.24
**A_{27}**	79±3.41	69±1.84	35±1.68	10±1.58	13±1.68	9±3.50	22±1.90	11±0.91
**A_{28}**	52±1.20	48±0.45	26±4.22	0±1.42	22±0.98	11±0.93	16±3.82	7±4.95
**A_{29}**	28±1.74	25±1.44	0±1.94	0±4.34	5±1.34	0±4.34	0±0.98	0±2.18
**A_{30}**	89±1.83	62±2.28	41±3.03	21±0.96	29±1.22	10±1.01	15±1.51	5±0.99
**A_{31}**	100±0.76	100±0.51	44±1.58	33±2.20	28±1.89	15±1.33	18±1.91	8±1.12
**A_{32}**	51±1.83	37±2.86	51±3.67	24±4.90	25±2.11	12±1.18	19±1.55	9±1.25
**A_{33}**	100±1.78	88±0.76	64±2.29	52±2.85	31±2.04	14±1.37	25±2.22	12±1.99
**BT[b]**	100±1.14	77±1.46	57±1.18	41±0.64	-	-	-	-
**TZ[b]**	100±4.89	100±2.36	100±1.48	87±0.90	97±2.64	40±1.70	60±3.24	42±1.13
**TC[b]**	100±1.86	54±1.12	40±0.57	27±2.32	37±2.80	22±1.70	27±2.05	11±1.76

Notes: [a]Average of three replicates. [b]Commercialized bactericides bismerthiazol (BT), Thiazolyl zinc (TZ), and thiodiazole copper (TC). – indicates not tested.

Table S3 EC_{50} (μg/mL)[a] value of some title compounds against *Xoo* and *Xac*.

Compd.	*Xoo*			*Xac*		
	Regression eq.	r	EC_{50}(μg/mL)	Regression eq.	r	EC_{50}(μg/mL)
**A_1**	y=1.2263x+3.4922	0.96	16.94±1.88	y=1.0699x+2.9696	0.94	79.02±2.43
**A_3**	y=1.2644x+3.3805	0.99	19.09±1.77	y=1.1546x+2.7886	0.96	82.28±1.48
**A_5**	y=1.872x+3.8251	0.99	4.24±1.08	y=0.6668x+3.7354	0.98	78.80±2.19
**A_6**	-	-	-	y=0.5587x+3.8282	0.97	125.13±2.57
**A_7**	y=1.26x+3.542	0.97	14.35±1.22	-	-	-
**A_{30}**	y=2.338x+1.4487	0.99	33.03±1.98	y=1.3985x+1.9319	0.98	156.26±2.11
**A_{31}**	y=2.9687x+2.5339	0.98	6.77±1.22	y=1.0497x+2.7707	0.99	132.95±2.05
**A_{32}**	y=2.1259x+0.9405	0.92	81.19±3.01	y=1.4739x+1.9468	0.95	117.90±1.56
**A_{33}**	y=1.7035x+3.3461	0.98	9.35±2.04	y=1.2391x+2.9623	0.94	44.11±1.98
**BT[b]**	y=2.2097x +1.9071	0.99	25.12±1.03	y=1.3896x +2.3833	0.98	76.38±3.23
**TC[b]**	y=1.4075x+2.6628	0.99	45.81±1.72	y=1.5038x+1.8186	0.94	130.49±2.50

Notes: [a]Average of three replicates. [b]Commercialized bactericides bismerthiazol (BT) and thiodiazole copper (TC). – indicates not tested.

Table S4 Protective and curative Activities of compound A_5 against Rice Bacterial Leaf Blight under greenhouse conditions at 200 μg/mL *in Vivo*[a].

**Treatment**	**Protective activity (14 days after spraying)**			**Curative activity (14 days after spraying)**		
	**Morbidity (%)**	**Disease index (%)[b]**	**Control efficiency (%)[c]**	**Morbidity (%)**	**Disease index (%)[b]**	**Control efficiency (%)[c]**
**A_5**	100	16.23	47.85±1.62b	100	17.33	41.05±2.21b

**TC**	100	16.92	45.66±0.85c	100	20.05	31.81±1.34c
**BT**	100	13.38	57.02±1.79a	100	13.95	52.54±3.53a
**CK**	100	31.27	-	100	29.40	-

Notes: [a]All results are expressed as mean ± SD. [b]Disease index, which is a comprehensive indicator of the overall incidence and severity. [c]Statistical analysis was performed by analysis of variance (ANOVA) in SPSS 21.0 software with equal variances assumed ($P > 0.05$). The different lowercase letters indicate curative activity with different treatment groups at $P < 0.05$.

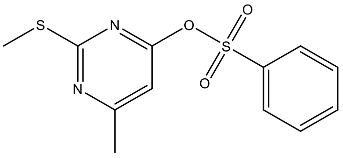

6-methyl-2-(methylthio)pyrimidin-4-yl-benzenesulfonate(A_1): Yellow solid，m.p.49-51℃；Yield：75%, ^{1}H NMR (400 MHz, $CDCl_3$) δ 8.12 – 7.99 (m, 2H, Ph-H), 7.74 – 7.65 (m, 1H, Ph-H), 7.65 – 7.52 (m, 2H, Ph-H), 6.58 (s, 1H, Pyrimidine-H), 2.44 (s, 3H, -S CH$_3$), 2.30 (s, 3H, Pyrimidine-CH$_3$). ^{13}C NMR (100 MHz, $CDCl_3$) δ 172.90, 1 71.29, 163.83, 136.88, 134.56, 129.24, 128.72, 105.29, 77.48, 77.36, 77.16, 76. 84, 24.26, 14.08. HRMS (ESI) calcd for $C_{12}H_{13}O_3N_2S_2$ [M＋H]$^+$: 297.0368, fo und 297.0364.

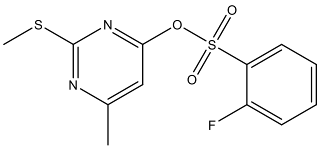

6-methyl-2-(methylthio)pyrimidin-4-yl-2-fluorobenzenesulfonate(A_2): White solid，m.p.44-46℃；Yield：75%, ^{1}H NMR (400 MHz, $CDCl_3$) δ 8.03 – 7.94 (m, 1H, Ph-H), 7.76 – 7.65 (m, 1H, Ph-H), 7.39 – 7.23 (m, 2H, Ph-H), 6.61 (s, 1H, Pyrimidine-H), 2.46 (s, 3H, -SCH$_3$), 2.19 (s, 3H, Pyrimidine-CH$_3$). ^{13}C NMR (100 MHz, $CDCl_3$) δ 172.97, 171.55, 163.83, 160.66, 158.07, 136.92 (d, J = 8.6 Hz), 131.18, 125.58 (d, J = 13.6 Hz), 124.55(d, J = 3.9 Hz), 117.60(d, J = 21.0 Hz), 105.26, 24.2 8, 13.95. ^{19}F NMR (376 MHz, $CDCl_3$) δ -107.06. HRMS (ESI) calcd for $C_{12}H_{12}O_3N_2FS_2$ [M＋H]$^+$: 315.0273, found 3315.0270.

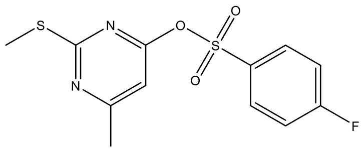

6-methyl-2-(methylthio)pyrimidin-4-yl-4-fluorobenzenesulfonate(A_3): White solid，m.p.69-71℃；Yield：74%, ^{1}H NMR (400 MHz, $CDCl_3$) δ 8.12 – 8.04 (m, 2H, Ph-H), 7.31 – 7.21 (m, 2H, Ph-H), 6.58 (s, 1H, Pyrimidine-H), 2.46 (s, 3H, -SCH$_3$), 2.37 (s, 3H, Pyrimidine-CH$_3$). ^{13}C NMR (100 MHz, $CDCl_3$) δ 172.88, 171.41, 168.20 (d, J = 256.0 Hz), 163.78, 132.81 (d, J = 3.3 Hz), 131.90 (d, J = 9.8 Hz), 116.63 (d, J = 23.0 Hz), 105.26, 24.31, 14.12. ^{19}F NMR (376 MHz, $CDCl_3$) δ -101.5 1. HRMS (ESI) calcd for $C_{12}H_{12}O_3N_2FS_2$ [M＋H]$^+$: 315.0268, found 315.0270.

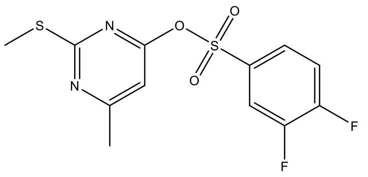

6-methyl-2-(methylthio)pyrimidin-4-yl-3,4-difluorobenzenesulfonate(A_4): White solid，m.p.46-48℃; Yield：72%, ^{1}H NMR (500 MHz, DMSO) δ 8.28 (ddd, J = 9.6, 7.2, 2.3 Hz, 1H, Ph-H), 8.02 – 7.93 (m, 2H, Ph-H), 7.86 – 7.76 (m, 1H, Ph-H), 6.99 (s, 1H, Pyrimidine-H), 2.43 (s, 3H, -SCH$_3$), 2.30 (s, 3H, Pyrimidine-CH$_3$). ^{13}C NMR (125 MHz, DMSO) δ 172.30, 171.57, 163.15, 154.63 (d, J = 12.2 Hz), 152.59 (d, J = 12.2 Hz), 150.42 (d, J = 13.5 Hz), 148.42 (d, J = 13.5 Hz), 132.83, 126.78 (dd, J = 8.3, 3.5 Hz), 119.28 (d, J = 18.9 Hz), 118.71 (d, J = 20.9 Hz), 105.64, 23.66, 13.30. ^{19}F NMR (471 MHz, DMSO) δ -127.04, -134.03. HRMS (ESI) calcd for $C_{12}H_{11}O_3N_2F_2S_2$ [M+H]$^+$: 333.0174, found 333.0176.

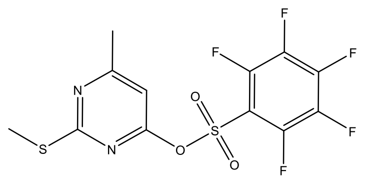

6-methyl-2-(methylthio)pyrimidin-4-yl-pentafluorobenzenesulfonate(A_5): White solid，m.p.74-76℃；Yield：50%, ^{1}H NMR (500 MHz, CDCl$_3$) δ 6.60 (s, 1H, Pyrimidine-H), 2.49 (s, 3H, Pyrimidine-CH$_3$), 2.35 (s, 3H, -SCH$_3$). ^{13}C NMR (100 MHz, CDCl$_3$) δ 173.14, 172.20, 163.91, 104.76, 24.45, 13.77. ^{19}F NMR (376 MHz, CDCl$_3$) δ -132.98, -142.21, -158.02. HRMS (ESI) calcd for $C_{12}H_8O_3N_2F_5S_2$ [M+H]$^+$: 386.9897, found 386.9892.

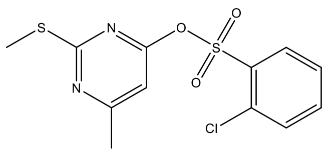

6-methyl-2-(methylthio)pyrimidin-4-yl-2-chlorobenzenesulfonate(A_6): White solid，m.p.76-78℃；Yield：70%, ^{1}H NMR (500 MHz, CDCl$_3$) δ 8.18 – 8.12 (m, 1H, Ph-H), 7.65 – 7.55 (m, 2H, Ph-H), 7.46 (ddd, *J* = 8.1, 6.8, 1.9 Hz, 1H, Ph-H), 6.60 (s, 1H, Pyrimidine-H), 2.45 (s, 3H, -SCH$_3$), 2.15 (s, 3H, Pyrimidine-CH$_3$). ^{13}C NMR (125 MHz, CDCl$_3$) δ 172.96, 171.48, 163.84, 135.70, 135.24, 133.19, 132.18, 132.15, 127.12, 105.07, 24.26, 13.92. HRMS (ESI) calcd for $C_{12}H_{12}O_3N_2ClS_2$ [M+H]$^+$: 330.9972, found 330.9975.

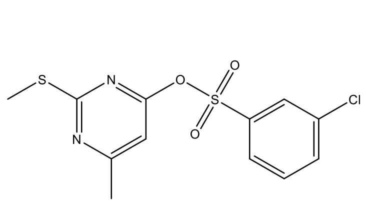

6-methyl-2-(methylthio)pyrimidin-4-yl-3-chloroben

zenesulfonate(A_7): White solid, m.p.62-64°C; Yield: 78%, ^{1}H NMR (400 MHz, $CDCl_3$) δ 8.07 (t, *J* = 1.9 Hz, 1H, Ph-H), 7.97 – 7.89 (m, 1H, Ph-H), 7.70 – 7.62 (m, 1H, Ph-H), 7.53 (t, *J* = 8.0 Hz, 1H, Ph-H), 6.57 (s, 1H, Pyrimidine-H), 2.46 (s, 3H, -SCH_3), 2.34 (s, 3H, Pyrimidine-CH_3). ^{13}C NMR (100 MHz, $CDCl_3$) δ 173.03, 171.51, 163.80, 138.60, 135.39, 134.65, 130.53, 128.99, 126.87, 105.20, 24.32, 14.12. HRMS (ESI) calcd for $C_{12}H_{12}O_3N_2ClS_2$ $[M+H]^+$: 330.9978, found 330.9975.

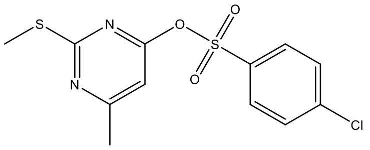

6-methyl-2-(methylthio)pyrimidin-4-yl-4-chlorobenzenesulfonate(A_8): White solid, m.p.71-73°C; Yield: 72%, ^{1}H NMR (500 MHz, $CDCl_3$) δ 8.09 – 7.95 (m, 2H, Ph-H), 7.62 – 7.50 (m, 2H, Ph-H), 6.57 (s, 1H, Pyrimidine-H), 2.46 (s, 3H, -SCH_3), 2.36 (s, 3H, Pyrimidine-CH_3). ^{13}C NMR (125 MHz, $CDCl_3$) δ 172.87, 171.40, 163.74, 141.36, 135.30, 130.31, 129.55, 105.19, 24.24, 14.07. HRMS (ESI) calcd for $C_{12}H_{12}O_3N_2ClS_2$ $[M+H]^+$: 330.9972, found 330.9975.

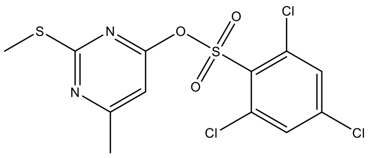

6-methyl-2-(methylthio)pyrimidin-4-yl-2,4,6-trichlorobenzenesulfonate(A_9): White solid, m.p.140-142°C; Yield: 81%, ^{1}H NMR (400 MHz, $CDCl_3$) δ 7.53 (s, 2H, Ph-H), 6.58 (s, 1H, Pyrimidine-H), 2.47 (s, 3H, -SCH_3), 2.22 (s, 3H, Pyrimidine-CH_3). ^{13}C NMR (100 MHz, $CDCl_3$) δ 172.94, 171.67, 163.96, 139.81, 136.64, 133.27, 131.24, 104.60, 24.37, 13.75. HRMS (ESI) calcd for $C_{12}H_{10}O_3N_2Cl_3S_2$ $[M+H]^+$: 398.9198, found 398.9198.

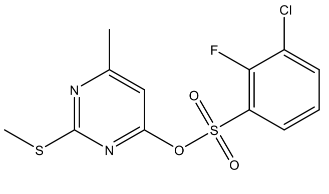

6-methyl-2-(methylthio)pyrimidin-4-yl-3-chloro-2-fluorobenzenesulfonate(A_{10}): White solid, m.p.85-87°C; Yield: 77%, ^{1}H NMR (400 MHz, $CDCl_3$) δ 7.89 (ddd, *J* = 7.8, 5.9, 1.7 Hz, 1H, Ph-H), 7.75 (ddd, *J* = 8.2, 6.6, 1.7 Hz, 1H, Ph-H), 7.35 – 7.25 (m, 1H, Ph-H), 6.59 (s, 1H, Pyrimidine-H),

2.47 (s, 3H, -SCH_3), 2.21 (s, 3H, Pyrimidine-CH_3). ^{13}C NMR (100 MHz, $CDCl_3$) δ 173.04, 171.76, 163.74, 155.15 (d, J = 261.0 Hz), 137.08, 129.39, 127.24 (d, *J* = 13.8 Hz), 124.80 (d, *J* = 5.0 Hz, 123.54 (d, *J* = 17.4 Hz) 105.25, 24.33, 13.97. ^{19}F NMR (376 MHz, $CDCl_3$) δ -108.44. HRMS (ESI) calcd for $C_{12}H_{11}O_3N_2ClFS_2$ [M+H]$^+$: 348.9884, found 348.9882.

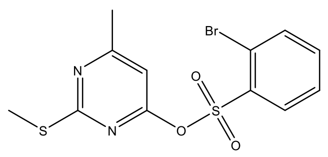

6-methyl-2-(methylthio)pyrimidin-4-yl-2-bromobenzenesulfonate(A_{11}): White solid，m.p.90-92°C；Yield：73%, ^{1}H NMR (400 MHz, $CDCl_3$) δ 8.22 – 8.15 (m, 1H, Ph-H), 7.84 – 7.76 (m, 1H, Ph-H), 7.55 – 7.47 (m, 2H, Ph-H), 6.61 (s, 1H, Pyrimidine-H), 2.46 (s, 3H, -SCH_3), 2.16 (s, 3H, Pyrimidine-CH_3). ^{13}C NMR (100 MHz, $CDCl_3$) δ 172.96, 171.46, 163.86, 137.54, 135.73, 135.16, 132.52, 127.70, 121.08, 105.05, 24.27, 13.96. HRMS (ESI) calcd for $C_{12}H_{12}O_3N_2BrS_2$ [M+H]$^+$: 374.9467, found 374.9472.

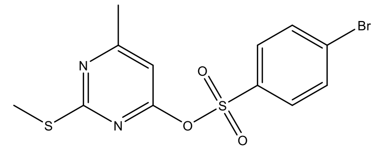

6-methyl-2-(methylthio)pyrimidin-4-yl-4-bromobenzenesulfonate(A_{12}): White solid，m.p.74-76°C；Yield：60%, ^{1}H NMR (500 MHz, $CDCl_3$) δ 7.94 – 7.87 (m, 2H, Ph-H), 7.76 – 7.69 (m, 2H, Ph-H), 6.57 (s, 1H, Pyrimidine-H), 2.45 (s, 3H, -SCH_3), 2.35 (s, 3H, Pyrimidine-CH_3). ^{13}C NMR (125 MHz, $CDCl_3$) δ 172.90, 171.45, 163.73, 135.87, 132.55, 130.34, 129.97, 105.20, 24.29, 14.08. HRMS (ESI) calcd for $C_{12}H_{12}O_3N_2BrS_2$ [M+H]$^+$: 374.9467, found 374.9471.

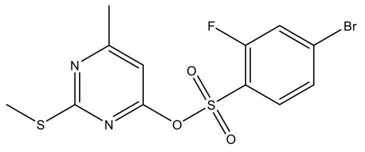

6-methyl-2-(methylthio)pyrimidin-4-yl-4-bromo-2-fluorobenzenesulfonate(A_{13}): White solid，m.p.84-86°C；Yield：70%, ^{1}H NMR (400 MHz, $CDCl_3$) δ 7.90 – 7.81 (m, 1H, Ph-H), 7.54 – 7.44 (m, 2H, Ph-H), 6.60 (s, 1H, Pyrimidine-H), 2.47 (s, 3H, -SCH_3), 2.25 (s, 3H, Pyrimidine-CH_3). ^{13}C NMR (100 MHz, $CDCl_3$) δ 173.01, 171.68, 163.75, 159.05 (d, *J* = 90.0 Hz), 132.13, 130.77 (d, *J* = 9.0 Hz), 128.07 (d, *J* = 3.9 Hz), 124.78 (d, *J* = 13.8 Hz), 121.33 (d, *J* = 24.

0 Hz), 105.21, 24.32, 13.99. ^{19}F NMR (376 MHz, $CDCl_3$) δ -104.44. HRMS (ESI) calcd for $C_{12}H_{11}O_3N_2BrFS_2$ $[M+H]^+$: 392.9379, found 392.9378.

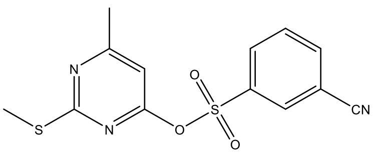

6-methyl-2-(methylthio)pyrimidin-4-yl-3-cyanobenzenesulfonate（A_{14}）: Yellowish solid，m.p.88-90°C；Yield：78%, ^{1}H NMR (400 MHz, $CDCl_3$) δ 8.07 (t, J = 1.9 Hz, 1H, Ph-H), 7.97 – 7.89 (m, 1H, Ph-H), 7.70 – 7.62 (m, 1H, Ph-H), 7.53 (t, J = 8.0 Hz, 1H, Ph-H), 6.57 (s, 1H, Pyrimidine-H), 2.46 (s, 3H, -SCH_3), 2.34 (s, 3H, Pyrimidine-CH_3). ^{13}C NMR (100 MHz, $CDCl_3$) δ 173.03, 171.51, 163.80, 138.60, 135.39, 134.65, 130.53, 128.99, 126.87, 105.20, 24.32, 14.12. HRMS (ESI) calcd for $C_{13}H_{12}O_3N_3S_2$ $[M+H]^+$: 322.0320, found 322.03 17.

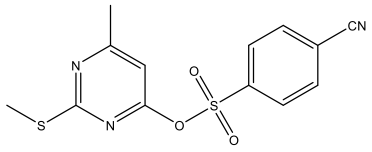

6-methyl-2-(methylthio)pyrimidin-4-yl-4-cyanobenzenesulfonate(A_{15}): White solid，m.p.112-114°C；Yie ld：77%, ^{1}H NMR (400 MHz, $CDCl_3$) δ 8.18 (d, J = 8.5 Hz, 2H, Ph-H), 7.88 (d, J = 8.4 Hz, 2H, Ph-H), 6.57 (s, 1H, Pyrimidi ne-H), 2.46 (s, 3H, -SCH_3), 2.32 (s, 3H, Pyrimidine-CH_3). ^{13}C NMR (100 MH z, $CDCl_3$) δ 172.86, 171.74, 163.57, 141.12, 132.90 , 129.56, 118.10, 117.03, 1 05.11, 24.34, 14.06. HRMS (ESI) calcd for $C_{12}H_{12}O_3N_3S_2$ $[M+H]^+$: 322.0320, found 322.0317.

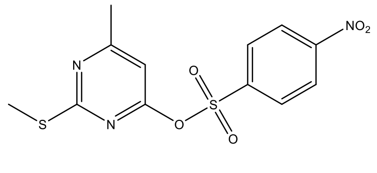

6-methyl-2-(methylthio)pyrimidin-4-yl-4-nitrobenzenesulfonate（A_{16}）: Yellow solid，m.p.94 -96°C；Yield：80%, ^{1}H NMR (400 MHz, $CDCl_3$) δ 8.49 – 8.37 (m, 2H, Ph-H), 8.33 – 8.21 (m, 2H, Ph-H), 6.58 (s, 1H, Pyrimid ine-H), 2.47 (s, 3H, -SCH_3), 2.34 (s, 3H, Pyrimidine-CH_3). ^{13}C NMR (100 MH z, $CDCl_3$) δ 172.92, 171.80, 163.60, 151.08, 142.63, 130.40, 124.35, 105.07, 2 4.38, 14.10. HRMS (ESI) calcd for $C_{12}H_{12}O_5N_3S_2$ $[M+H]^+$: 342.0213, found 3 42.0215.

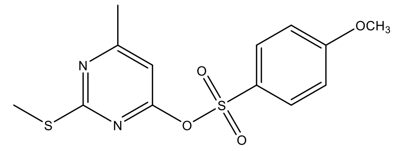

6-methyl-2-(methylthio)pyrimidin-4-yl-4-methoxybenzenesulfonate(A_{17}): White solid，m.p.58-60℃；Yield：60%,^{1}H NMR (400 MHz, $CDCl_3$) δ 8.03 – 7.92 (m, 2H, Ph-H), 7.07 – 6.96 (m, 2H, Ph-H), 6.57 (s, 1H, Pyrimidine-H), 3.89 (s, 3H, -OCH_3), 2.44 (s, 3H, -SCH_3), 2.39 (s, 3H, Pyrimidine-CH_3). ^{13}C NMR (100 MHz, $CDCl_3$) δ 172.82, 171.10, 164.49, 163.95, 131.28, 127.87, 114.41, 105.24, 55.95, 24.27, 14.20. HRMS (ESI) calcd for $C_{13}H_{15}O_4N_2S_2$ [M＋H]$^+$: 327.0472, found 327.0460.

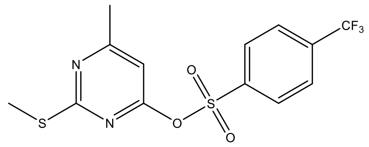

6-methyl-2-(methylthio)pyrimidin-4-yl-4-(trifluoromethyl)benzenesulfonate(A_{18}): White solid，m.p.74-76℃；Yield：78%, ^{1}H NMR (400 MHz, $CDCl_3$) δ 8.20 (d, *J* = 8.2 Hz, 2H, Ph-H), 7.85 (d, *J* = 8.3 Hz, 2H, Ph-H), 6.59 (s, 1H, Pyrimidine-H), 2.47 (s, 3H, -SCH_3), 2.29 (s, 3H, Pyrimidine-CH_3). ^{13}C NMR (100 MHz, CDCl3) δ 172.99, 171.67, 163.71, 140.59, 136.08(d, J = 33.0 Hz,), 129.50, 126.38 (q, *J* = 3.7 Hz) ,123.08(d, J = 272.0 Hz), 105.28, 24.35, 14.01. ^{19}F NMR (376 MHz, $CDCl_3$) δ -63.28. HRMS (ESI) calcd for $C_{13}H_{12}O_3N_2F_3S_2$ [M＋H]$^+$: 297.0368, found 297.0364.

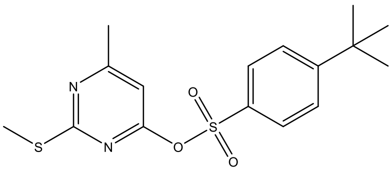

6-methyl-2-(methylthio)pyrimidin-4-yl-4-(tert-butyl)benzenesulfonate(A_{19}): White solid，m.p.60-62℃；Yield：82%, ^{1}H NMR (500 MHz, $CDCl_3$) δ 7.99 – 7.91 (m, 2H, Ph-H), 7.63 – 7.54 (m, 2H, Ph-H), 6.60 (s, 1H, Pyrimidine-H), 2.45 (s, 3H, -SCH_3), 2.29 (s, 3H, Pyrimidine-CH_3), 1.35 (s, 9H, -$(CH_3)_3$). ^{13}C NMR (125 MHz, CDCl3) δ 172.92, 171.23, 163.92, 158.73, 133.79, 128.64, 126.28, 105.45, 35.50, 31.12, 24.25, 14.06. HRMS (ESI) calcd for $C_{16}H_{21}O_3N_2S_2$ [M＋H]$^+$: 353.0988, found 353.0991.

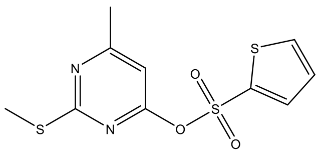

6-methyl-2-(methylthio)pyrimidin-4-yl-thiophene-2-

sulfonate(A$_{20}$): Yellowish solid，m.p.85-87°C；Yield：70%,^{1}H NMR (400 MHz, $CDCl_3$) δ 7.87 (dd, J = 3.9, 1.4 Hz, 1H, Thiophene-H), 7.78 (dd, J = 5.0, 1.4 Hz, 1H, Thiophene-H), 7.15 (dd, J = 5.0, 3.9 Hz, 1H, Thiophene-H), 6.58 (s, 1H, Pyrimidine-H), 2.46 (s, 3H, -SCH_3), 2.43 (s, 3H, Pyrimidine-CH_3). ^{13}C NMR (100 MHz, $CDCl_3$) δ 172.92, 171.37, 163.77, 136.27, 135.84, 135.33, 127.54, 105.23, 24.31, 14.22. HRMS (ESI) calcd for $C_{10}H_{11}O_3N_2S_3$ [M+H]$^+$: 302.9926, found 3302.9928.

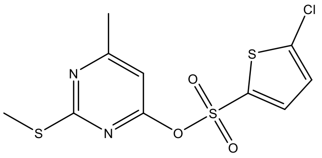

6-methyl-2-(methylthio)pyrimidin-4-yl-5-chlorothiophene-2-sulfonate(A$_{21}$): White solid，m.p.56-58°C；Yield：74%, ^{1}H NMR (400 MHz, $CDCl_3$) δ 7.68 (d, J = 4.1 Hz, 1H, Thiophene-H), 6.99 (d, J = 4.1 Hz, 1H, Thiophene-H), 6.56 (s, 1H, Pyrimidine-H), 2.47 (d, J = 2.8 Hz, 6H, -SCH_3, Pyrimidine-CH_3). ^{13}C NMR (100 MHz, $CDCl_3$) δ 172.91, 171.52, 163.72, 141.20, 135.94, 133.55, 126.85, 105.03, 24.36, 14.23. HRMS (ESI) calcd for $C_{10}H_{11}O_3N_2ClS_3$ [M+H]$^+$: 336.9537, found 336.9539.

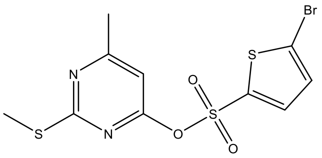

6-methyl-2-(methylthio)pyrimidin-4-yl-5-bromothiophene-2-sulfonate(A$_{22}$): Yellowish solid，m.p.64-66°C；Yield：70%,^{1}H NMR (400 MHz, $CDCl_3$) δ 7.64 (d, J = 4.1 Hz, 1H, Thiophene-H), 7.13 (d, J = 4.1 Hz, 1H, Thiophene-H), 6.56 (s, 1H, Pyrimidine-H), 2.47 (s, 6H, -SCH_3, Pyrimidine-CH_3). ^{13}C NMR (100 MHz, $CDCl_3$) δ 172.92, 171.51, 163.72, 136.55, 136.44, 130.45, 124.02, 105.05, 24.36, 14.23. HRMS (ESI) calcd for $C_{10}H_{11}O_3N_2BrS_3$ [M+H]$^+$: 380.9031, found 380.9036.

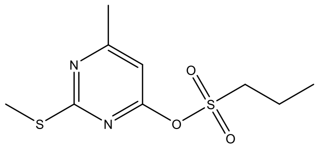

6-methyl-2-(methylthio)pyrimidin-4-yl-propane-1-sulfonate(A$_{23}$): White solid，m.p.56-58°C；Yield：74%, ^{1}H NMR (400 MHz, $CDCl_3$) δ 6.58 (s, 1H, Pyrimidine-H), 3.69 – 3.60 (m, 2H, -CH_2-), 2.53 (s, 3H, -SCH_3), 2.48 (s, 3H, Pyrimidine-CH_3), 2.11 – 1.96 (m, 2H,

-CH_2-), 1.12 (t, *J* = 7.5 Hz, 3H, -CH_3). ^{13}C NMR (100 MHz, $CDCl_3$) δ 172.93, 171.59, 164.01, 105.65, 55.94, 24.30, 17.43, 14.37, 12.95. HRMS (ESI) calcd for $C_9H_{15}O_3N_2S_2$ [M+H]$^+$: 263.0519, found 263.0520.

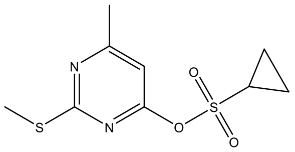

6-methyl-2-(methylthio)pyrimidin-4-yl-cyclopropanesulfonate(A_{24}): White solid，m.p.74-76°C；Yield：70%, ^1H NMR (400 MHz, $CDCl_3$) δ 6.57 (s, 1H, Pyrimidine-H), 3.15 (tt, *J* = 8.0, 4.8 Hz, 1H, -CH-), 2.55 (s, 3H, -SCH_3), 2.47 (s, 3H, Pyrimidine-CH_3), 1.47 – 1.41 (m, 2H, -CH_2-), 1.26 – 1.19 (m, 2H, -CH_2-). ^{13}C NMR (100 MHz, $CDCl_3$) δ 172.81, 171.35, 164.03, 105.09, 31.12, 24.31, 14.39, 7.11. HRMS (ESI) calcd for $C_9H_{13}O_3N_2S_2$ [M+H]$^+$: 261.0362, found 261.0364.

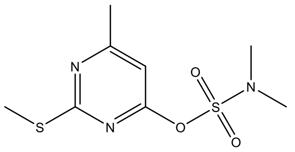

6-methyl-2-(methylthio)pyrimidin-4-yl-dimethylsulfamate (A_{25}): White solid，m.p.42-44°C；Yield：40%, ^1H NMR (400 MHz, $CDCl_3$) δ 6.62 (s, 1H, Pyrimidine-H), 3.04 (s, 6H, -$N(CH_3)_2$), 2.55 (s, 3H, -SCH_3), 2.47 (s, 3H, Pyrimidine-CH_3). ^{13}C NMR (100 MHz, $CDCl_3$) δ 172.83, 171.02, 164.23, 104.61, 38.89, 24.31, 14.33. HRMS (ESI) calcd for $C_8H_{14}O_3N_3S_2$ [M+H]$^+$: 264.0471, found 264.0473.

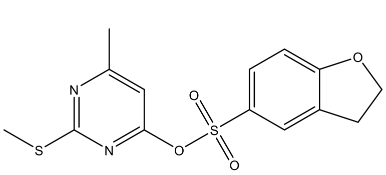

6-methyl-2-(methylthio)pyrimidin-4-yl-2,3-dihydrobenzofuran-5-sulfonate(A_{26}): Yellow solid，m.p.94-96°C；Yield：80%, ^1H NMR (400 MHz, $CDCl_3$) δ 7.89 – 7.79 (m, 2H, Ph-H), 6.90 – 6.83 (m, 1H, Ph-H), 6.57 (s, 1H, Pyrimidine-H), 4.71 (t, *J* = 8.9 Hz, 2H, furan-H), 3.29 (t, *J* = 8.8 Hz, 2H, furan-H), 2.42 (d, *J* = 13.6 Hz, 6H. -SCH_3, Pyrimidine-CH_3). ^{13}C NMR (100 MHz, $CDCl_3$) δ 172.74, 171.07, 165.52, 163.97, 131.02, 128.65, 127.60, 126.36, 109.67, 105.19, 72.76, 28.97, 24.28, 14.16. HRMS (ESI) calcd for $C_{14}H_{15}O_4N_2S_2$ [M+H]$^+$: 339.0468, found 339.0471.

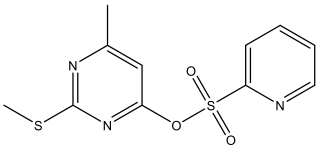

6-methyl-2-(methylthio)pyrimidin-4-yl-pyridine-2-sulfonate(A_{27}): Reddish brown solid，m.p.70-72℃; Yield：72%, ^{1}H NMR (400 MHz, $CDCl_3$) δ 9.24 (d, *J* = 1.9 Hz, 1H, Pyridine-H), 8.90 (dd, *J* = 4.9, 1.6 Hz, 1H, Pyridine-H), 8.34 (ddd, *J* = 8.1, 2.3, 1.7 Hz, 1H, Pyridine-H), 7.54 (ddd, *J* = 8.1, 4.9, 0.7 Hz, 1H, Pyridine-H), 6.58 (s, 1H, Pyrimidine-H), 2.46 (s, 3H, -SCH_3), 2.32 (s, 3H, Pyrimidine-CH_3). ^{13}C NMR (100 MHz, $CDCl_3$) δ 173.00, 171.66, 163.75, 154.82, 149.60, 136.62, 134.06, 123.76, 105.17, 24.34, 14.12. HRMS (ESI) calcd for $C_{11}H_{12}O_3N_3S_2$ $[M+H]^+$: 298.0315, found 298.0317.

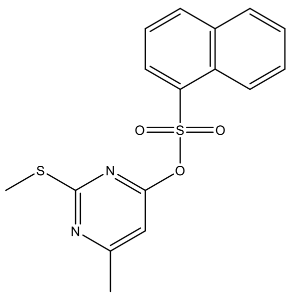

6-methyl-2-(methylthio)pyrimidin-4-yl-naphthalene-1-sulfonate(A_{28}): White solid，m.p.92-94℃; Yield：75%, ^{1}H NMR (400 MHz, $CDCl_3$) δ 8.67 (d, *J* = 8.7 Hz, 1H, Naphthalene-H), 8.36 (dd, *J* = 7.4, 1.1 Hz, 1H, Naphthalene-H), 8.17 (d, *J* = 8.3 Hz, 1H, Naphthalene-H), 7.97 (d, *J* = 8.1 Hz, 1H, Naphthalene-H), 7.81 – 7.69 (m, 1H, Naphthalene-H), 7.69 – 7.53 (m, 2H, Naphthalene-H), 6.59 (s, 1H, Pyrimidine-H), 2.41 (s, 3H, -SCH_3), 2.03 (s, 3H, Pyrimidine-CH_3). ^{13}C NMR (100 MHz, $CDCl_3$) δ 172.94, 171.21, 163.94, 136.09, 134.15, 132.65, 131.16, 129.17, 129.09, 128.47, 127.50, 124.79, 124.07, 105.23, 24.17, 13.87. HRMS (ESI) calcd for $C_{16}H_{15}O_3N_2S_2$ $[M+H]^+$: 347.0519, found 347.0522.

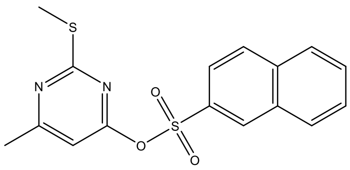

6-methyl-2-(methylthio)pyrimidin-4-yl-naphthalene-2-sulfonate(A_{29}): White solid，m.p.62-64℃; Yield：69%, ^{1}H NMR (400 MHz, $CDCl_3$) δ 8.62 (s, 1H, Naphthalene-H), 8.06 – 7.90 (m, 4H, Naphthalene-H), 7.75 – 7.60 (m, 2H, Naphthalene-H), 6.60 (s, 1H, Pyrimidine-H), 2.43 (s, 3H, -SCH_3), 2.20 (s, 3H, Pyrimidine-CH_3). ^{13}C NMR (100 MHz, $CDCl_3$) δ 172.90, 171.27, 163.93, 135.64, 133.65, 131.87, 130.99, 129.91, 129.56, 128.15, 128.08,

123.08, 105.23, 24.28, 14.00. HRMS (ESI) calcd for $C_{16}H_{15}O_3N_2S_2$ [M+H]$^+$: 347.0519, found 347.0522.

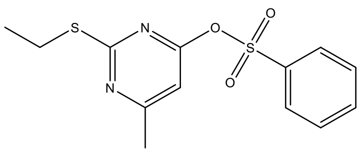

6-methyl-2-(ethylthio)pyrimidin-4-yl-benzenesulfonate(A_{30}): Yellow oily，Yield：69%, ^{1}H NMR (500 MHz, $CDCl_3$) δ 8.04 (dd, *J* = 8.4, 1.2 Hz, 2H, Ph-H), 7.72 – 7.67 (m, 1H, Ph-H), 7.58 (t, *J* = 7.9 Hz, 2H, Ph-H), 6.58 (s, 1H, Pyrimidine-H), 2.88 (q, *J* = 7.4 Hz, 2H, -CH_2-), 2.44 (s, 3H, Pyrimidine-CH_3), 1.25 (t, *J* = 7.4 Hz, 3H, -CH_3). ^{13}C NMR (100 MHz, $CDCl_3$) δ 172.71, 171.38, 163.92, 137.11, 134.51, 129.29, 128.67, 105.39, 25.29, 24.31, 14.38. HRMS (ESI) calcd for $C_{12}H_{12}O_5N_3S_2$ [M + H]$^+$: 311.0519, found 311.0515.

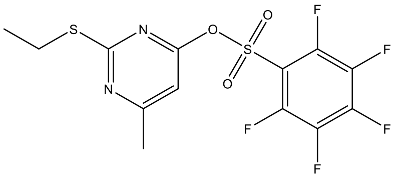

6-methyl-2-(ethylthio)pyrimidin-4-yl-pentafluorobenzenesulfonate(A_{31}): White solid，m.p.64-66℃；Yield：65%, ^{1}H NMR (400 MHz, $CDCl_3$) δ 6.59 (s, 1H, Pyrimidine-H), 2.91 (q, *J* = 7.4 Hz, 2H, -CH_2-), 2.49 (s, 3H, Pyrimidine-CH_3), 1.28 (t, *J* = 7.4 Hz, 3H, -CH_3). ^{13}C NMR (100 MHz, $CDCl_3$) δ 172.86, 172.24, 163.91, 104.79, 25.21, 24.47, 14.11. HRMS (ESI) calcd for $C_{12}H_{12}O_5N_3S_2$ [M + H]$^+$: 401.0048, found 401.0045.

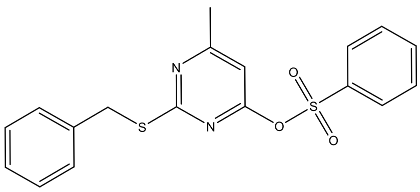

6-methyl-2-(benzylthio)pyrimidin-4-yl-benzenesulfonate(A_{32}): Yellow solid，m.p.88-90℃；Yield：75%, ^{1}H NMR (400 MHz, $CDCl_3$) δ 7.97 (d, *J* = 7.4 Hz, 2H, Ph-H), 7.59 (t, *J* = 7.5 Hz, 1H, Ph-H), 7.47 (t, *J* = 7.9 Hz, 2H, Ph-H), 7.26 – 7.17 (m, 5H, Ph-H), 6.54 (s, 1H, Pyrimidine-H), 4.09 (s, 2H, -CH_2-), 2.40 (s, 3H, Pyrimidine-CH_3). ^{13}C NMR (100 MHz, $CDCl_3$) δ 172.10, 171.48, 163.86, 137.01, 136.93, 134.58, 129.28, 129.18, 128.76, 128.65, 127.46, 105.72, 35.35, 24.29. HRMS (ESI) calcd for $C_{12}H_{12}O_5N_3S_2$ [M + H]$^+$: 373.0675, found 373.0672.

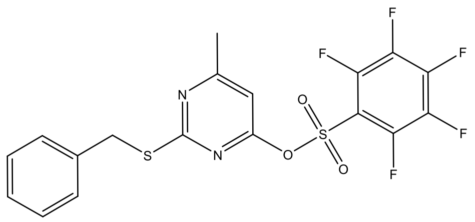

6-methyl-2-(benzylthio)pyrimidin-4-yl-pentafluorobenzenesulfonate(A_{33}): Yellow solid, m.p.82-84°C; Yield: 70%, ^{1}H NMR (500 MHz, $CDCl_3$) δ 7.26 – 7.15 (m, 5H, Ph-H), 6.64 (s, 1H, Pyrimidine-H), 4.16 (s, 2H, $-CH_2-$), 2.51 (s, 3H, Pyrimidine-CH_3). ^{13}C NMR (100 MHz, $CDCl_3$) δ 171.74, 171.11, 166.56, 136.76, 128.88, 128.69, 128.54, 128.24, 127.36, 101.82, 35.26, 24.31. HRMS (ESI) calcd for $C_{12}H_{12}O_5N_3S_2$ $[M+H]^+$: 463.0204, found 463.0200.

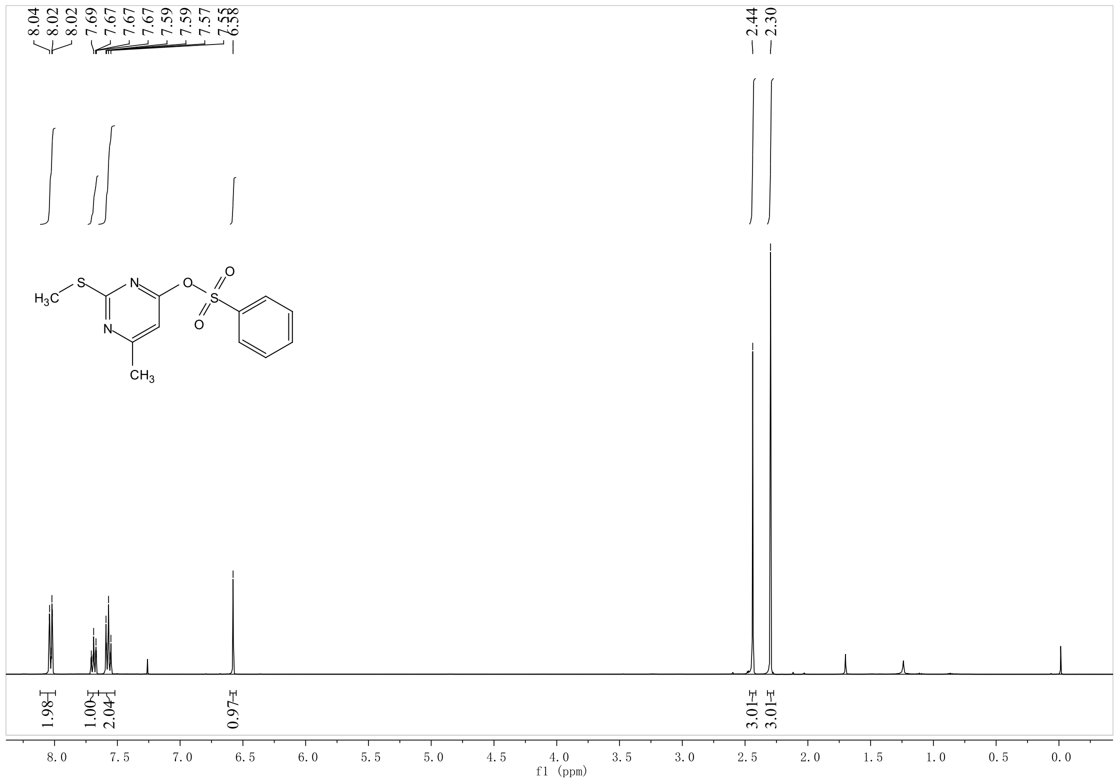

^{1}H NMR of compound A_1

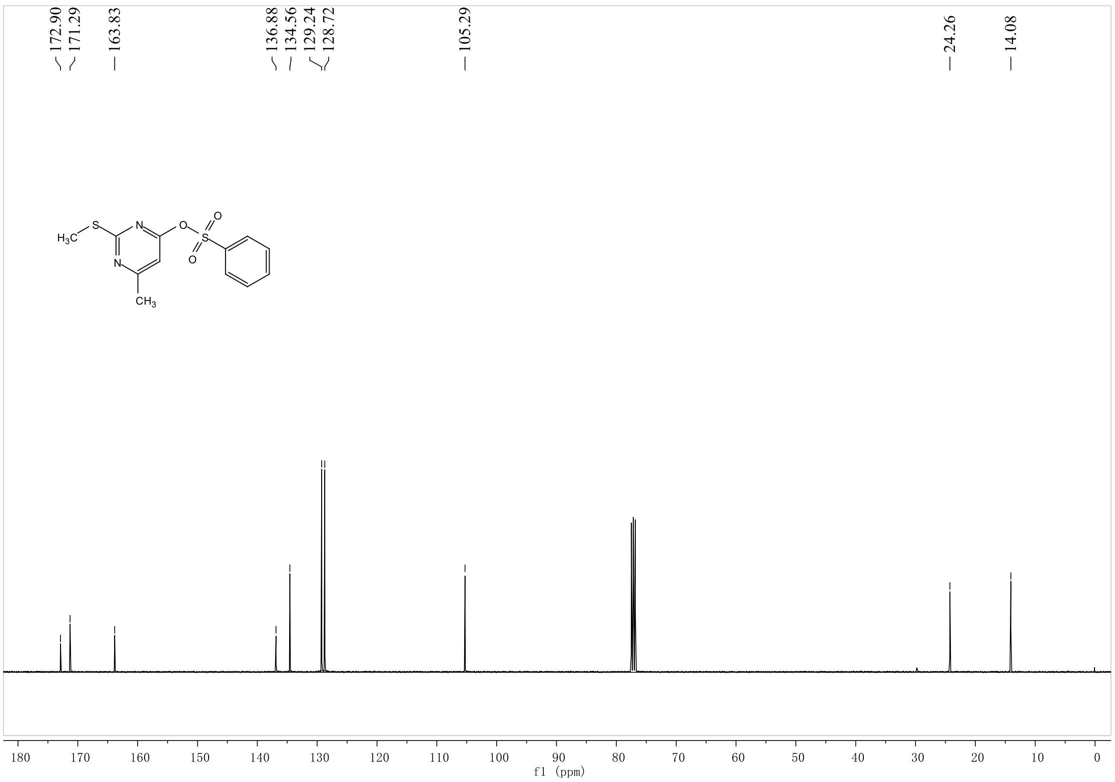

^{13}C NMR of compound A$_1$

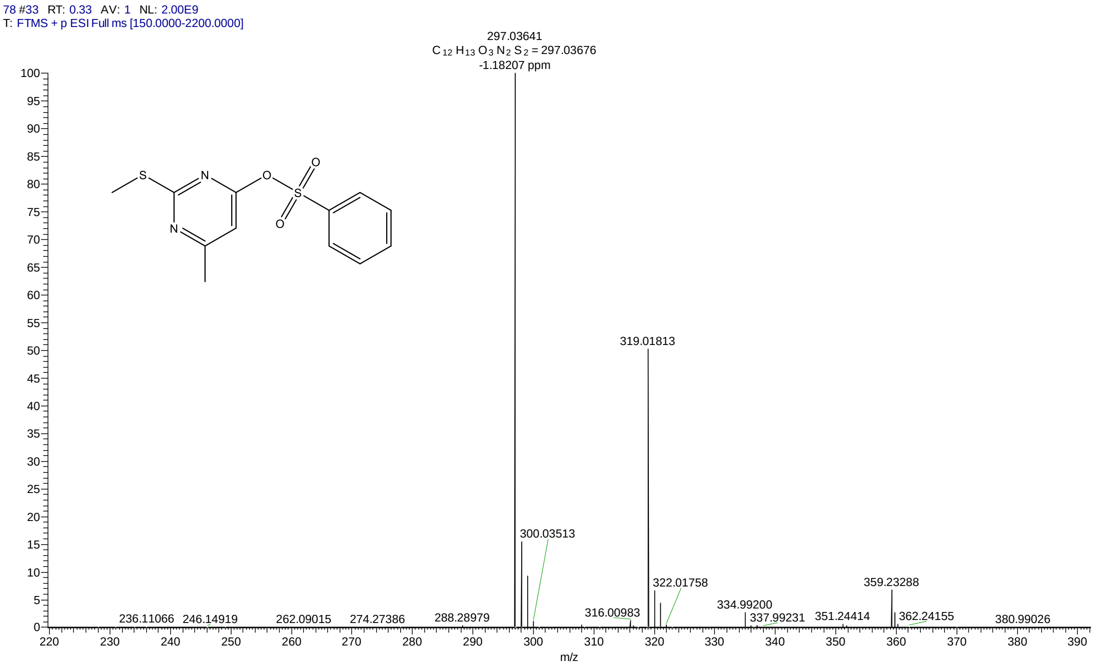

HRMS of compound A$_1$

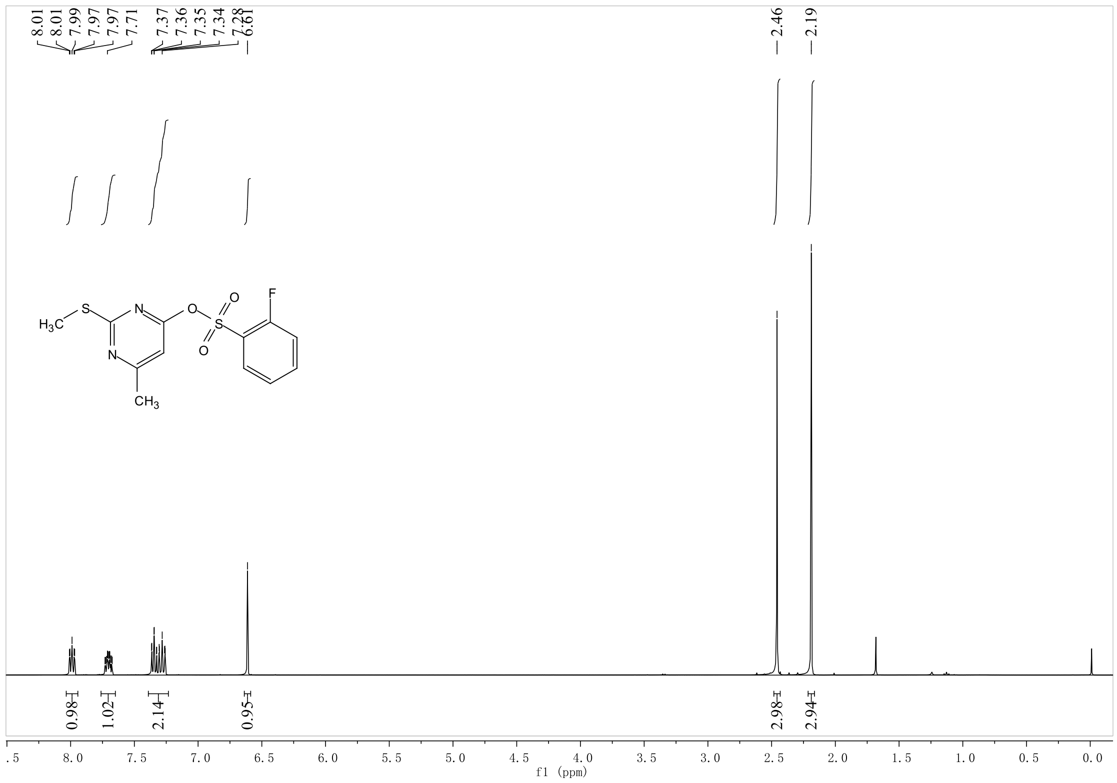

^{1}H NMR of compound A2

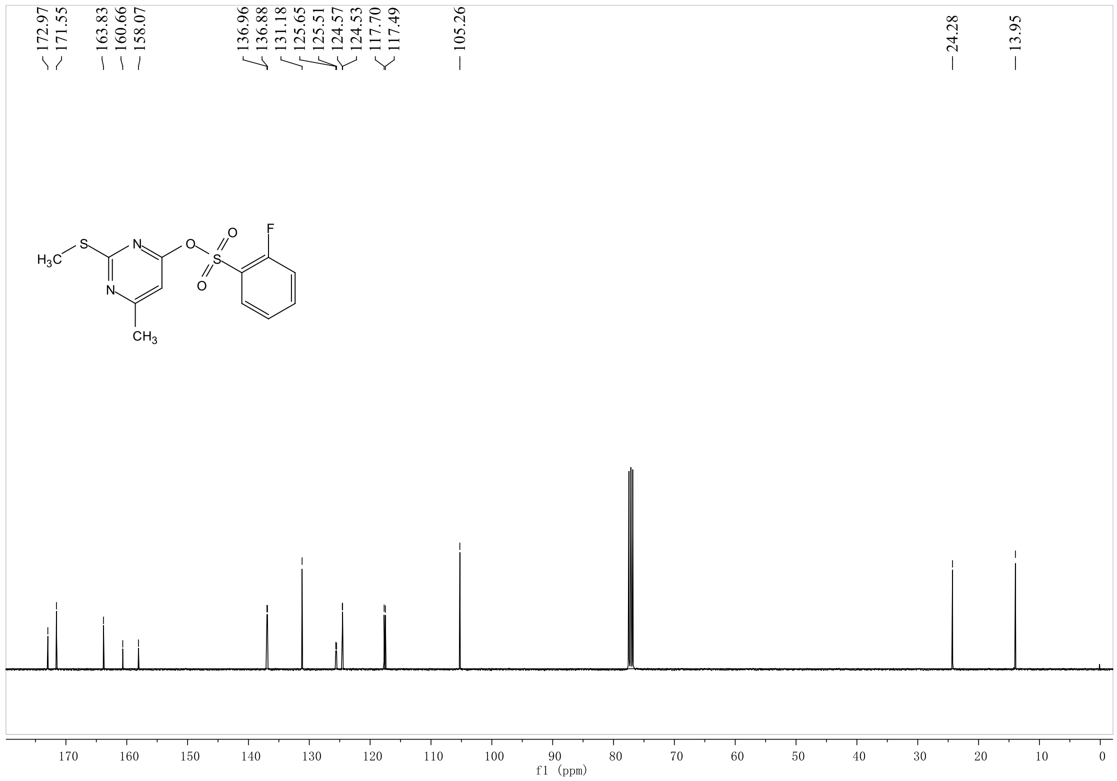

^{13}C NMR of compound A2

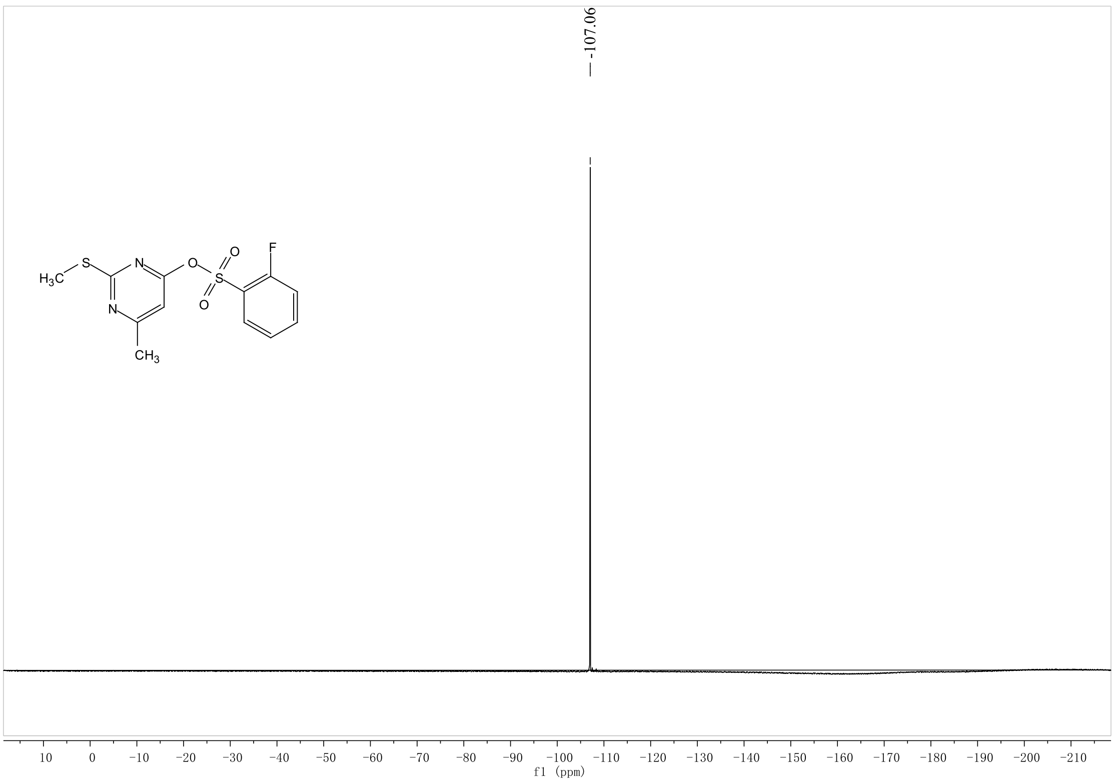

^{19}F NMR of compound A2

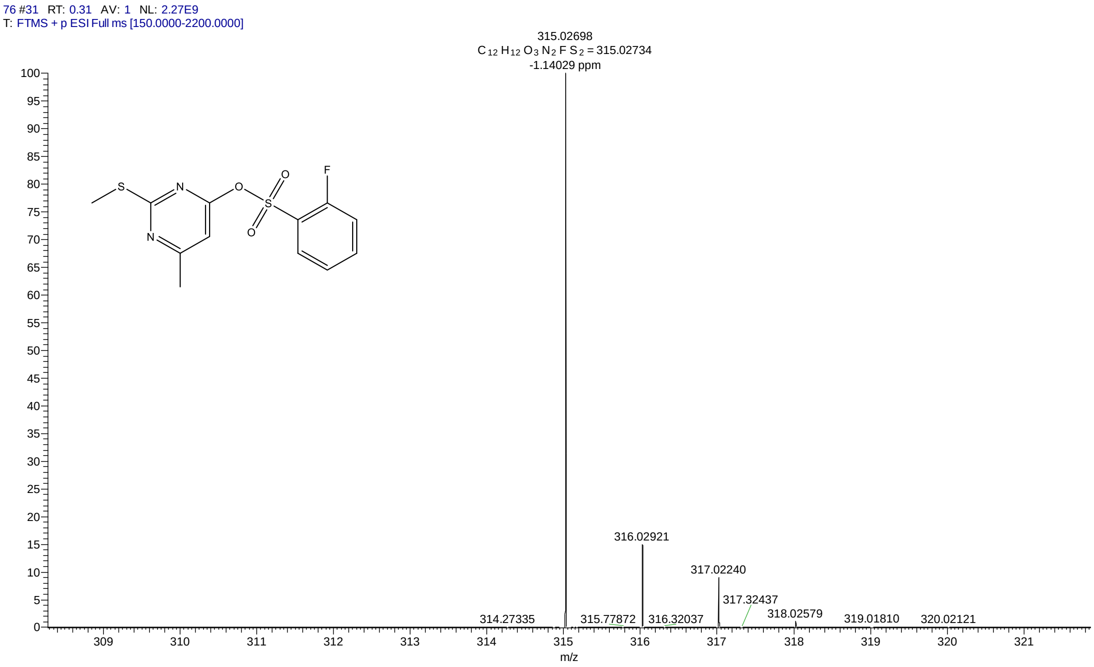

HRMS of compound A2

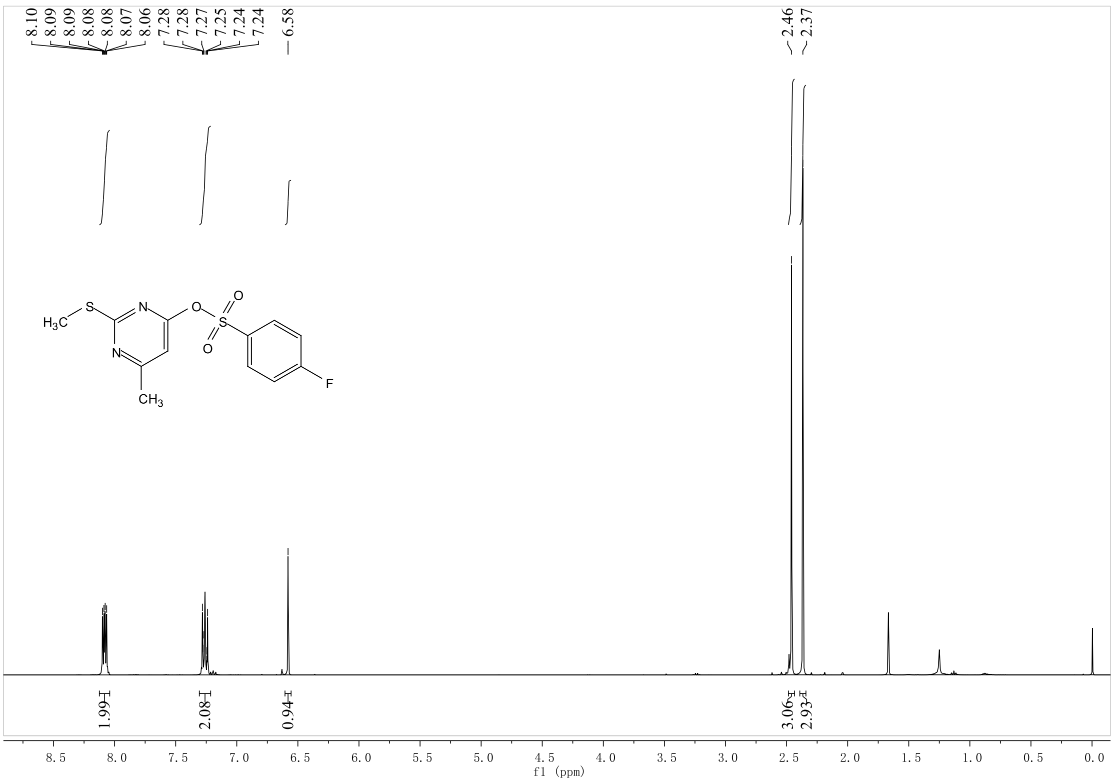

^{1}H NMR of compound A3

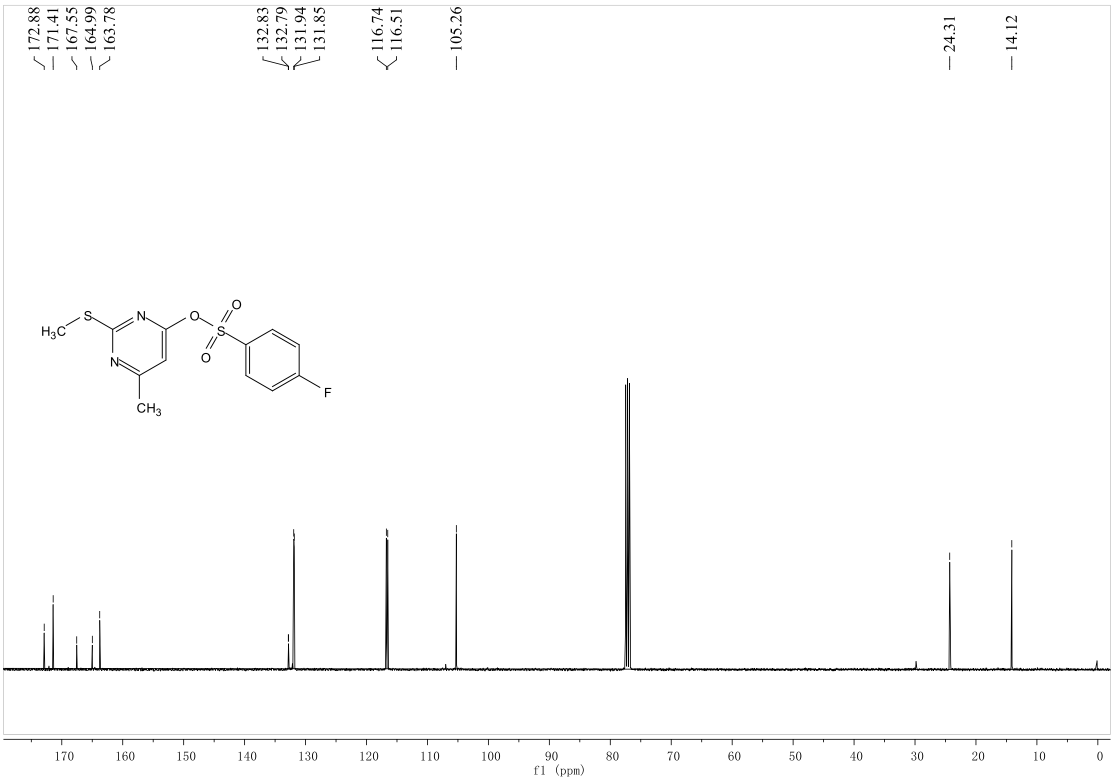

^{13}C NMR of compound A3

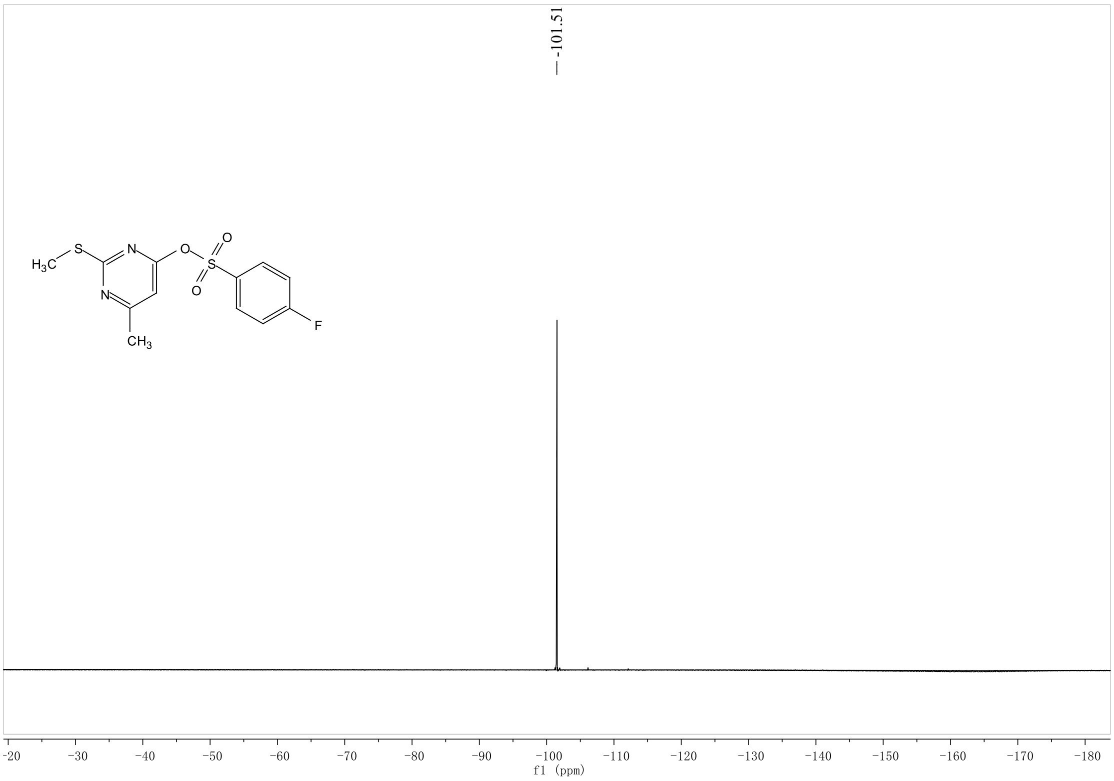

^{19}F NMR of compound A3

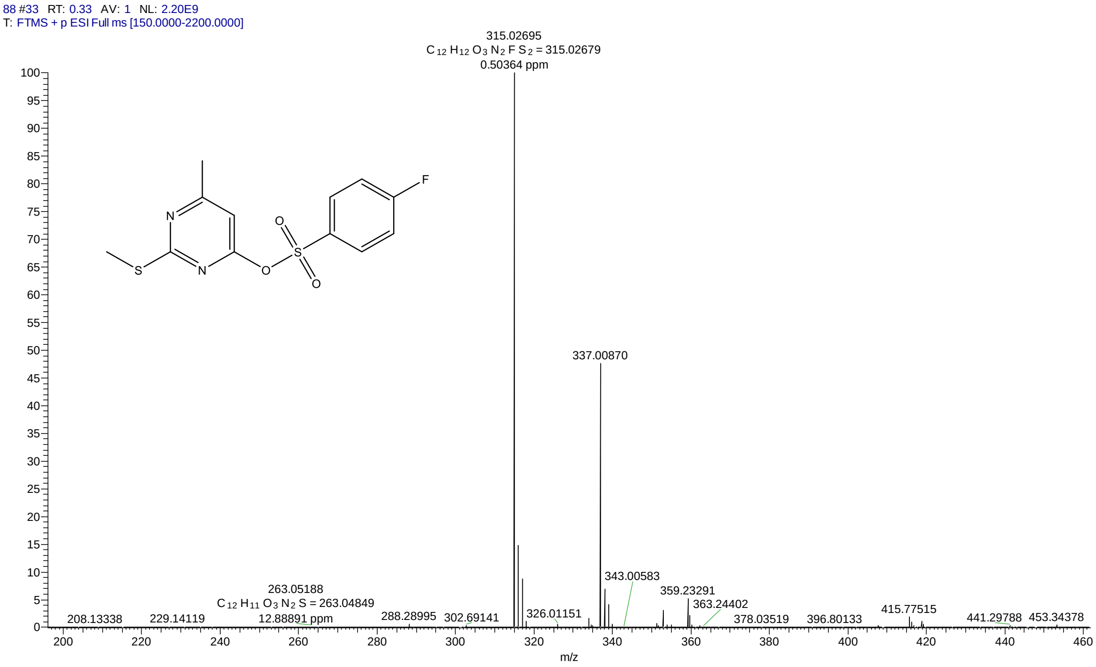

HRMS of compound A3

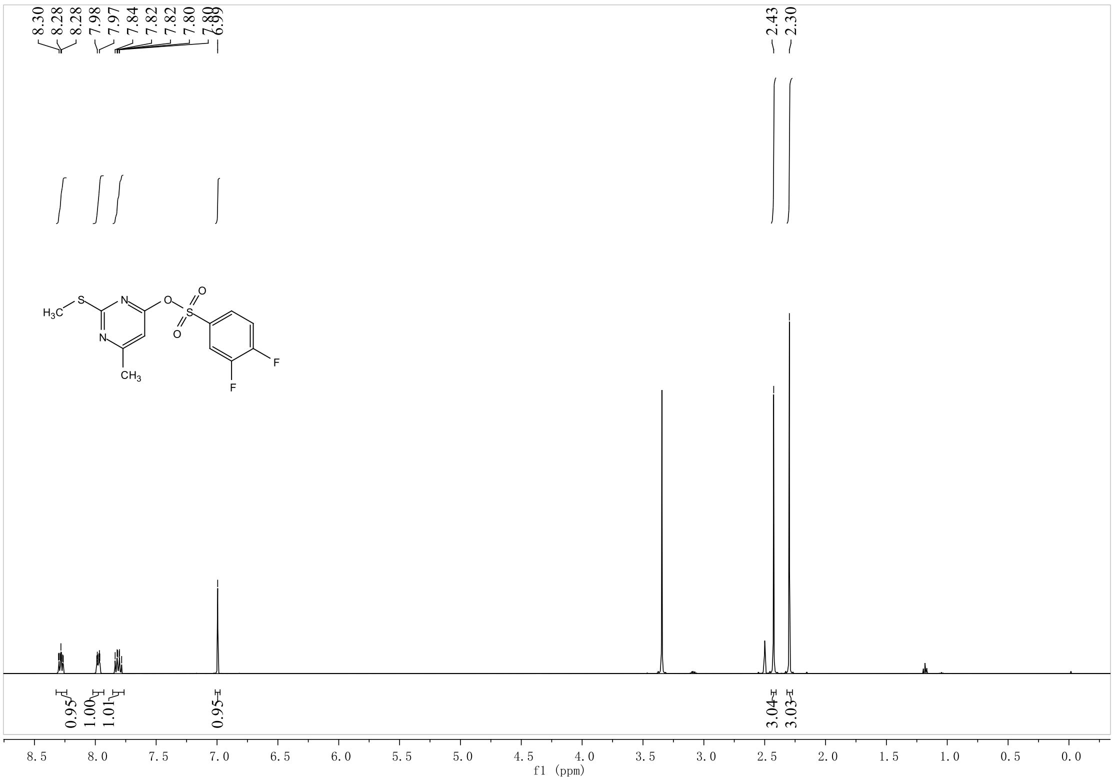

^{1}H NMR of compound A4

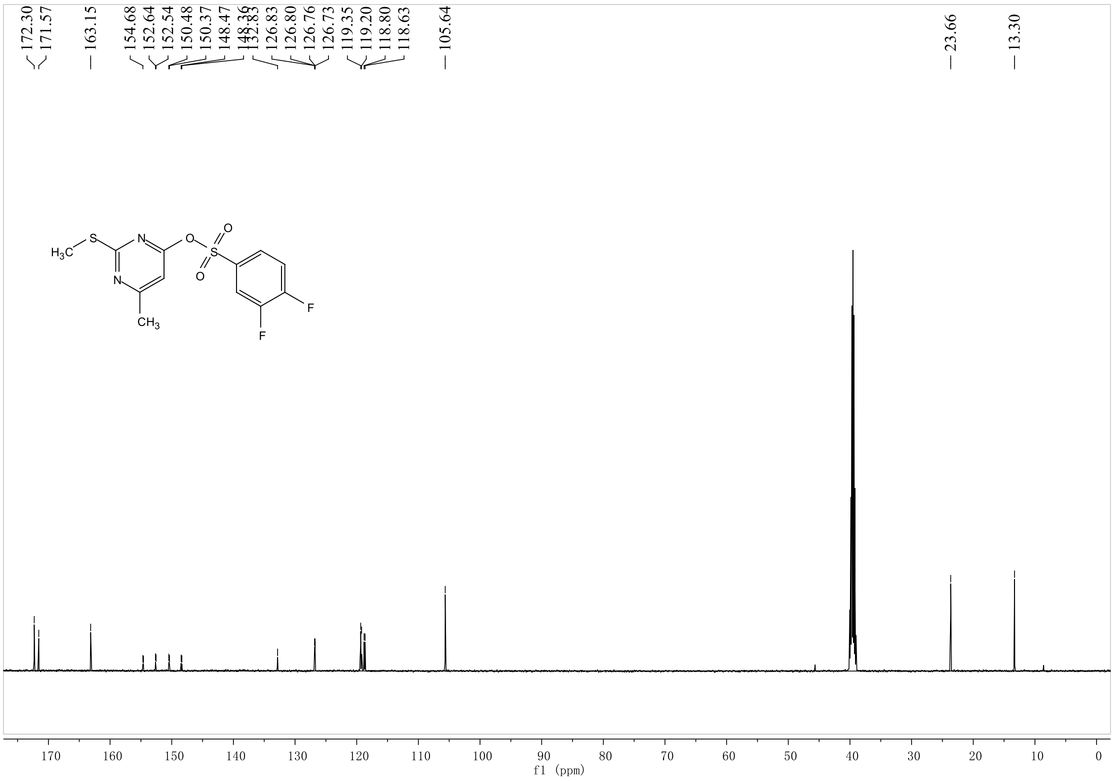

^{13}C NMR of compound A4

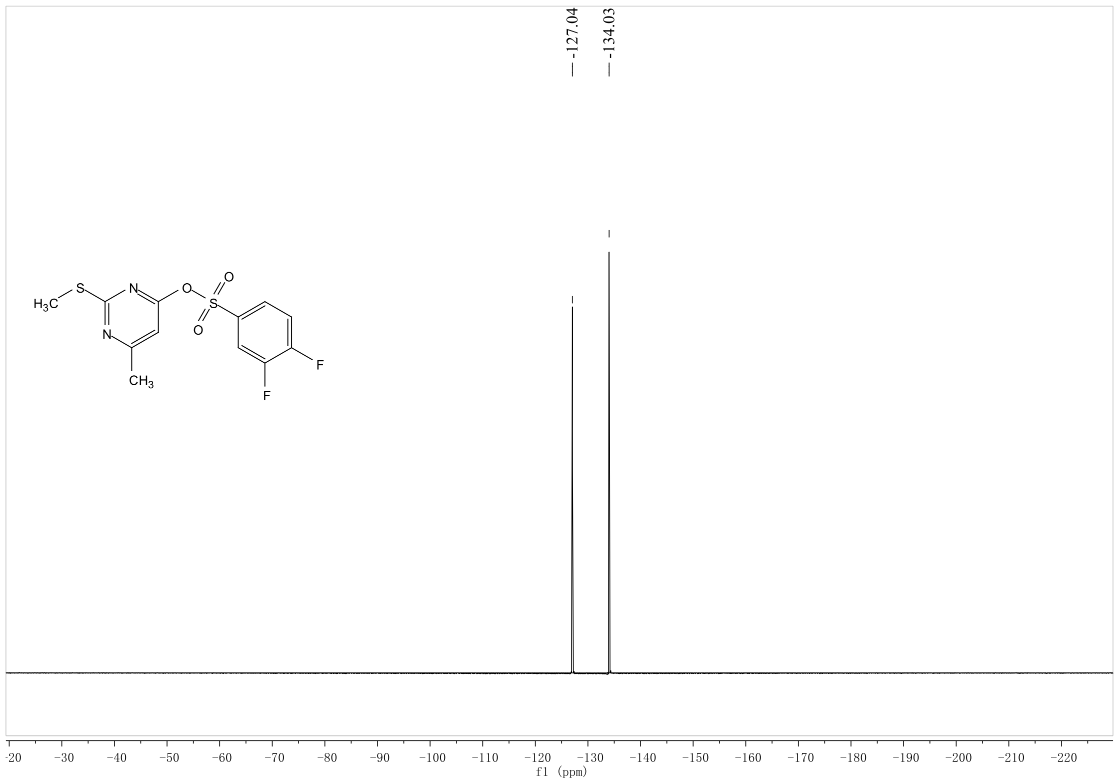

^{19}F NMR of compound A$_4$

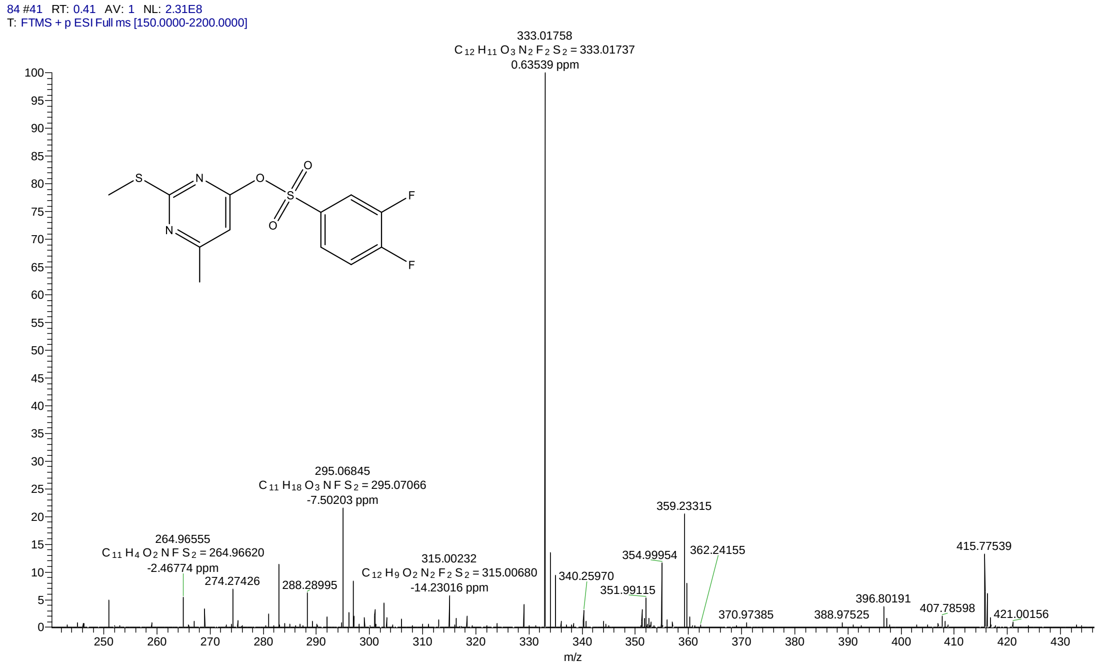

HRMS of compound A$_4$

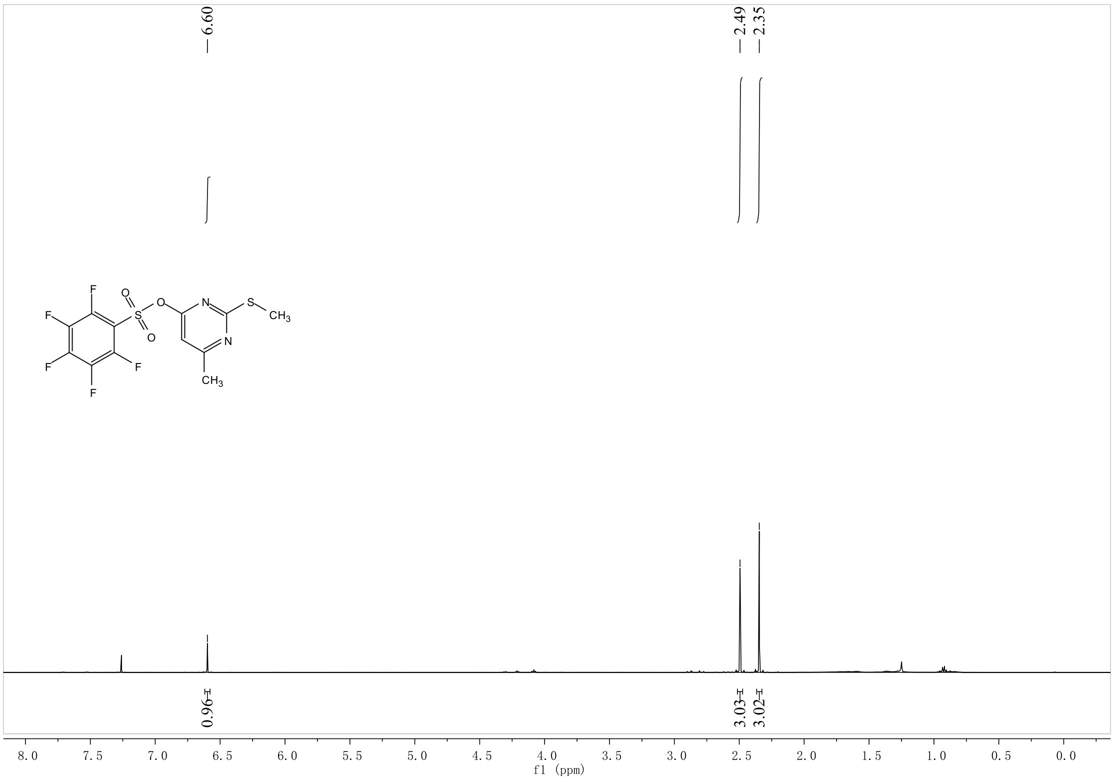

^{1}H NMR of compound A5

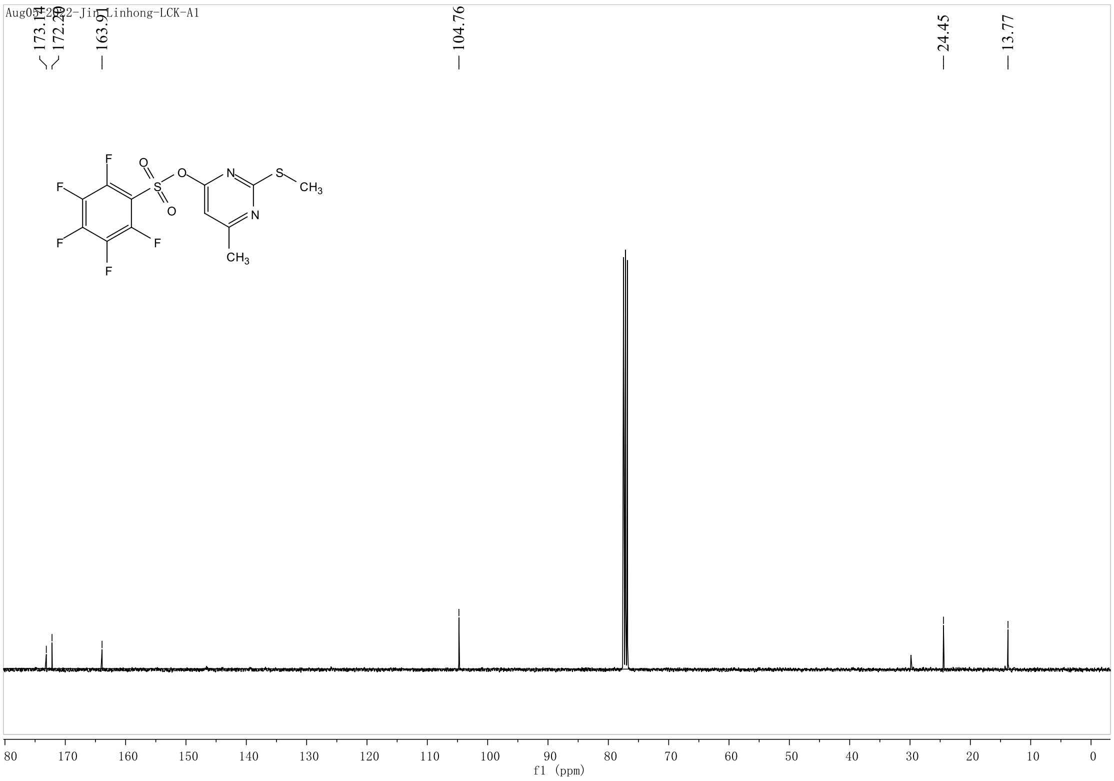

^{13}C NMR of compound A5

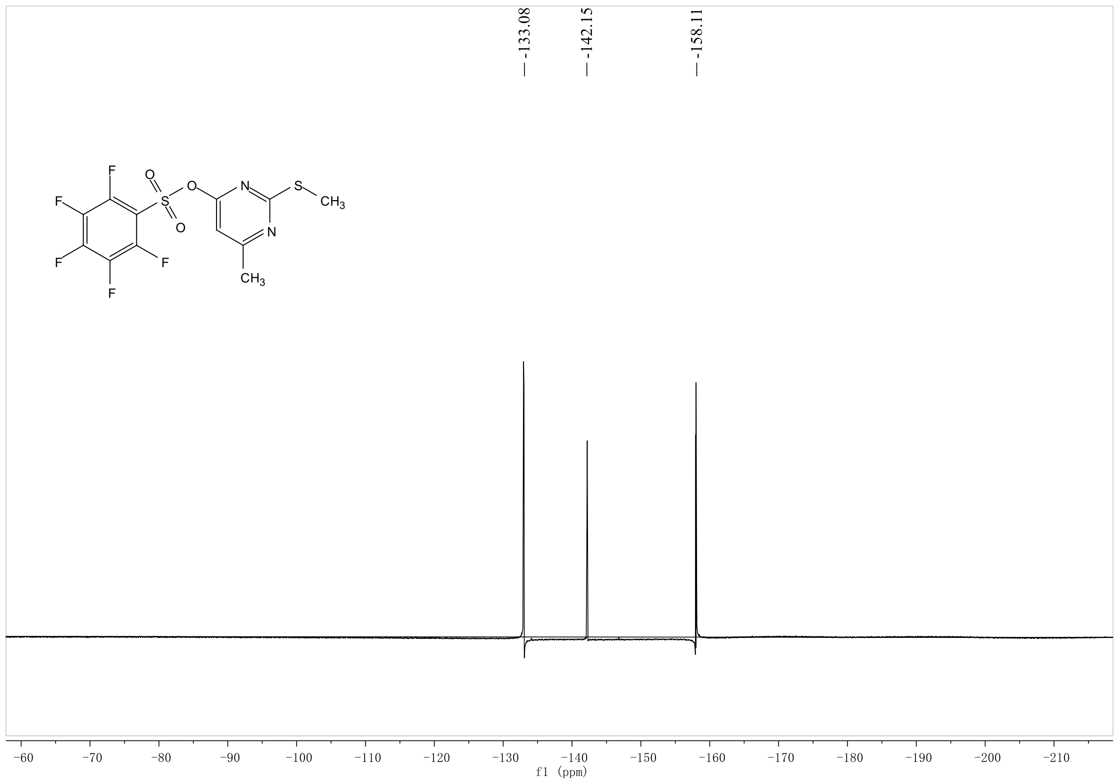

^{19}F NMR of compound A5

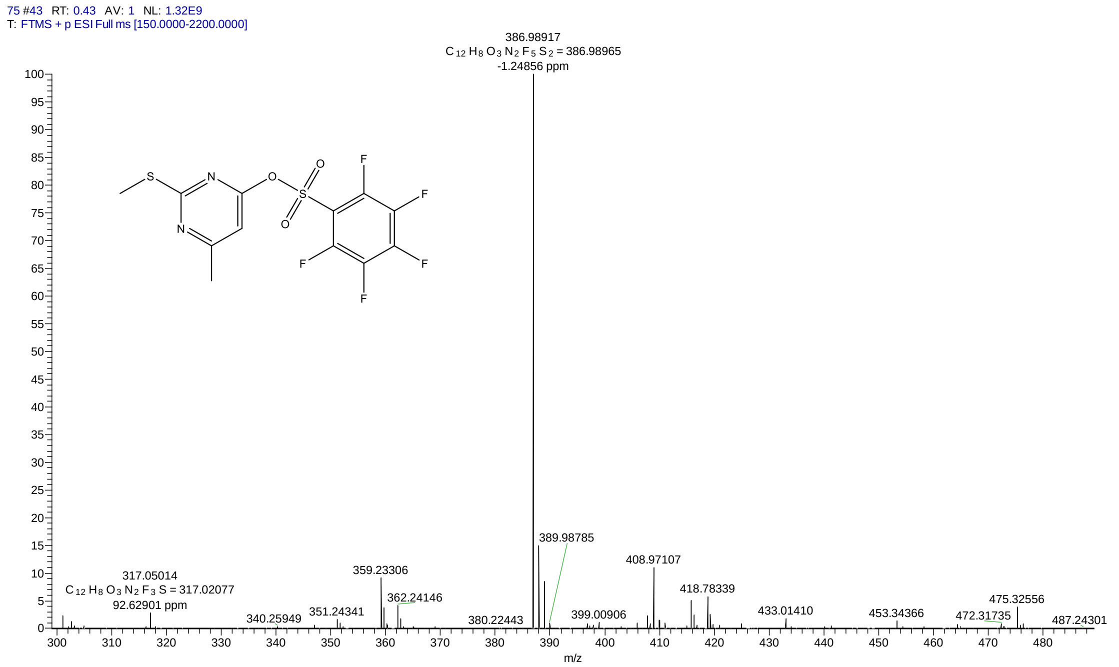

HRMS of compound A5

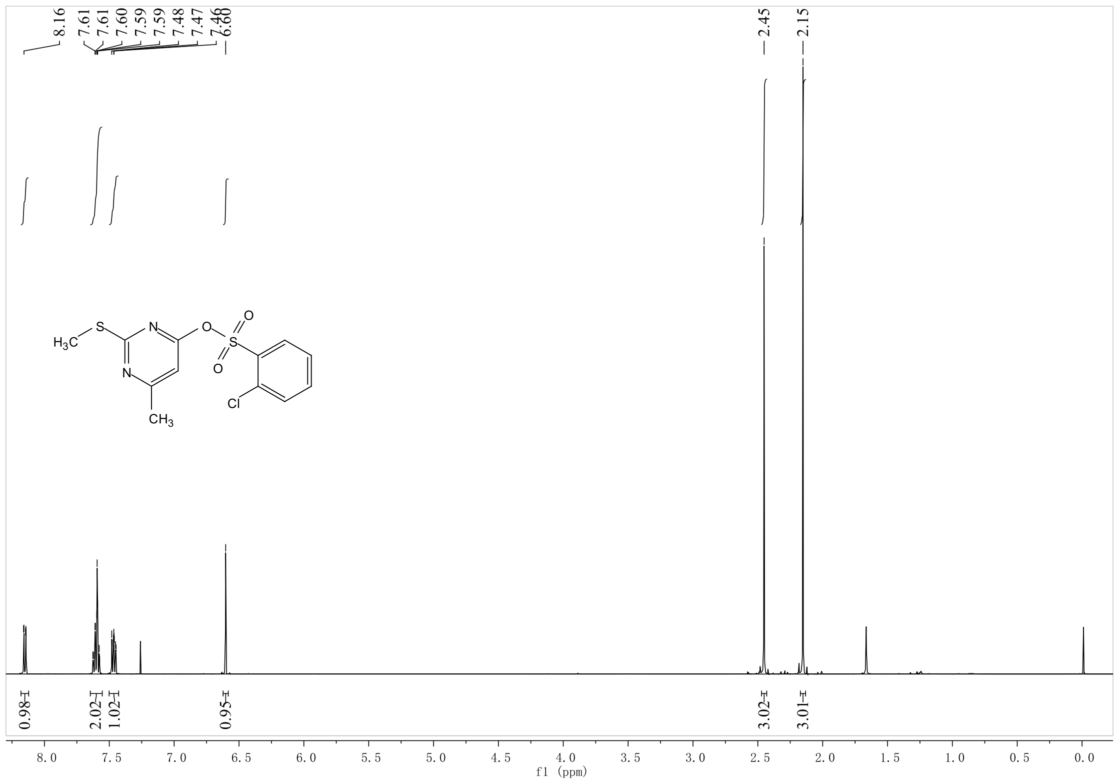

^{1}H NMR of compound A6

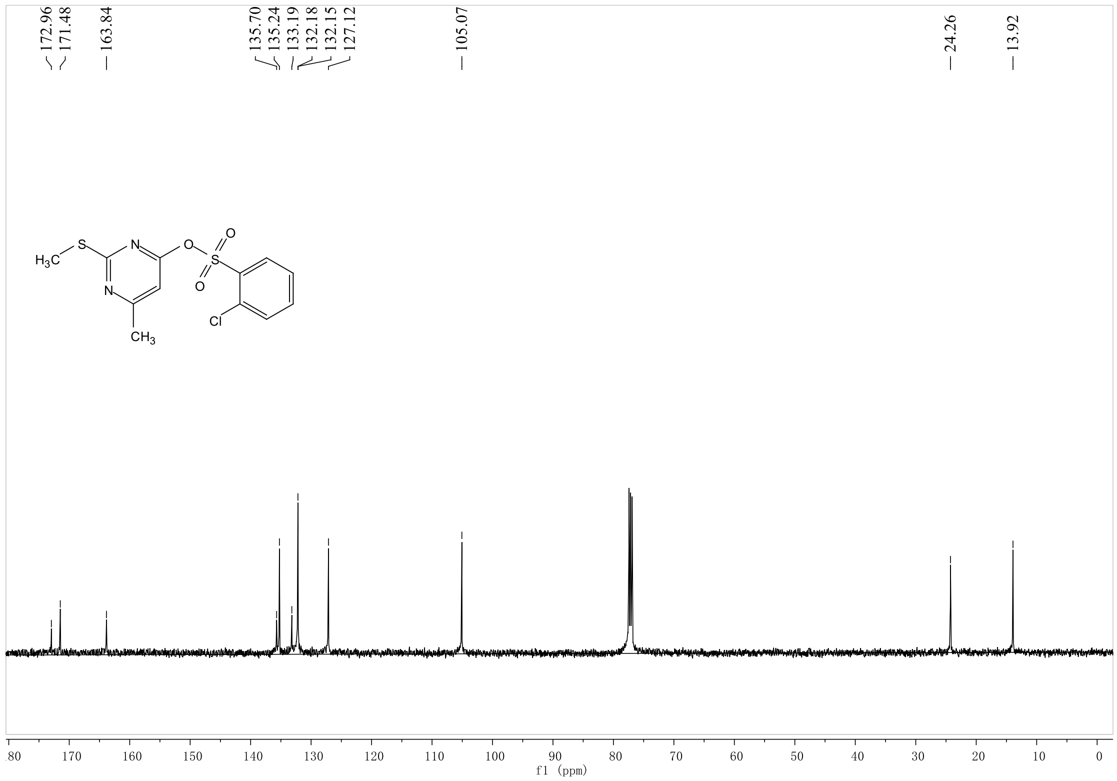

^{13}C NMR of compound A6

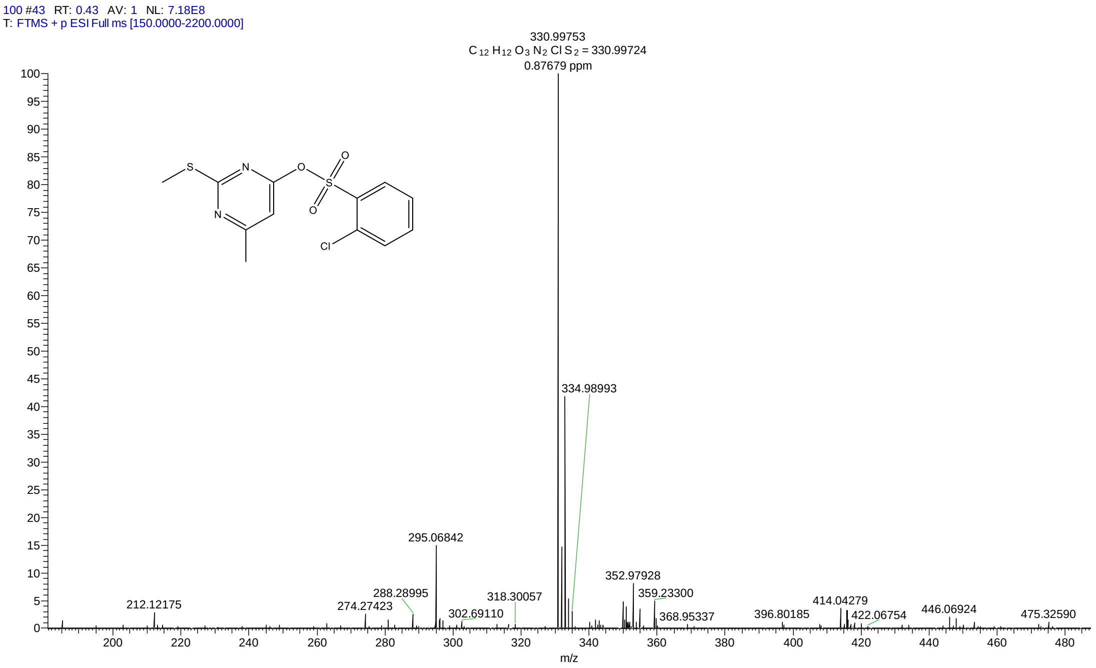

HRMS of compound A_6

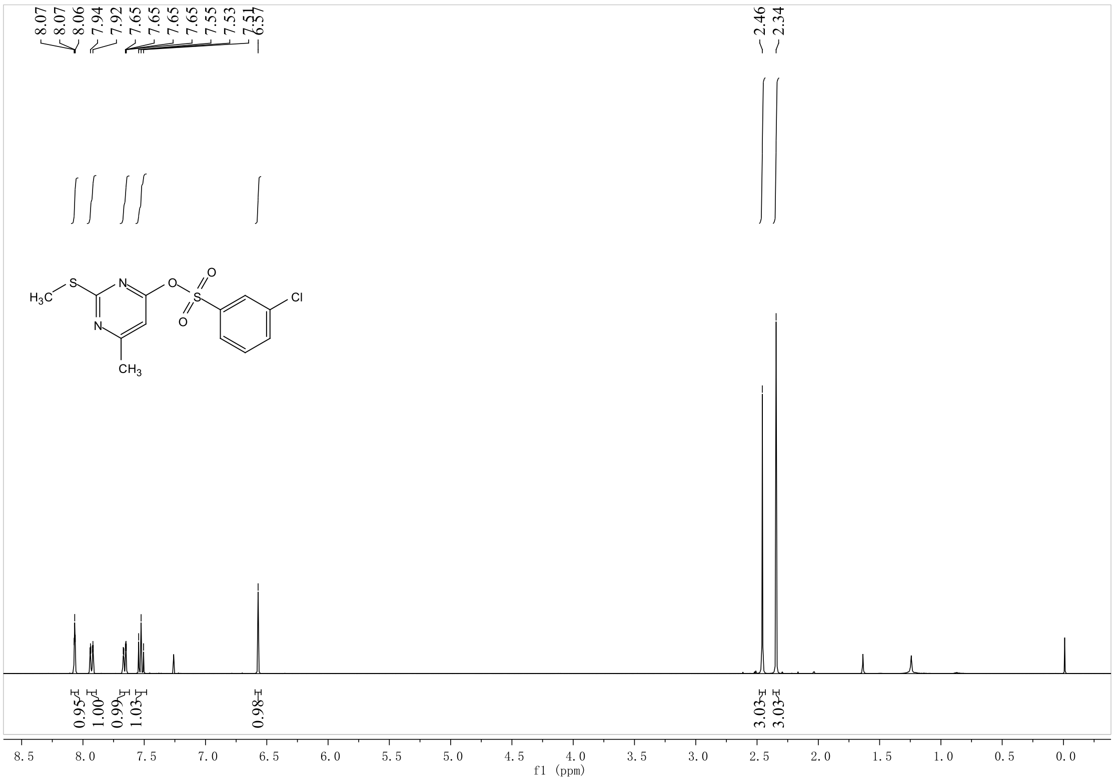

^{1}H NMR of compound A_7

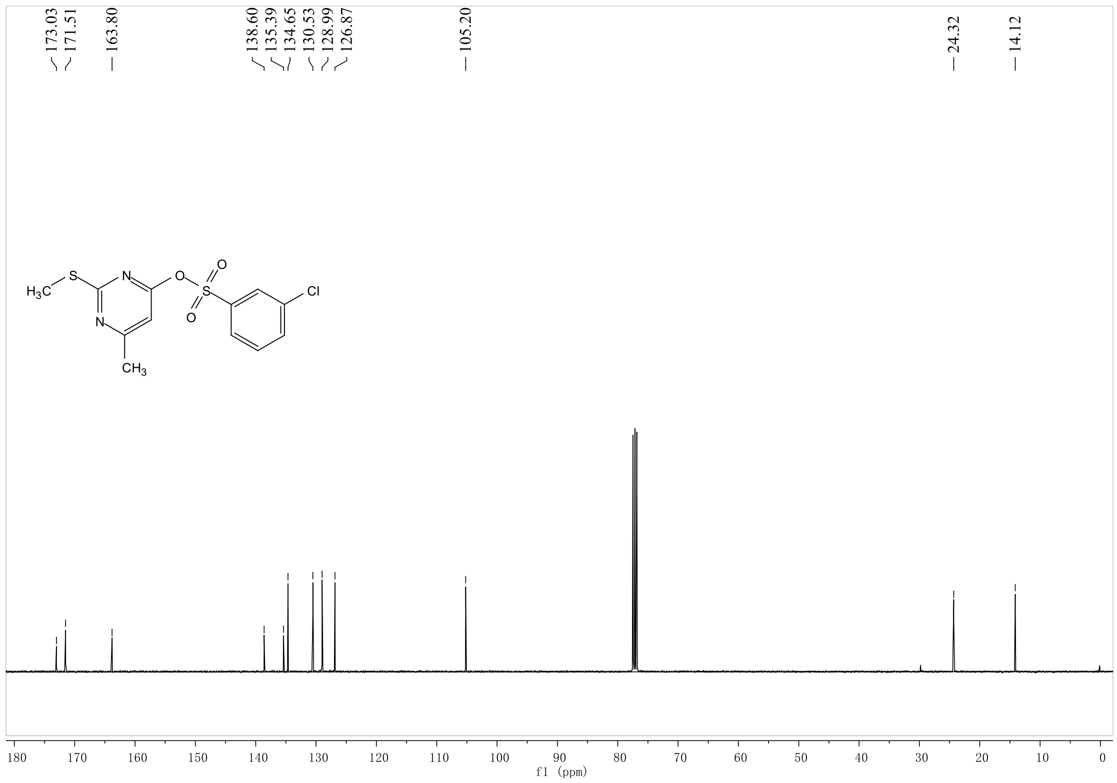

^{13}C NMR of compound A7

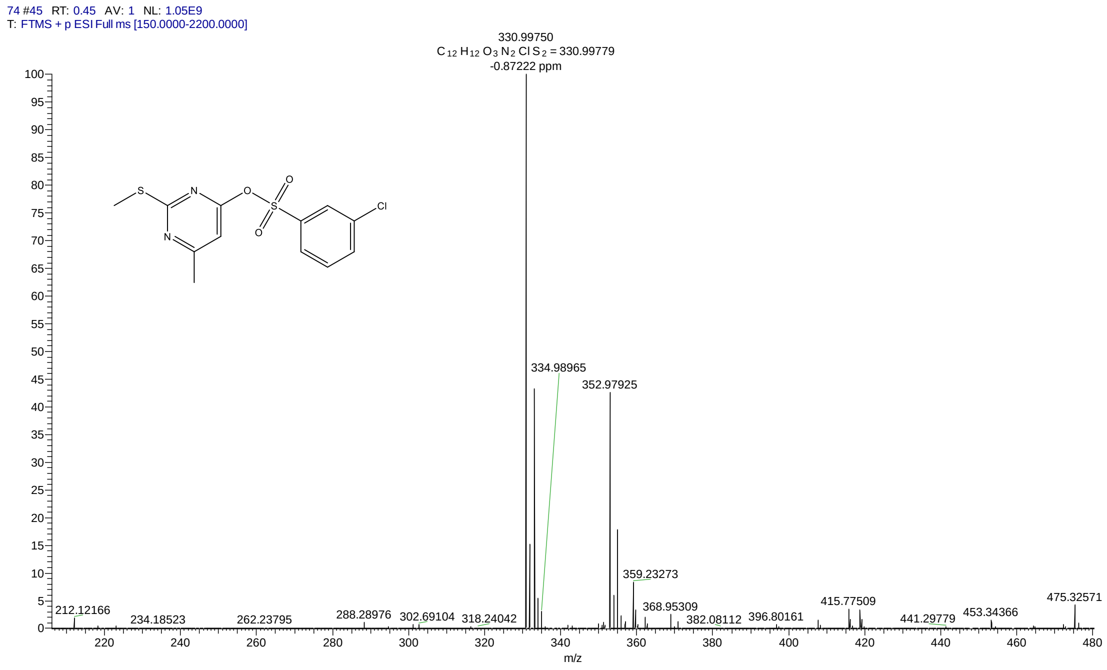

HRMS NMR of compound A7

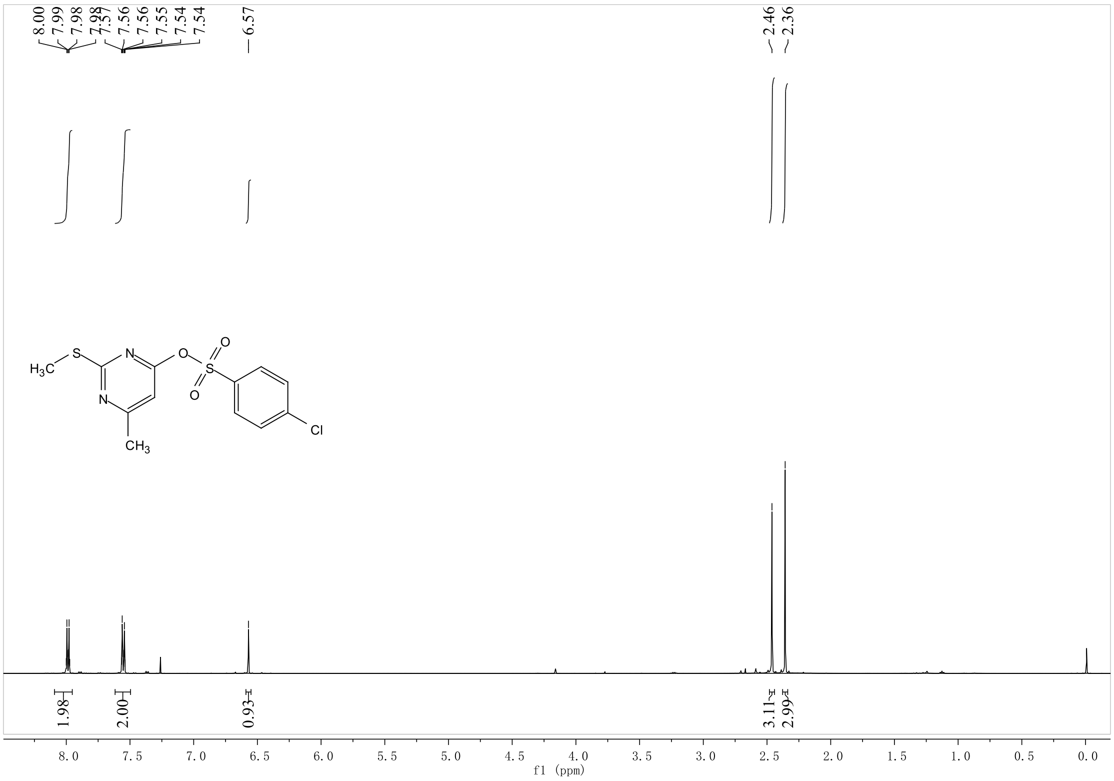

^{1}H NMR of compound A8

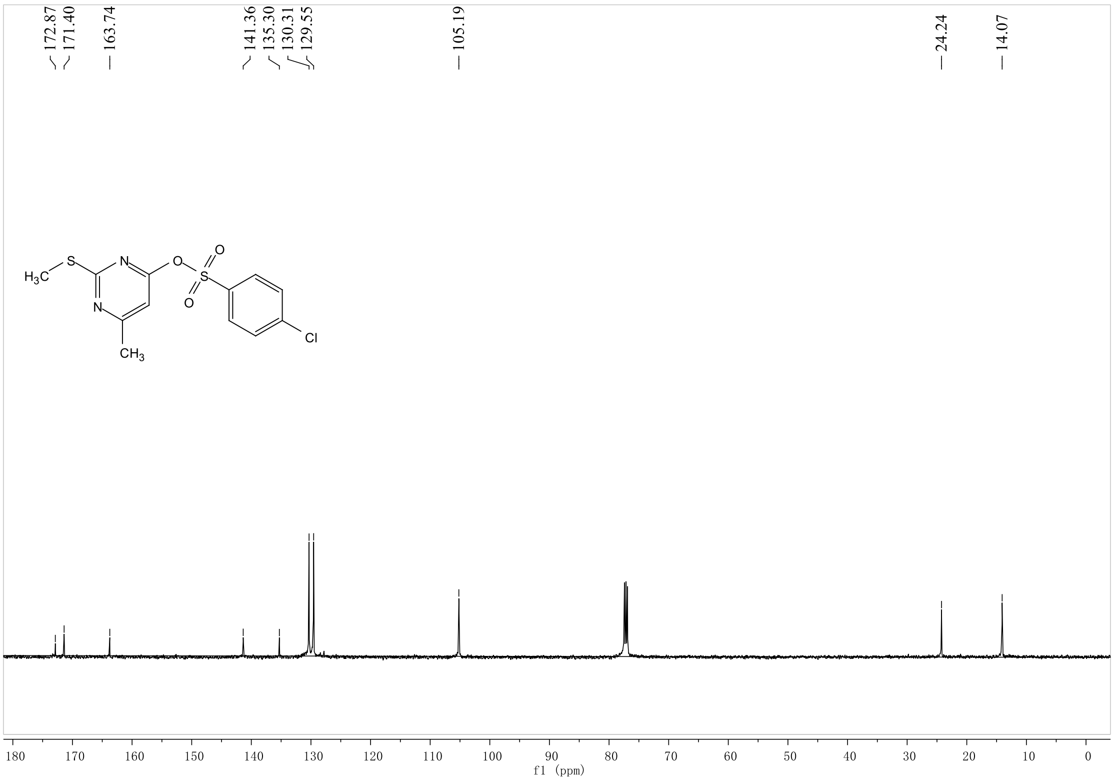

^{13}C NMR of compound A8

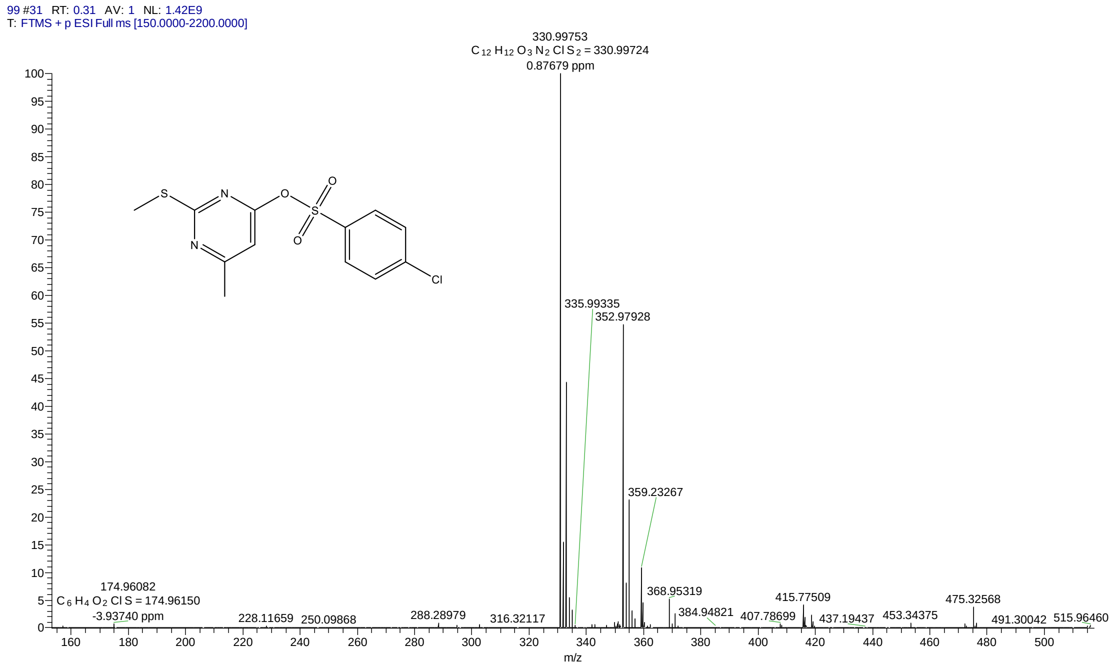

HRMS NMR of compound A8

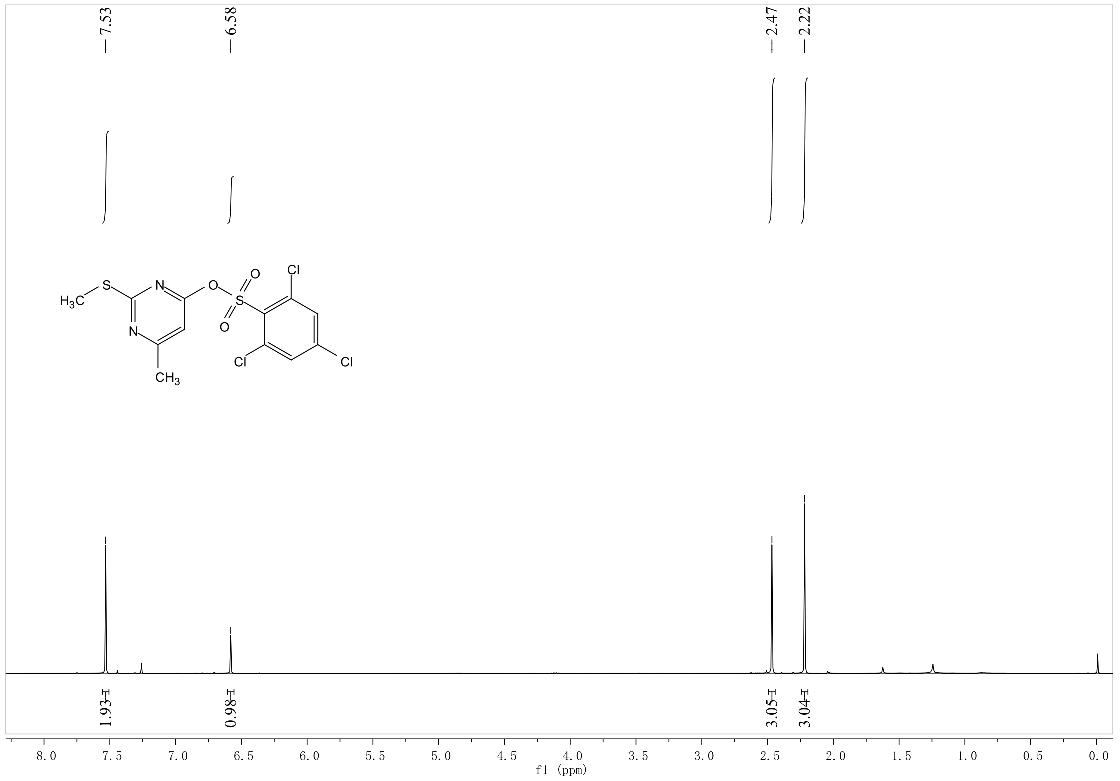

^{1}H NMR of compound A9

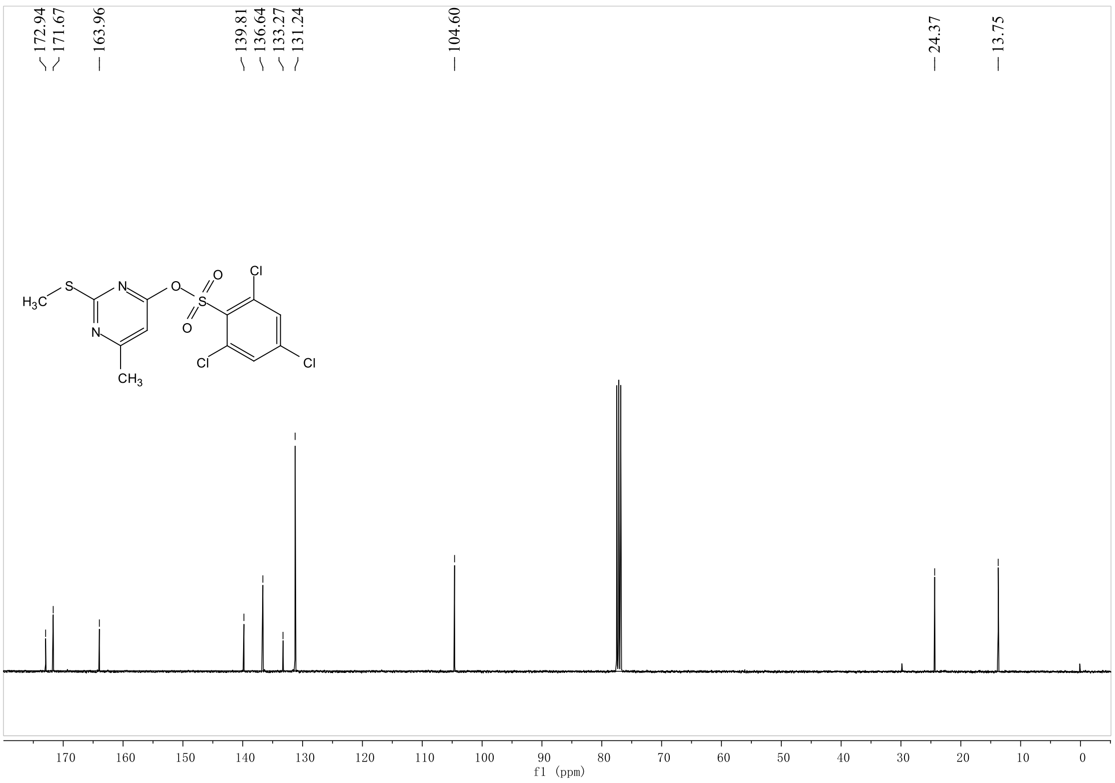

^{13}C NMR of compound A9

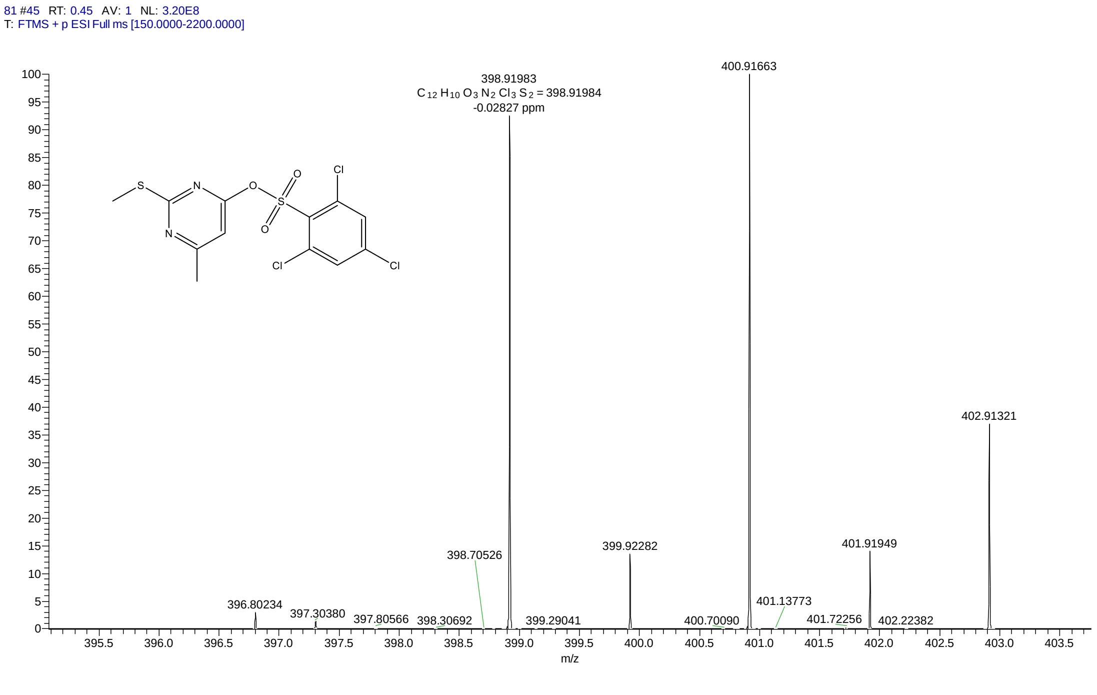

HRMS NMR of compound A9

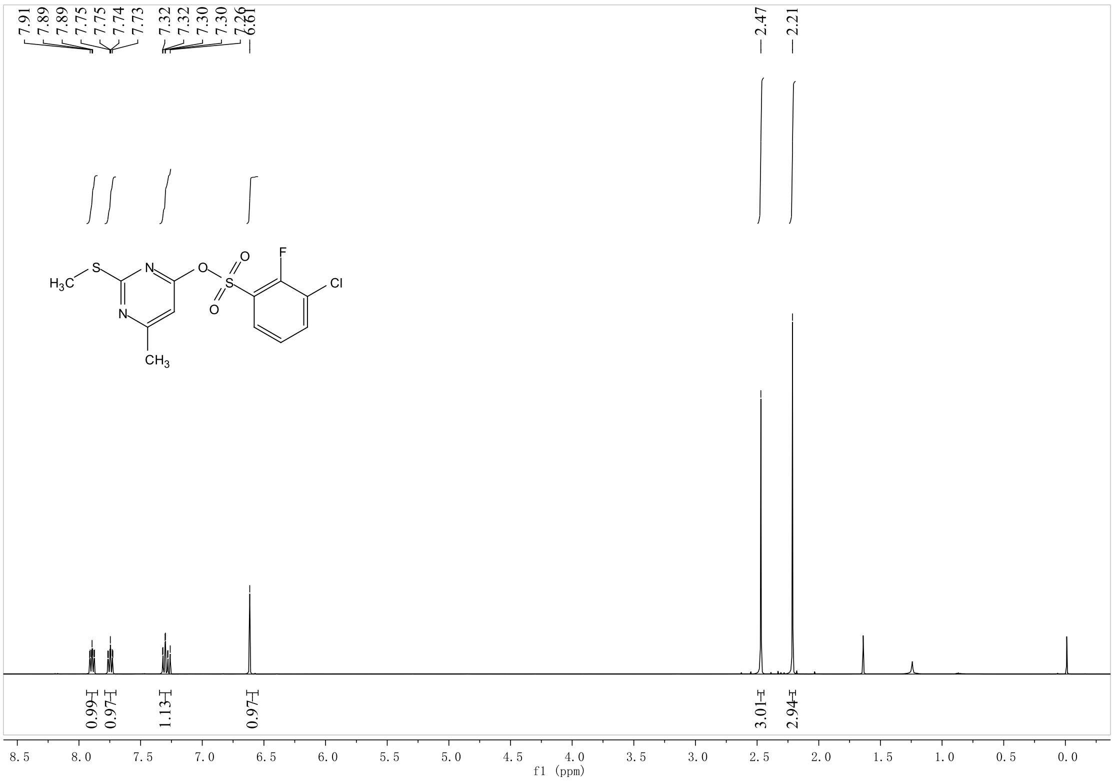

^{1}H NMR of compound A10

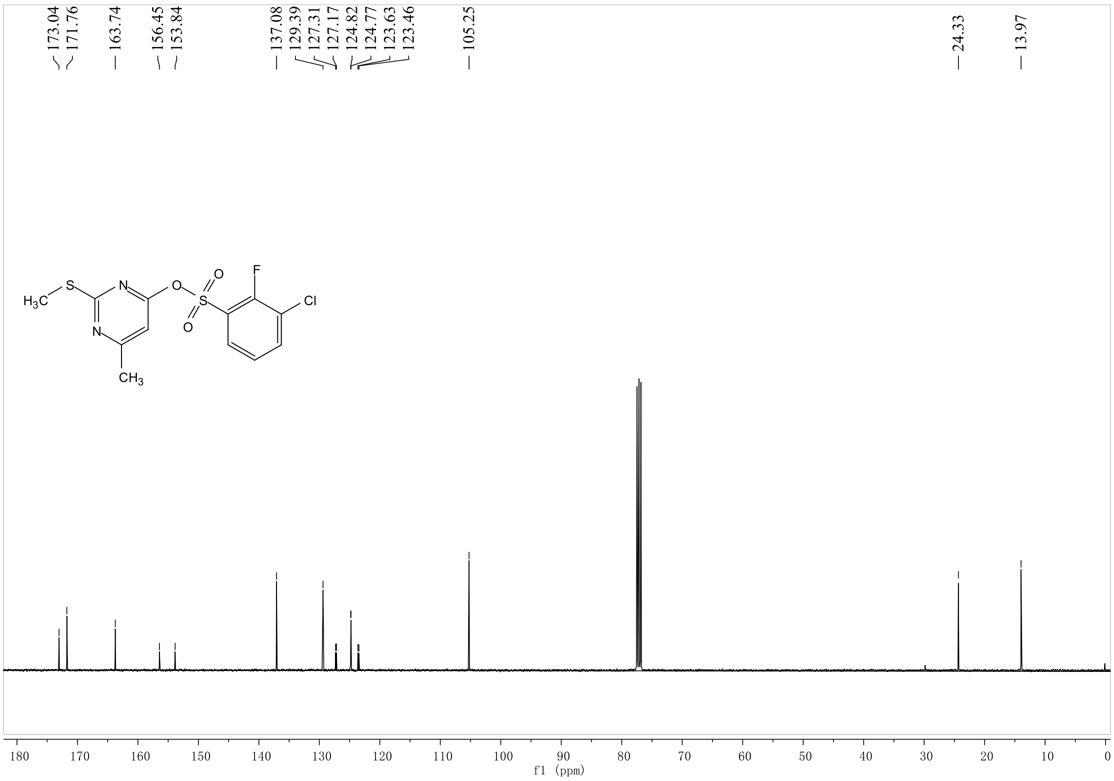

^{13}C NMR of compound A10

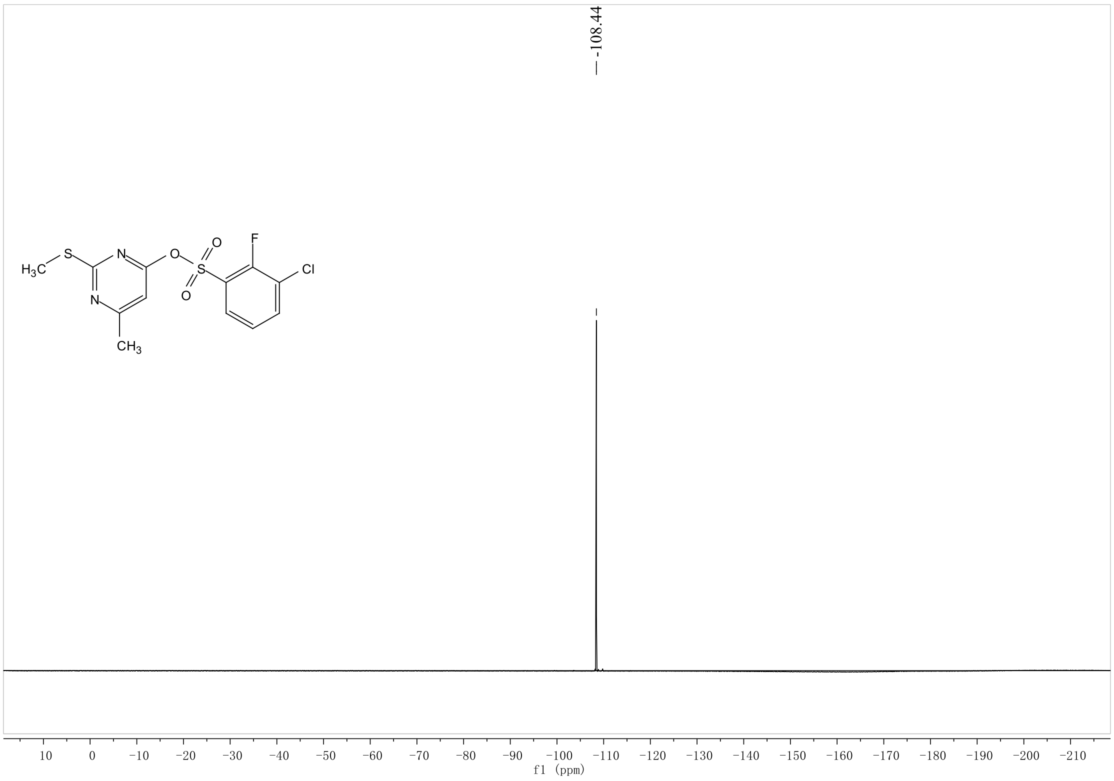

^{19}F NMR of compound A10

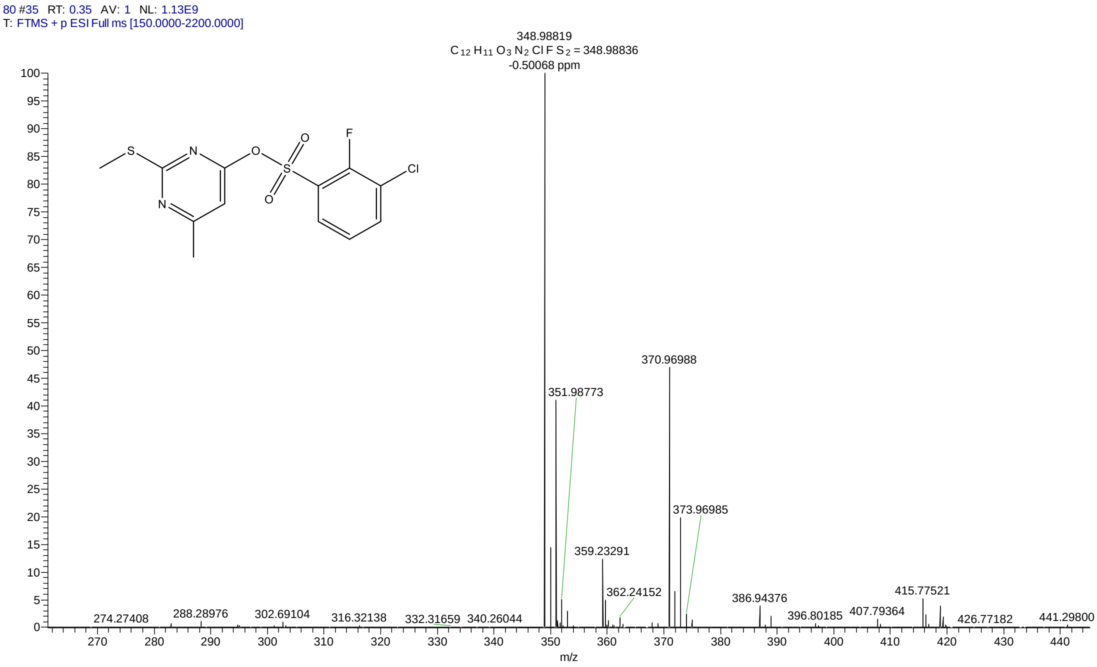

HRMS NMR of compound A10

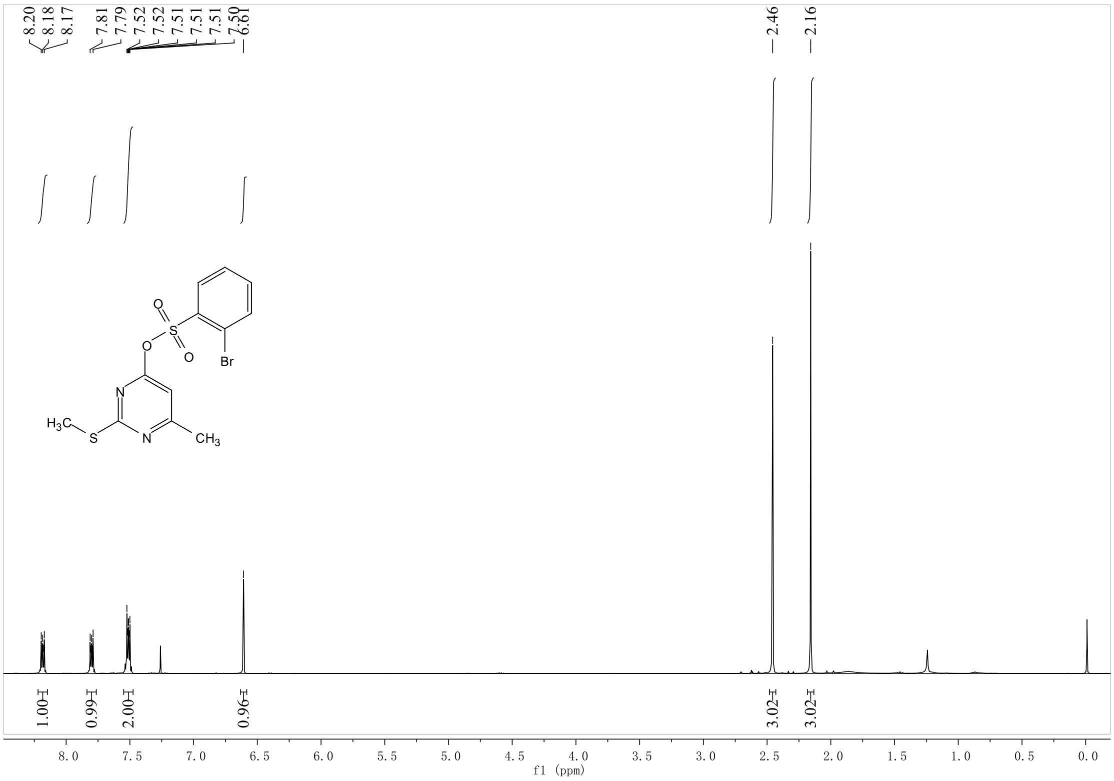

^{1}H NMR of compound A11

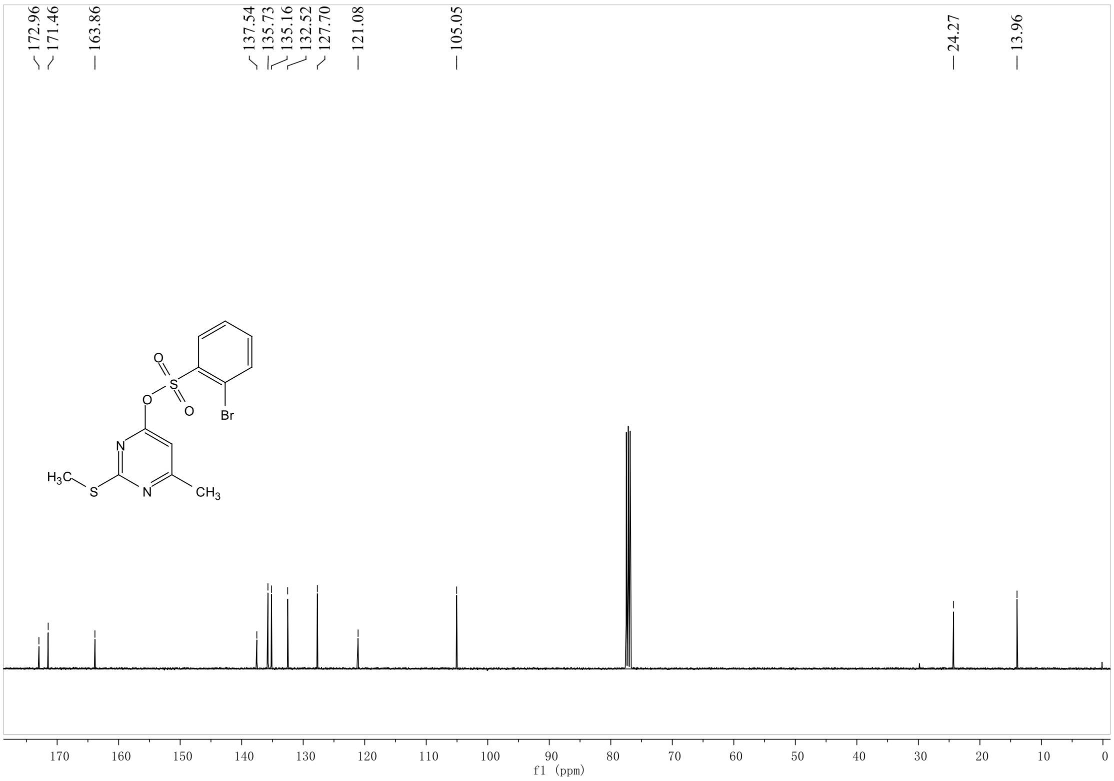

^{13}C NMR of compound A11

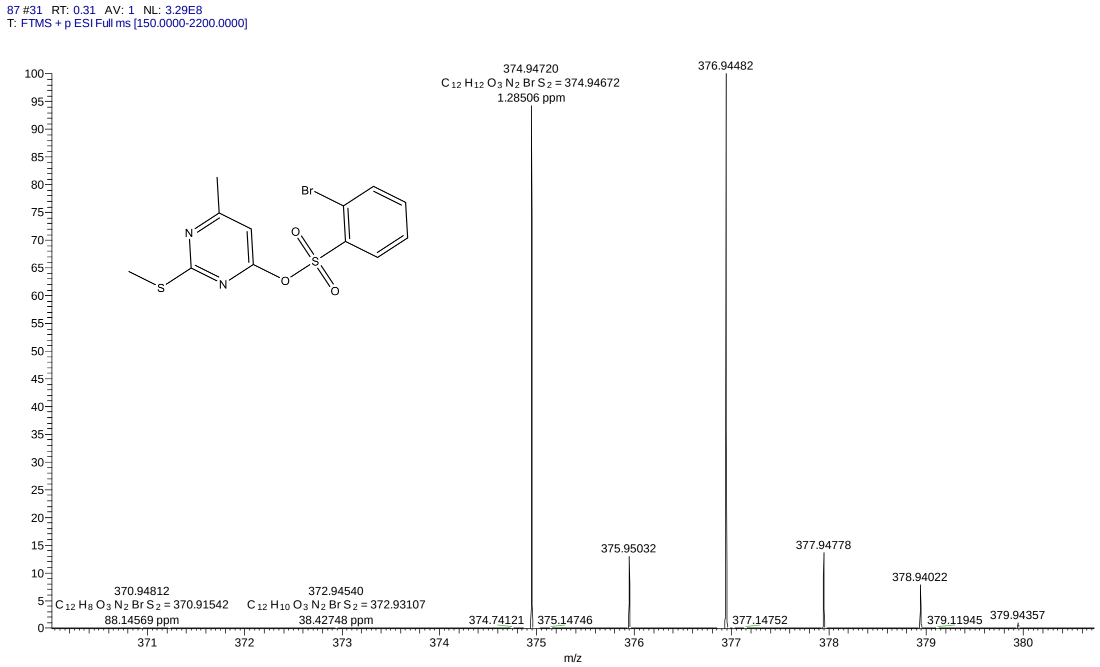

HRMS NMR of compound A11

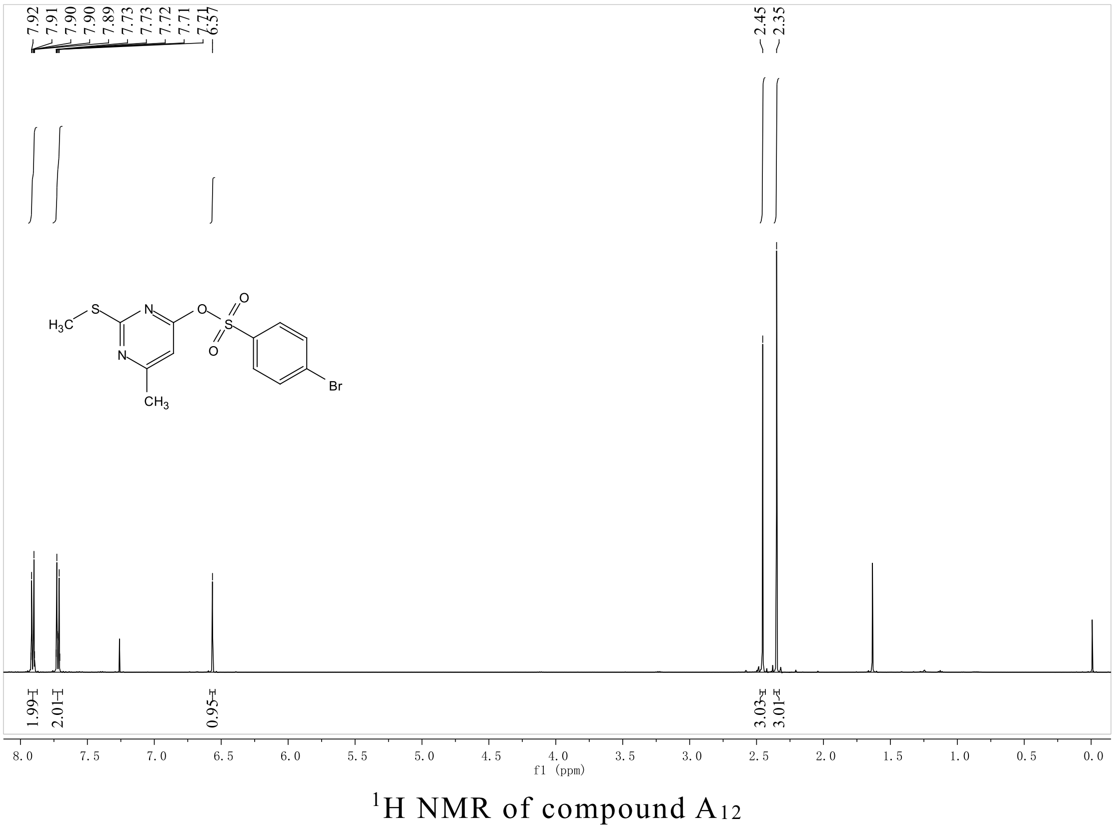

^{1}H NMR of compound A12

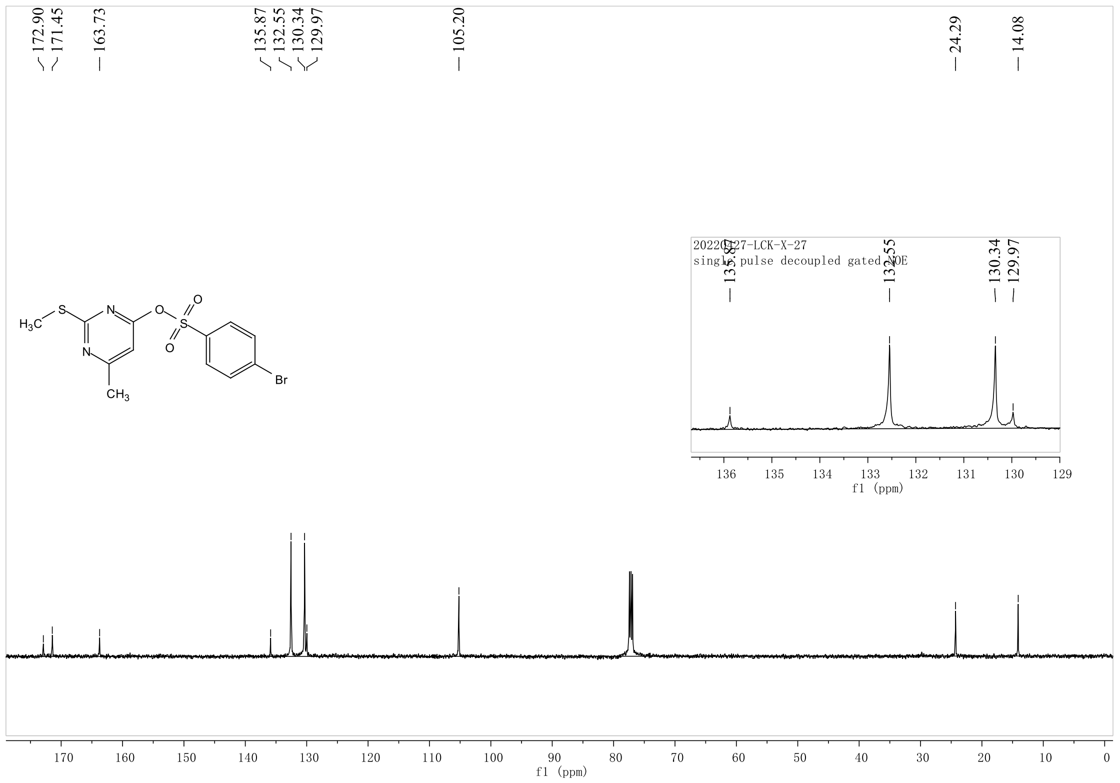

^{13}C NMR of compound A12

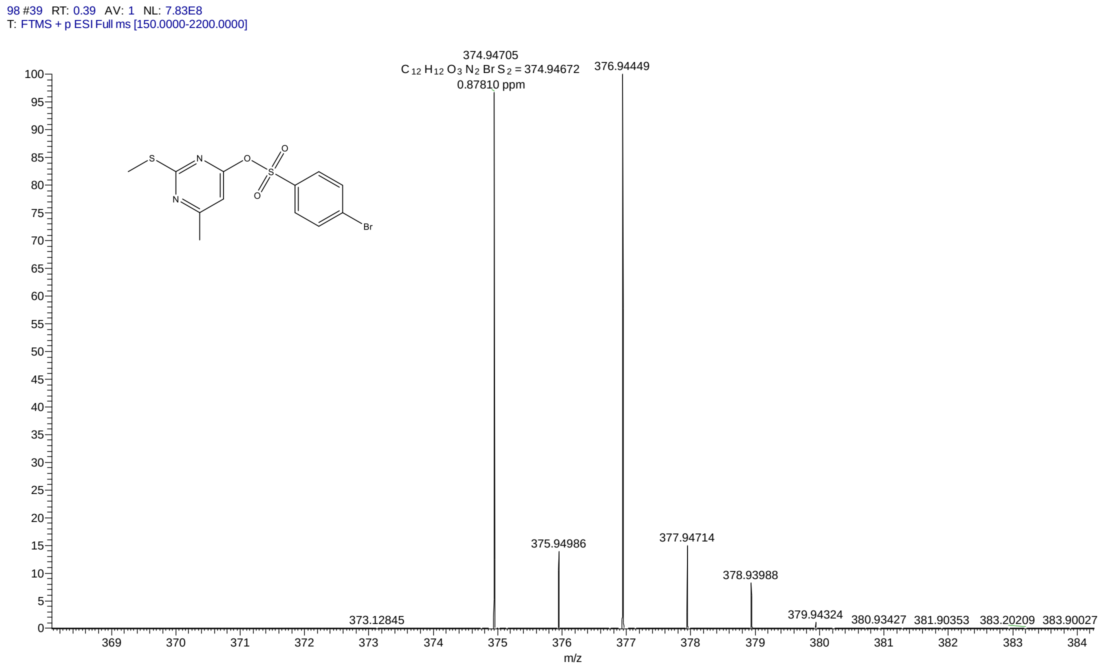

HRMS of compound A12

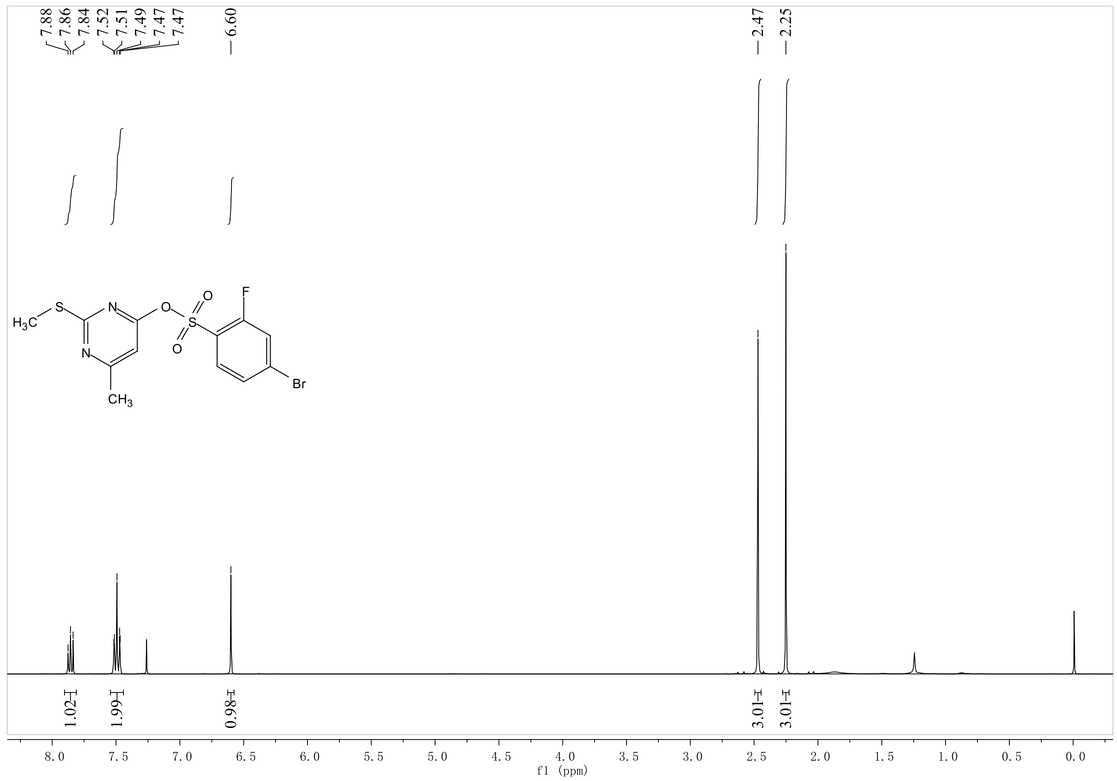

^{1}H NMR of compound A13

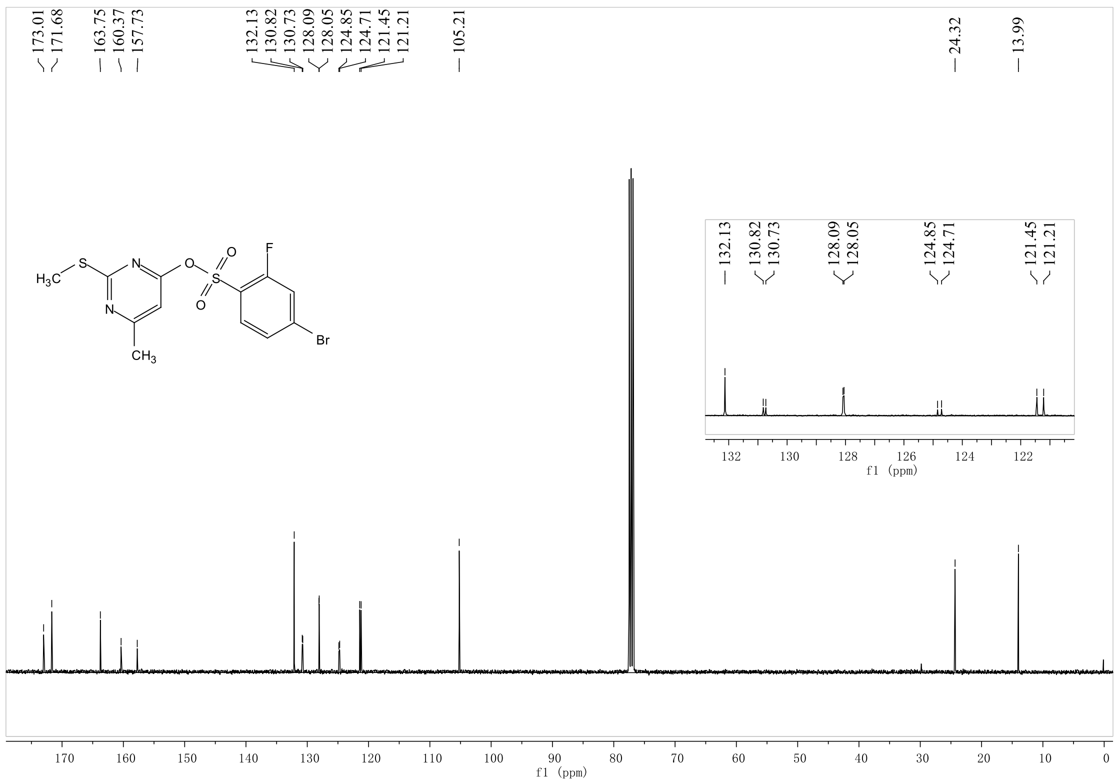

^{13}C NMR of compound A13

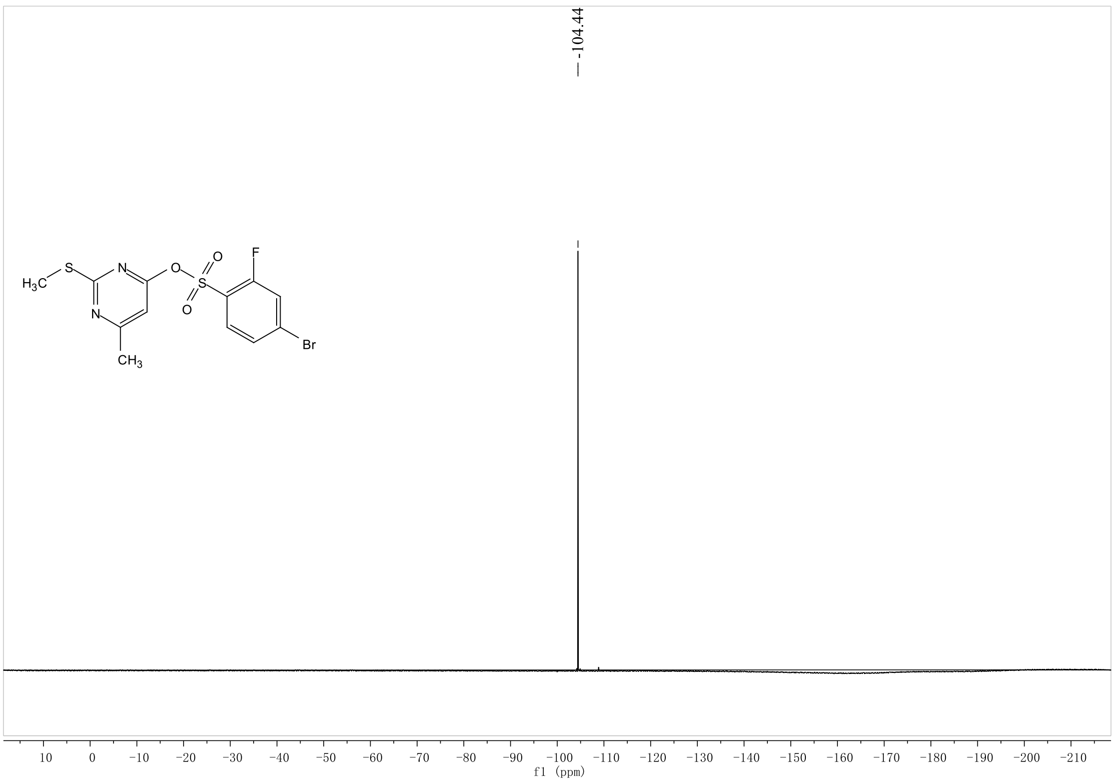

^{19}F NMR of compound A13

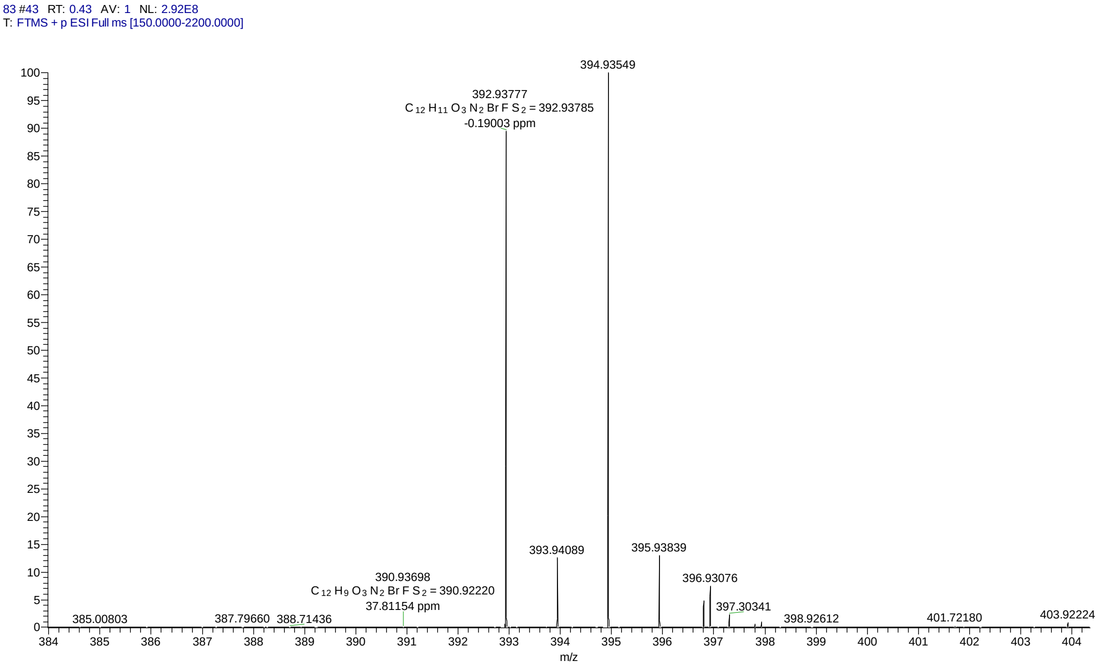

HRMS of compound A13

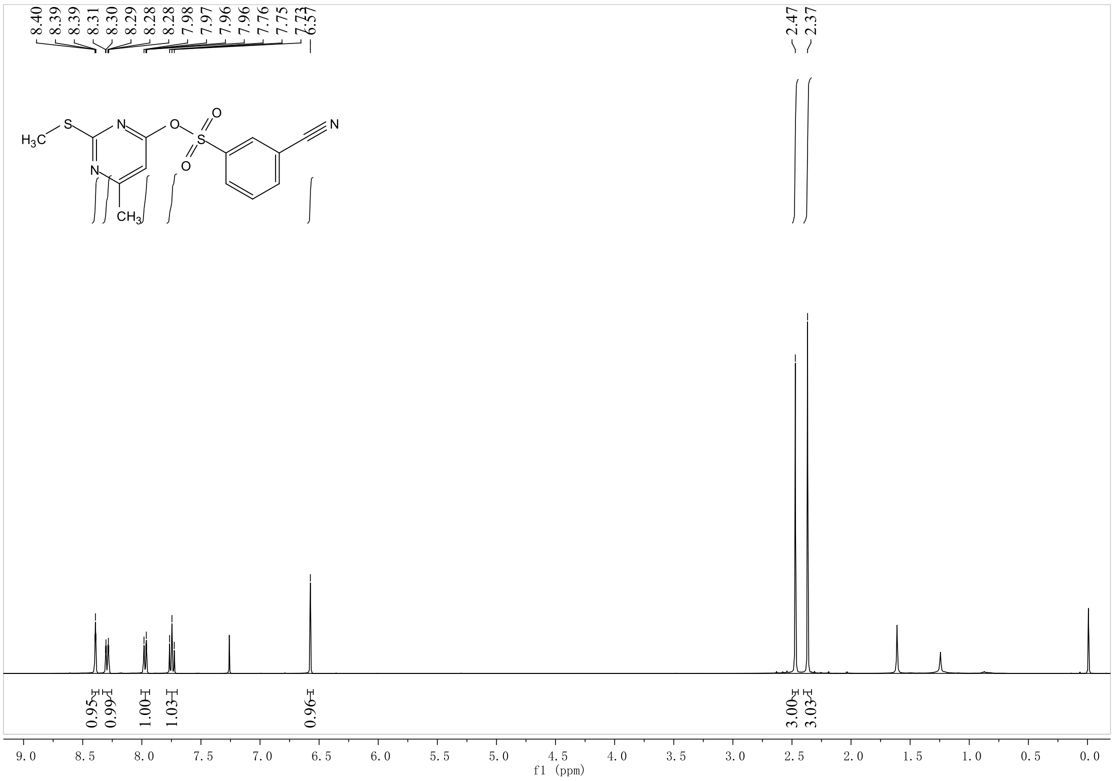

^{1}H NMR of compound A14

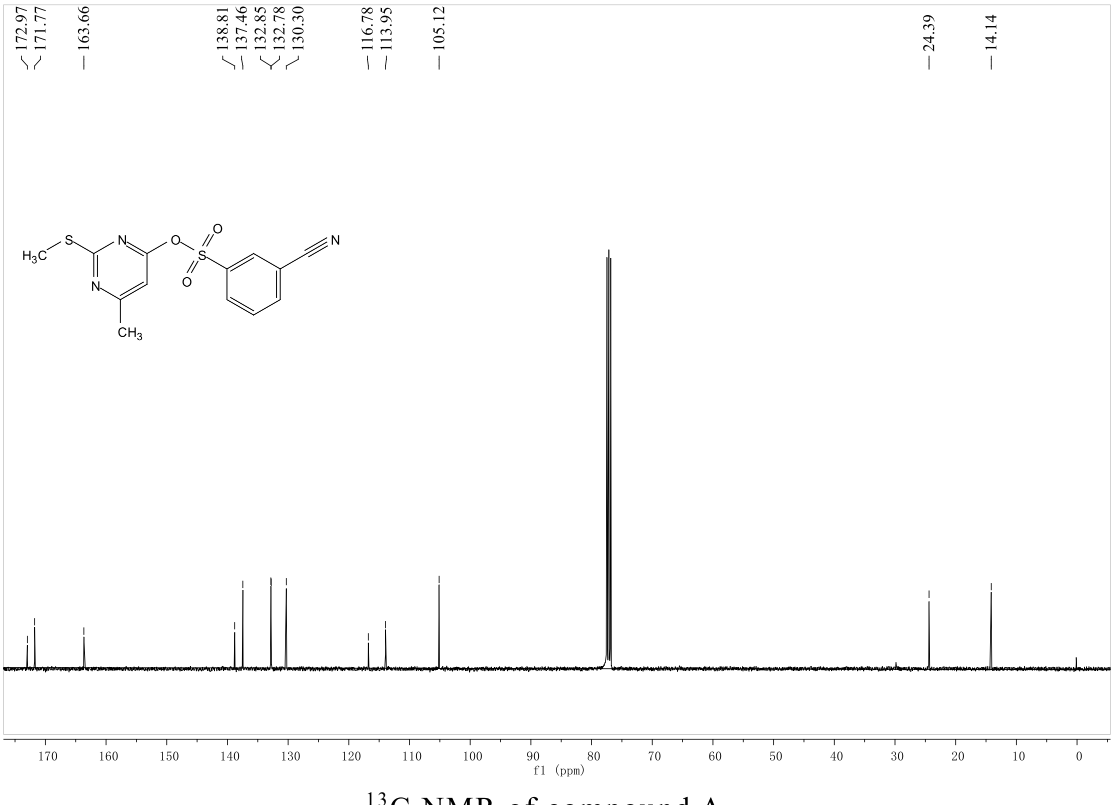

^{13}C NMR of compound A14

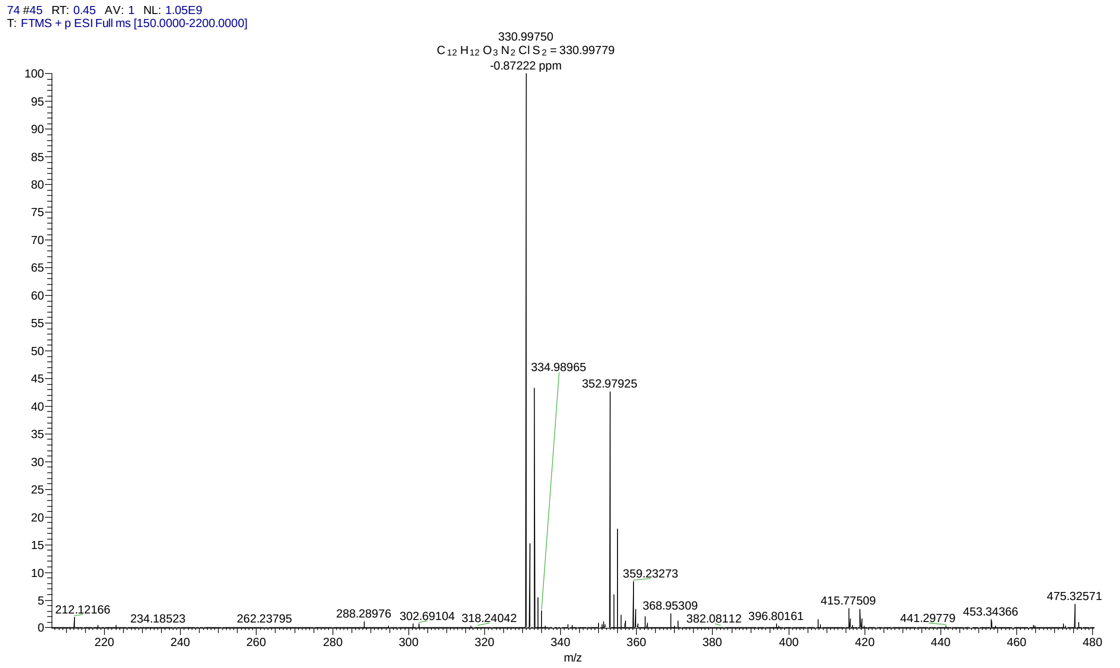

HRMS NMR of compound A14

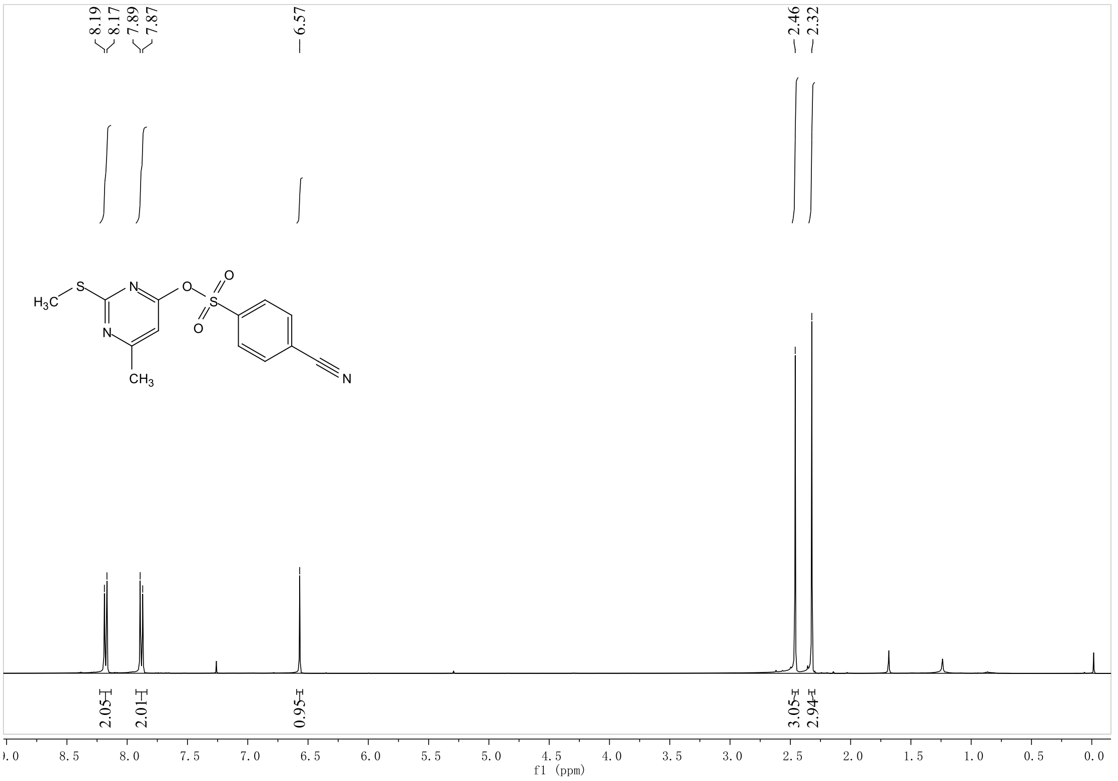

^{1}H NMR of compound A15

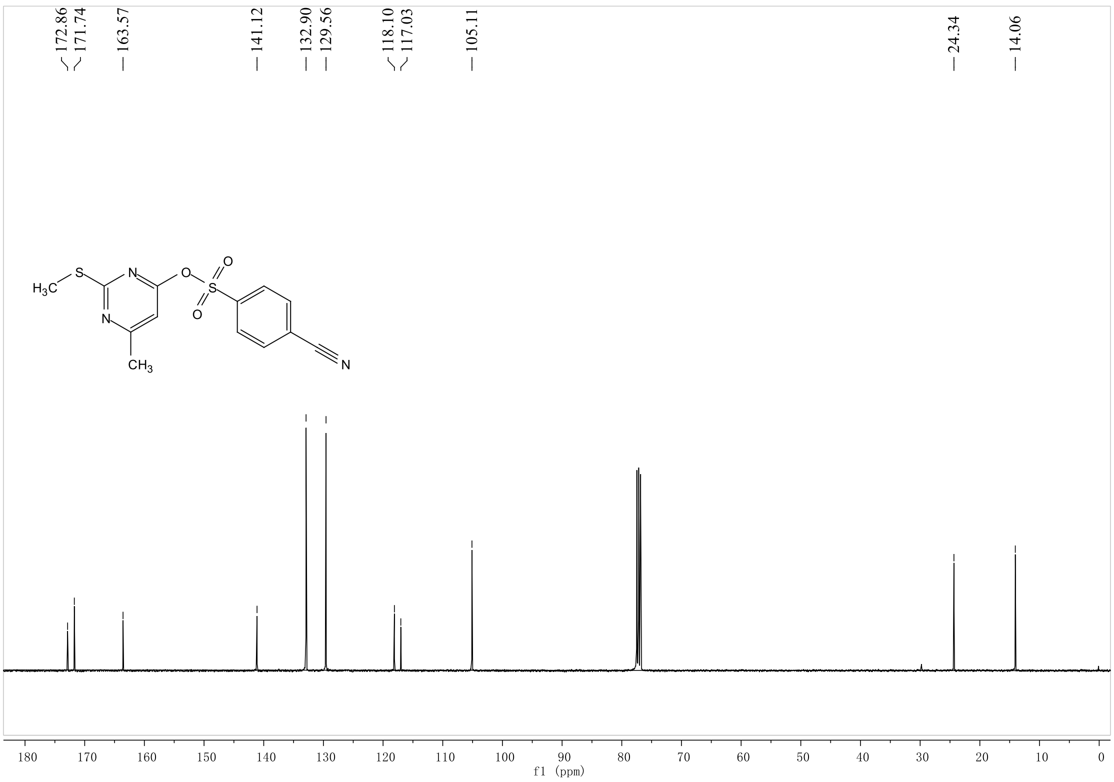

^{13}C NMR of compound A15

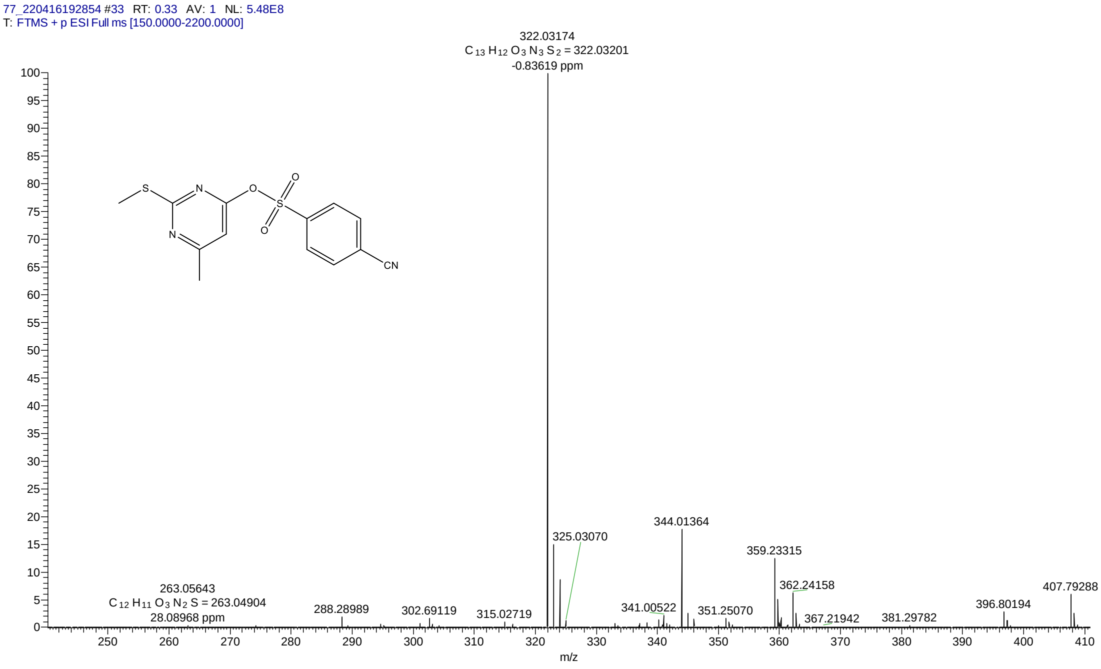

HRMS of compound A15

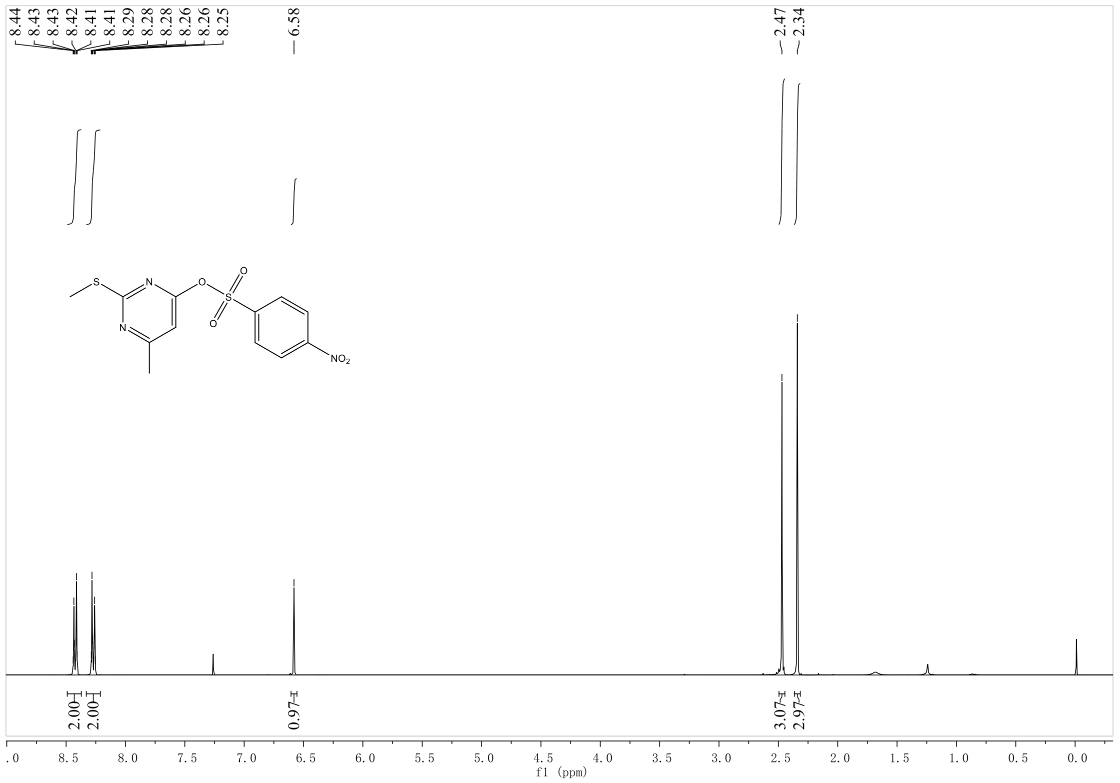

^{1}H NMR of compound A16

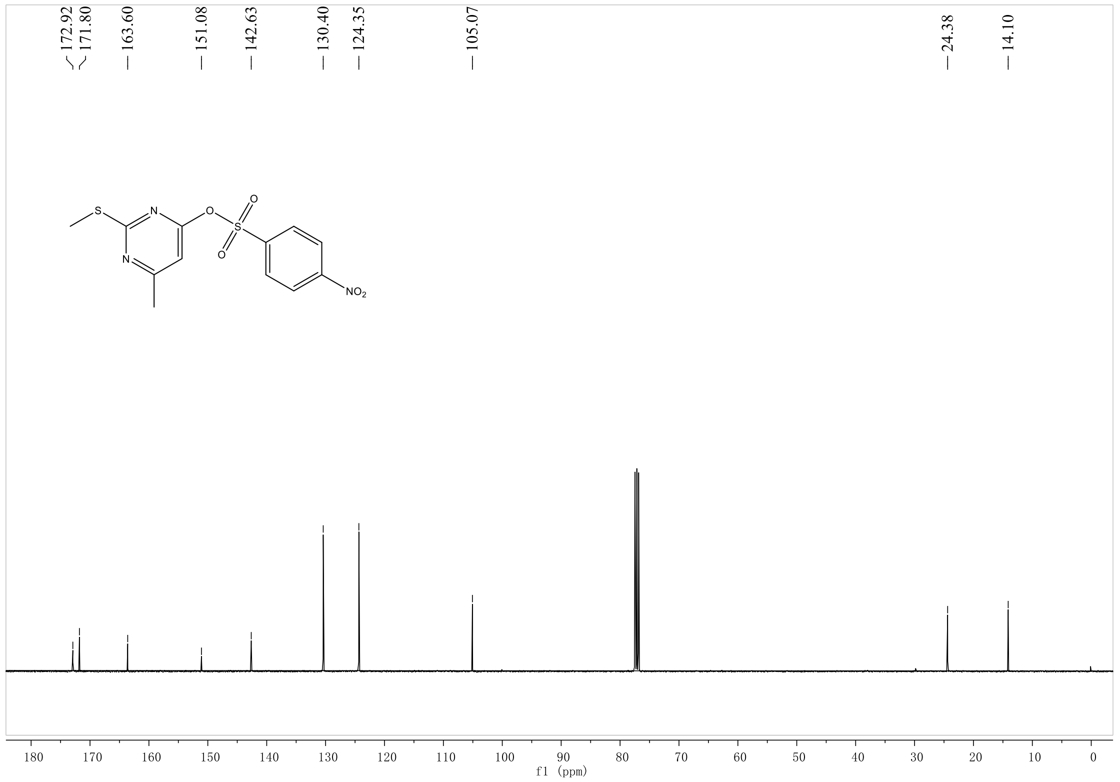

^{13}C NMR of compound A16

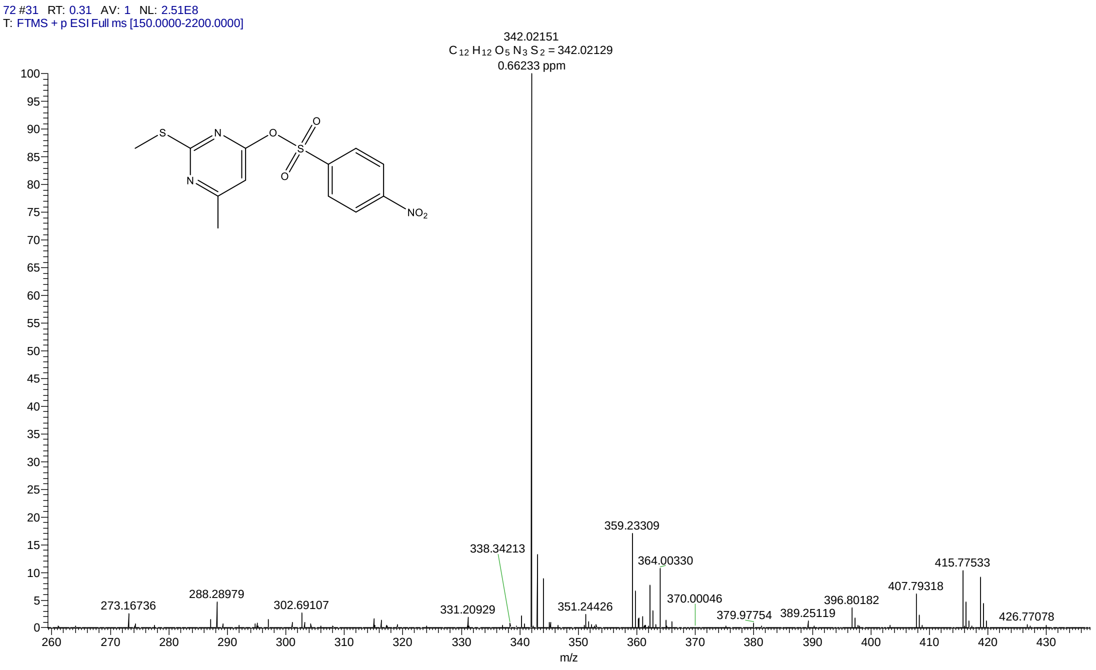

HRMS of compound A16

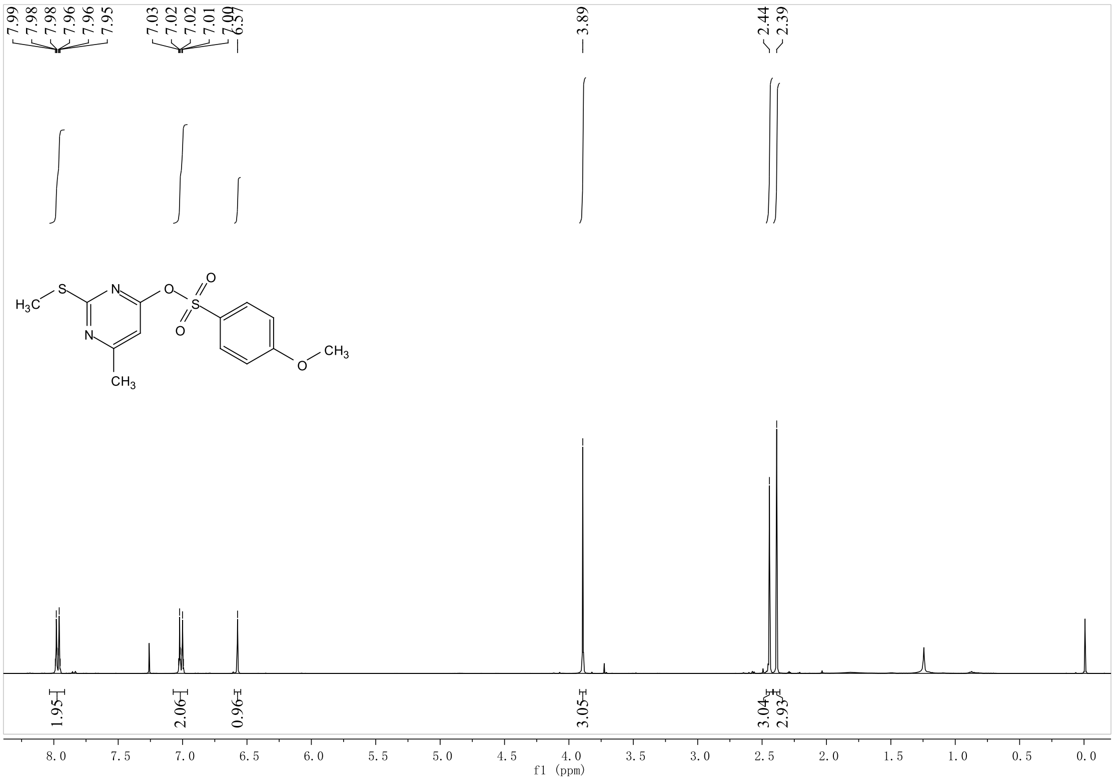

^{1}H NMR of compound A17

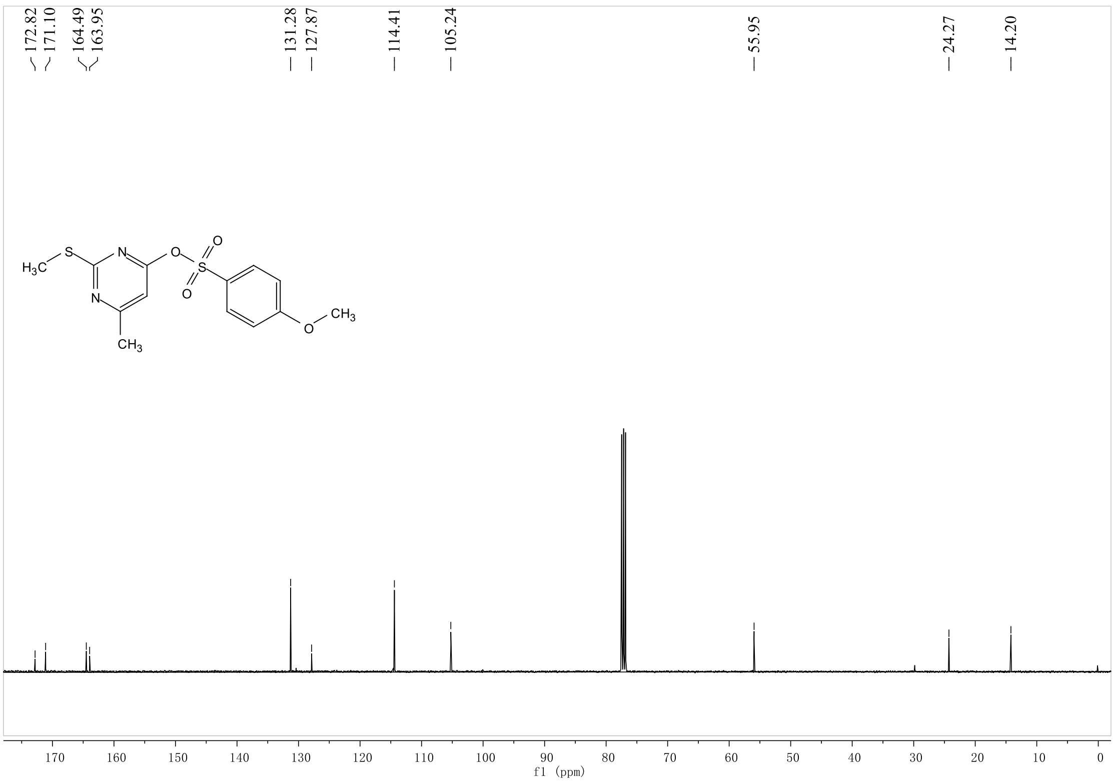

^{13}C NMR of compound A17

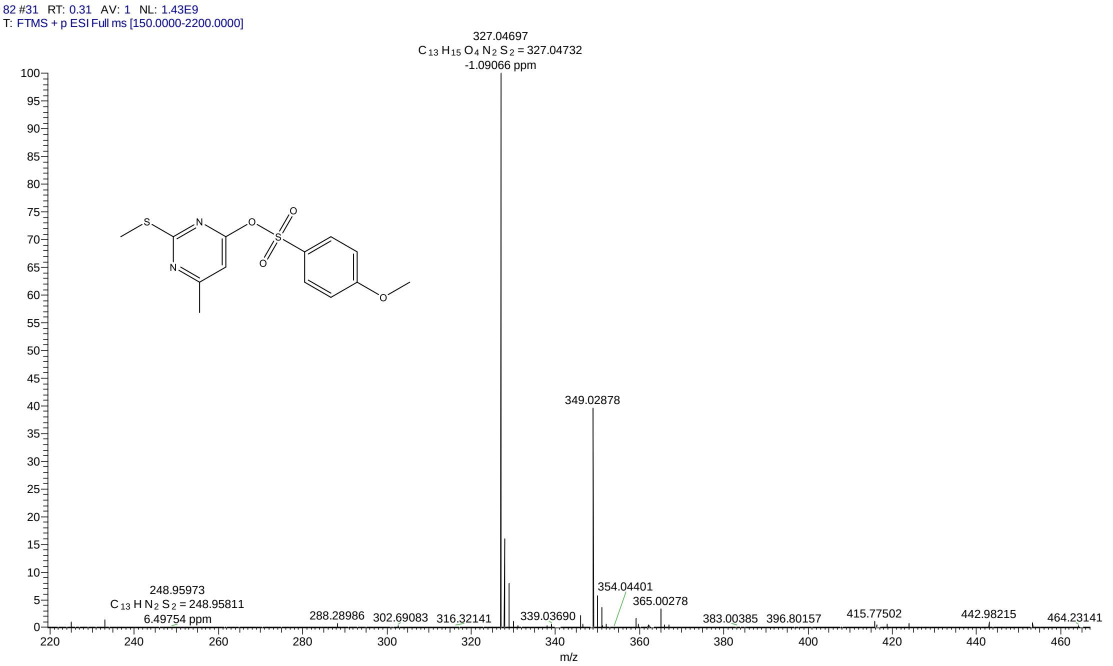

HRMS of compound A17

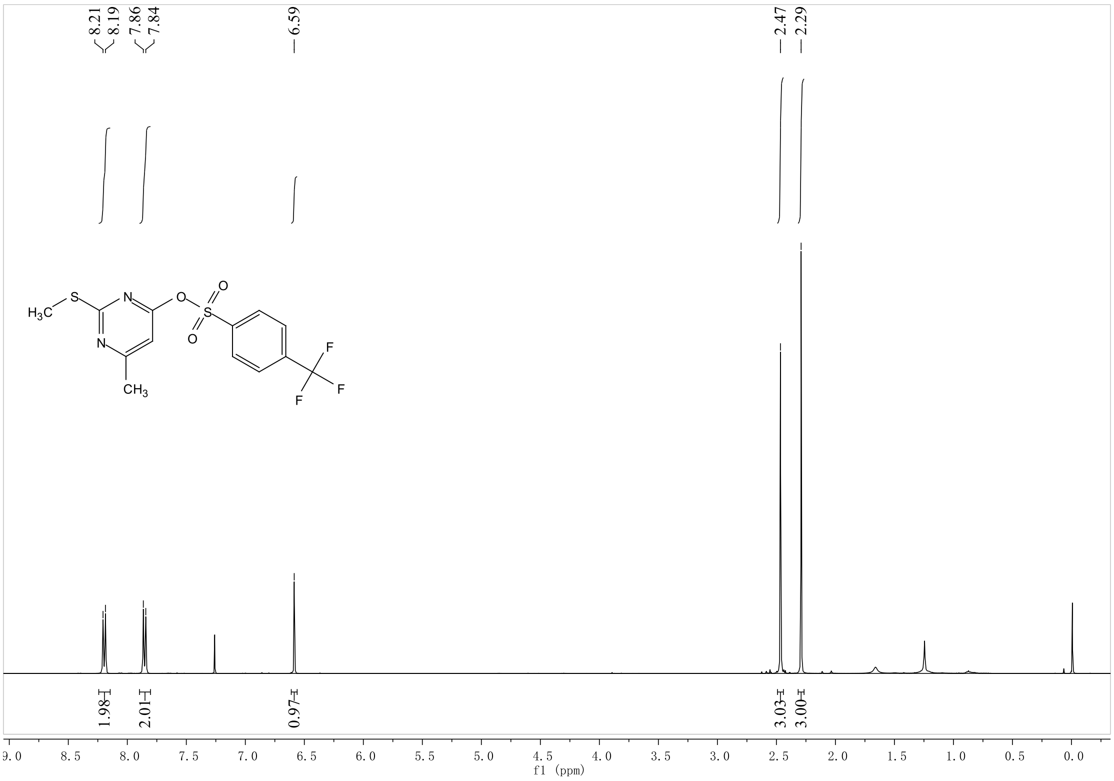

^{1}H NMR of compound A18

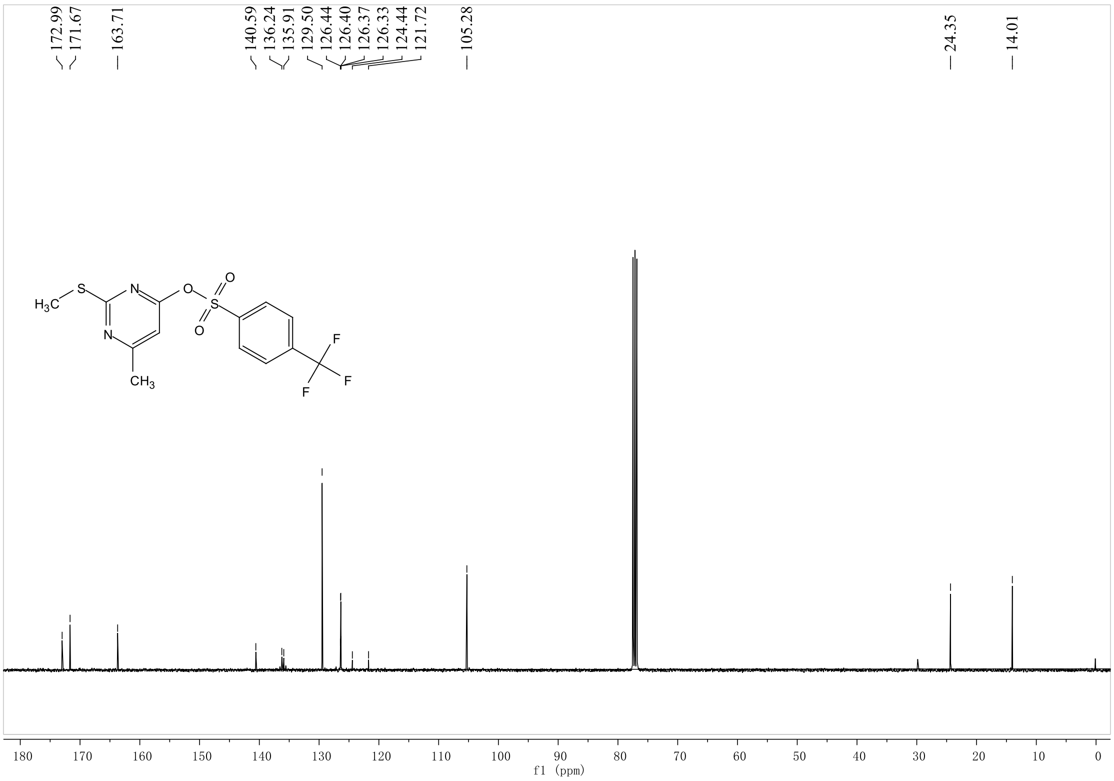

^{13}C NMR of compound A18

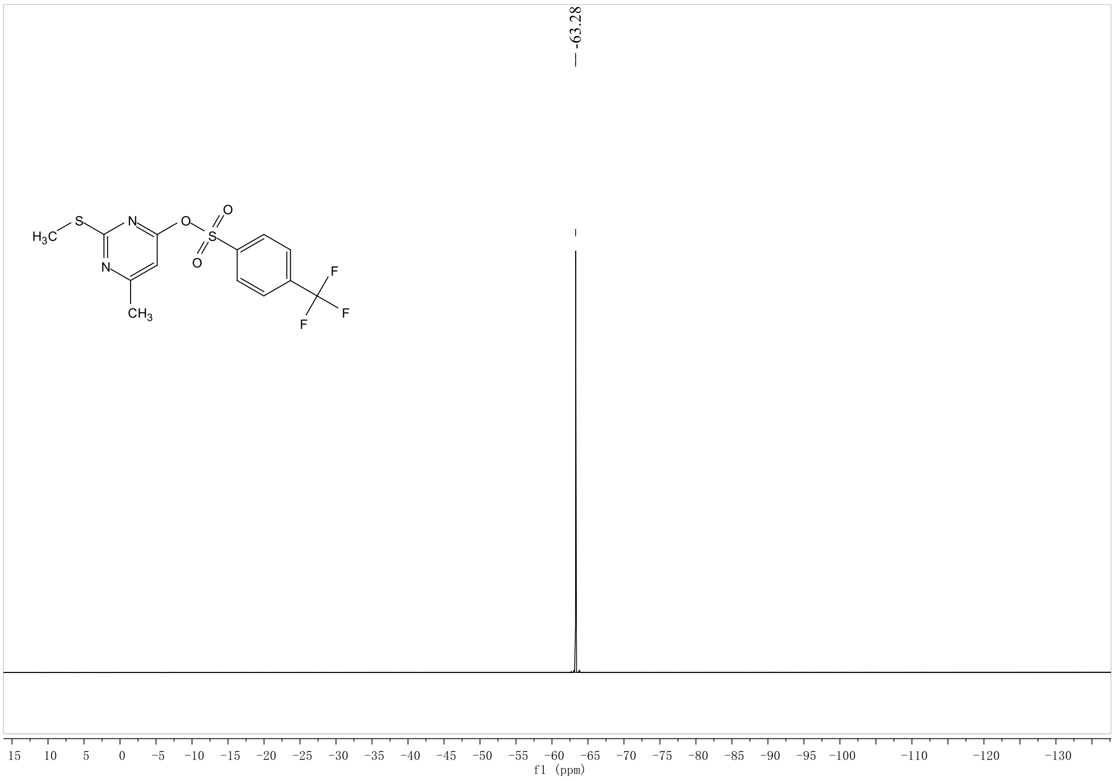

^{19}F NMR of compound A_{18}

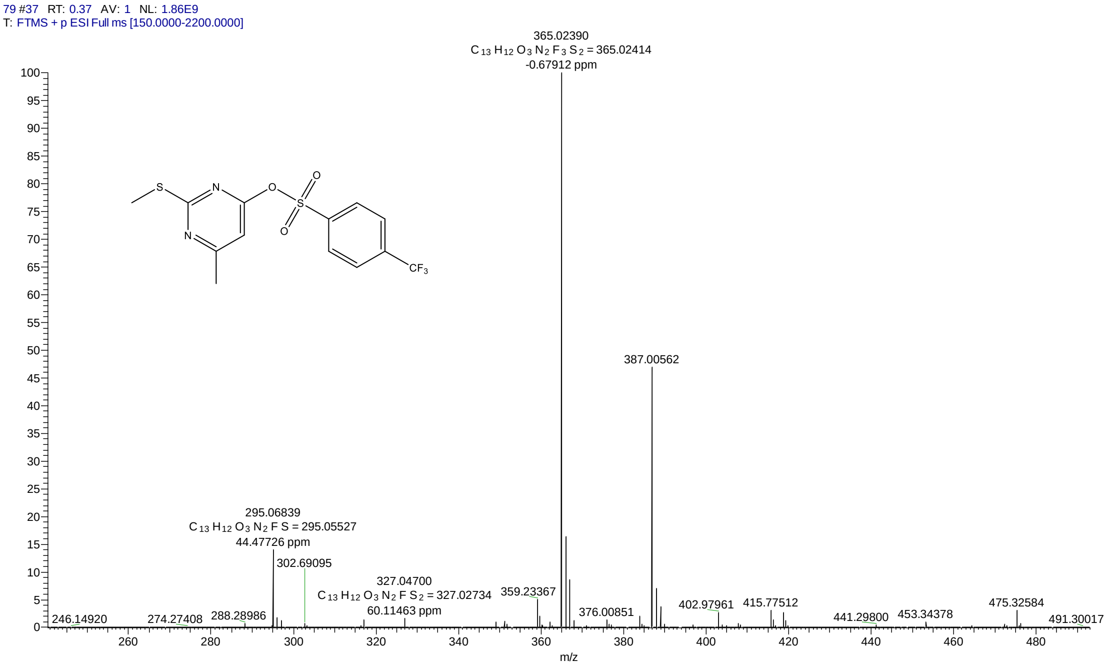

HRMS of compound A_{18}

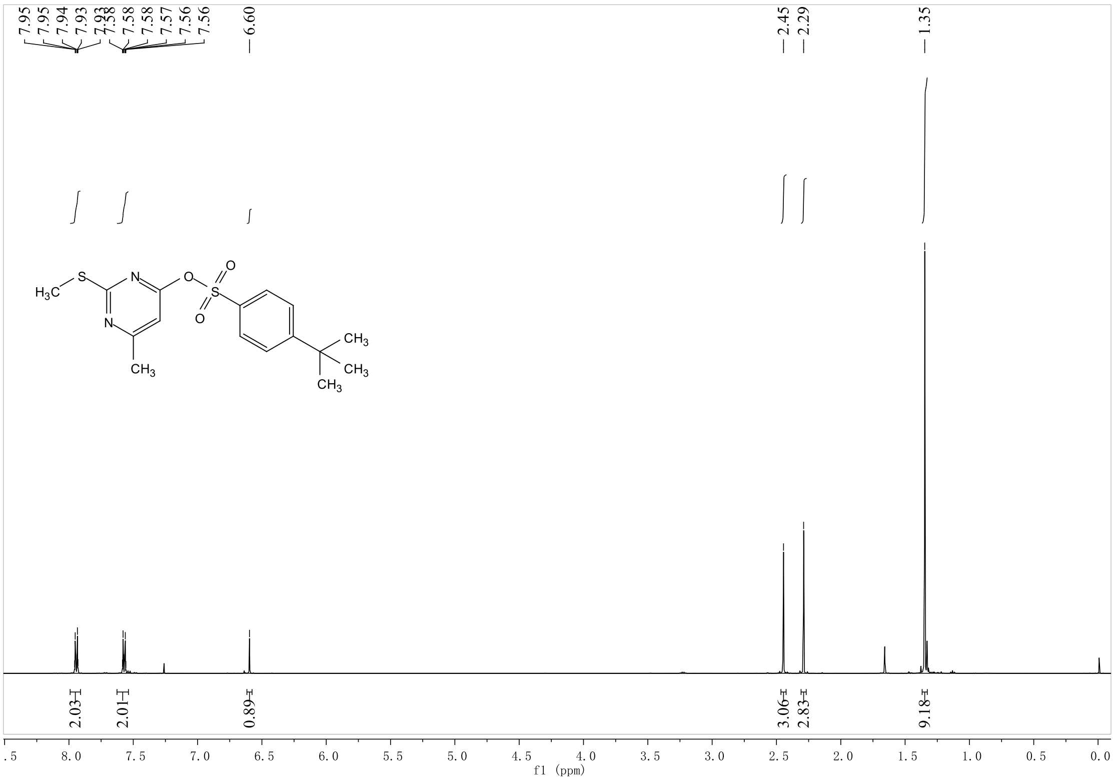

^{1}H NMR of compound A19

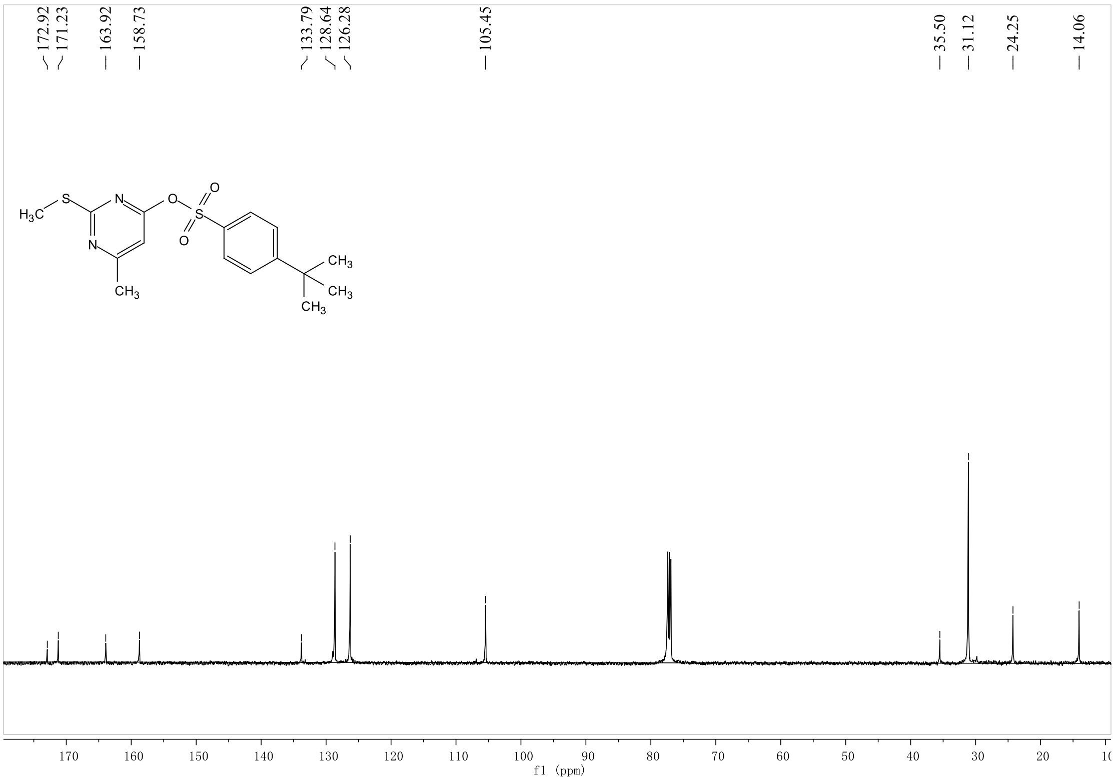

^{13}C NMR of compound A19

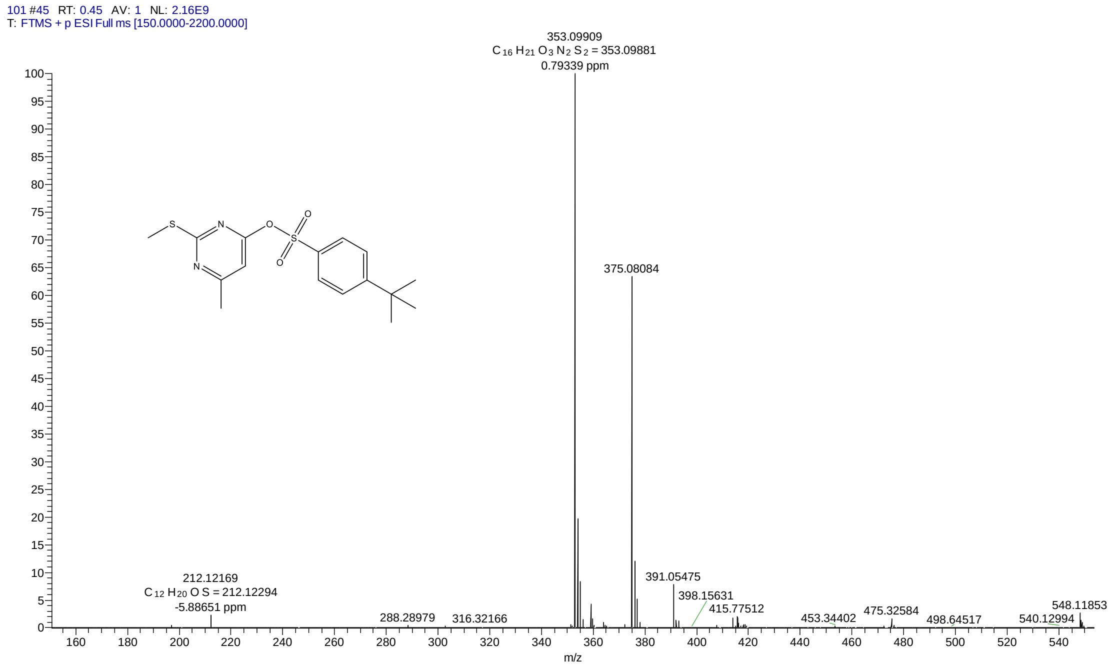

HRMS of compound A19

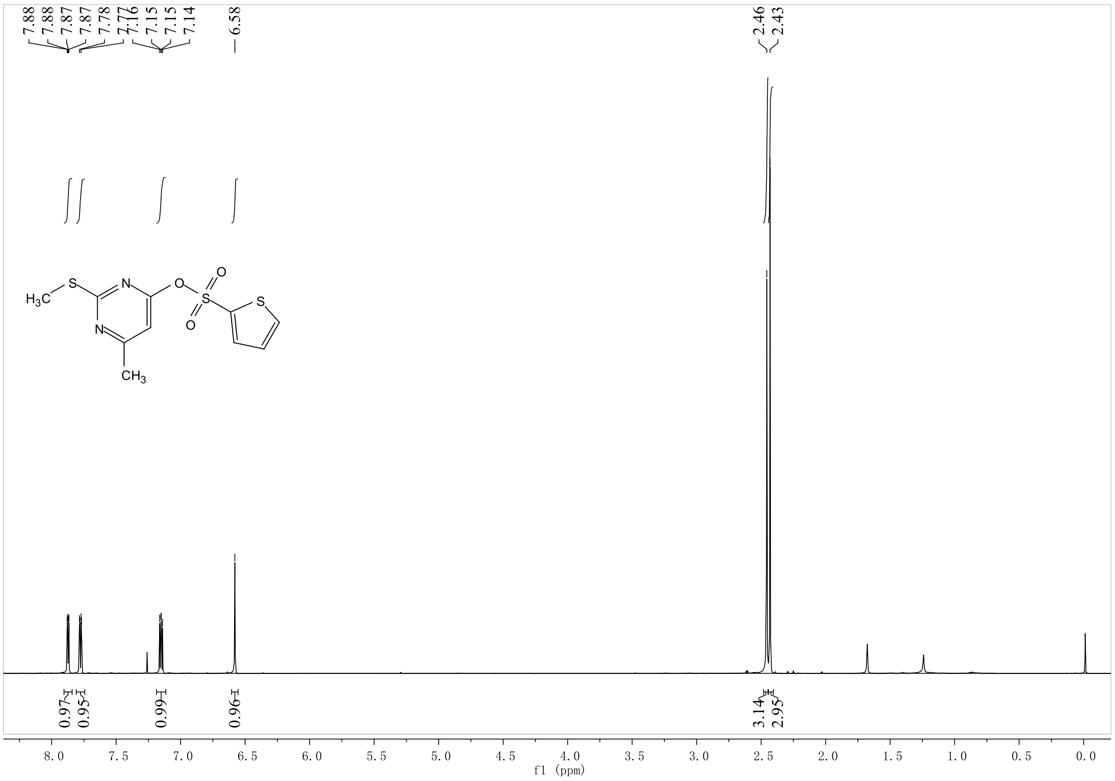

^{1}H NMR of compound A20

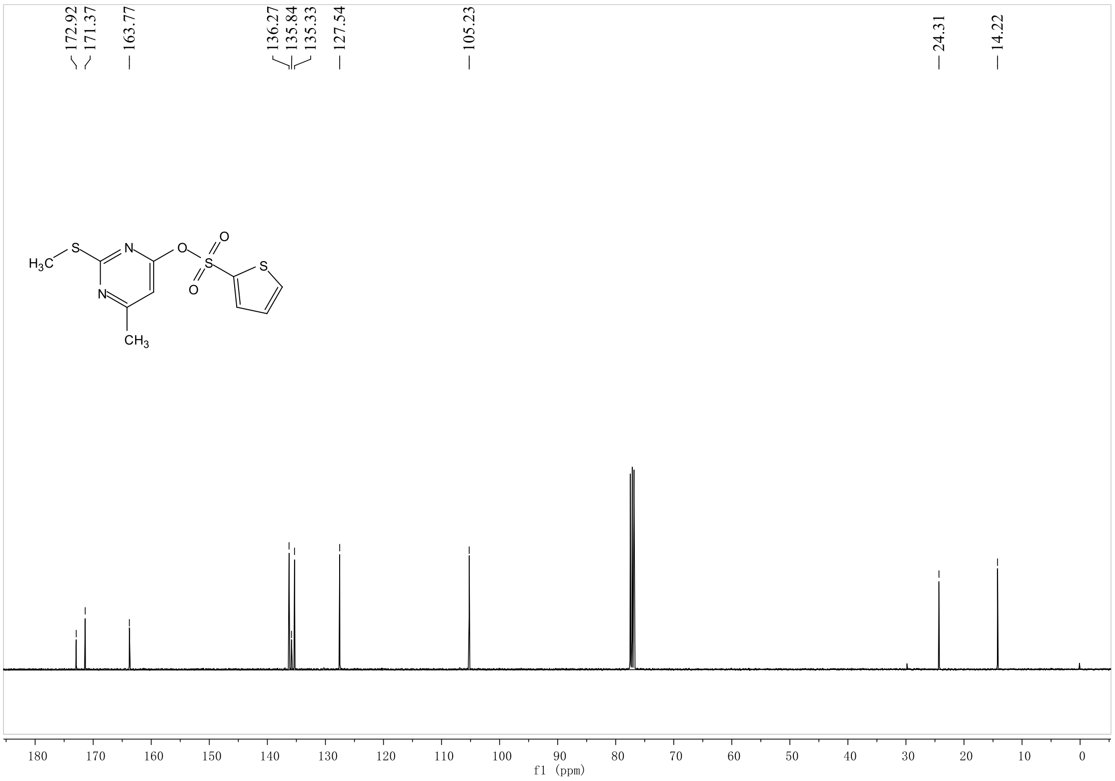

^{13}C NMR of compound A20

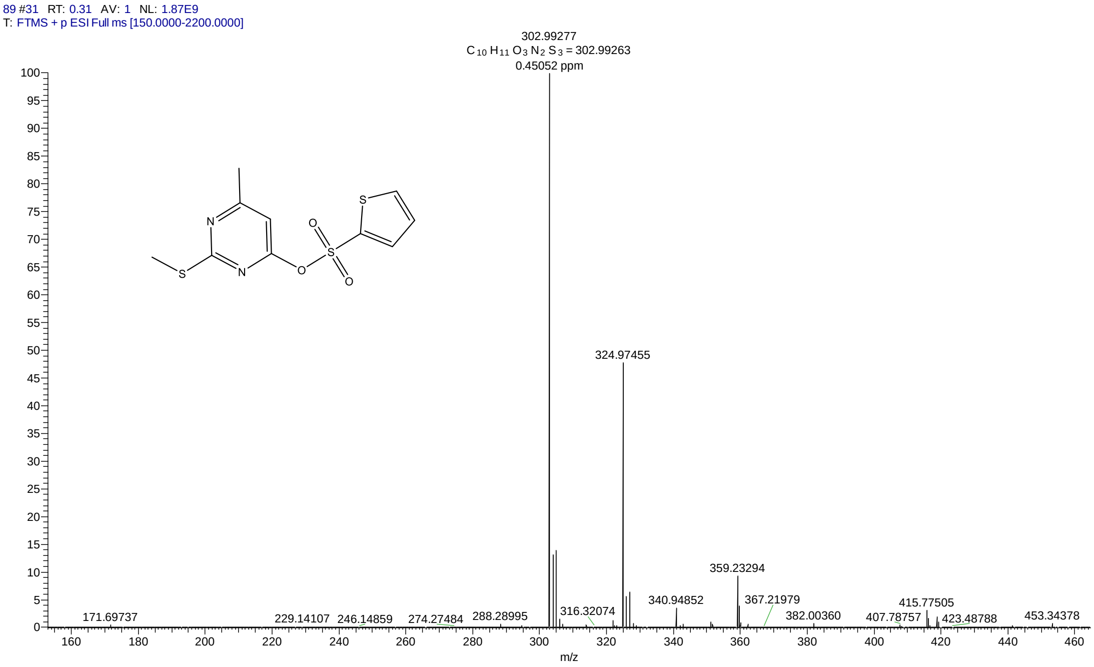

HRMS of compound A20

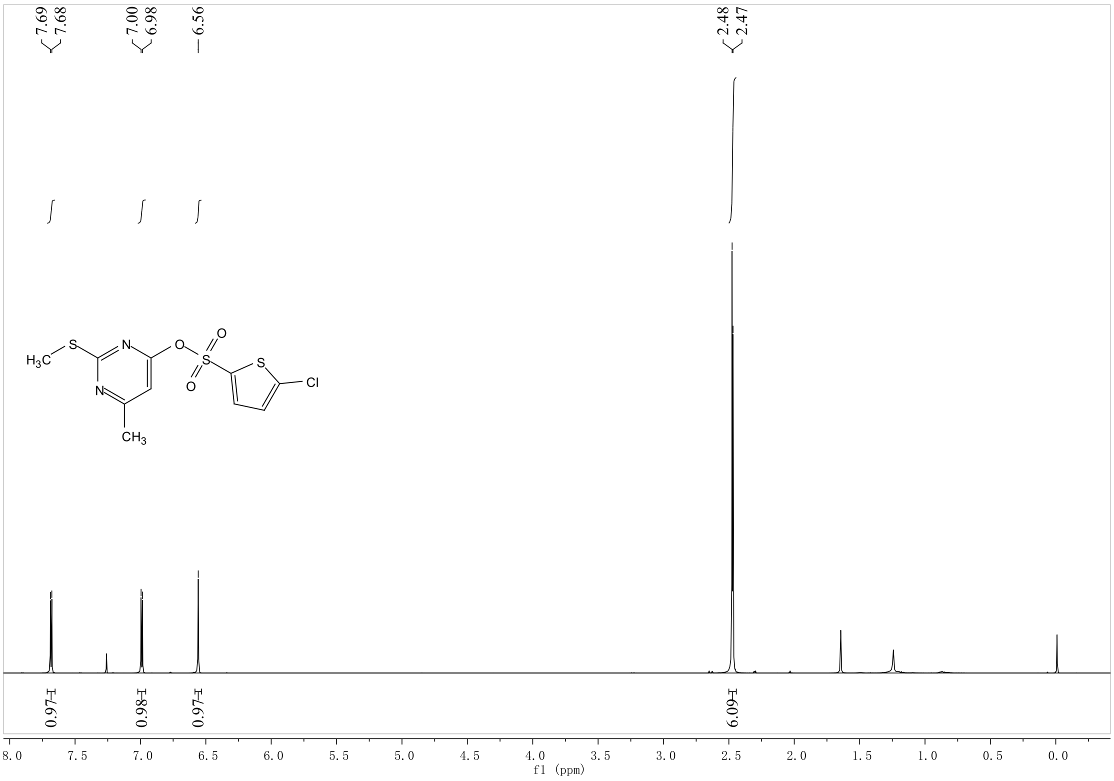

^{1}H NMR of compound A21

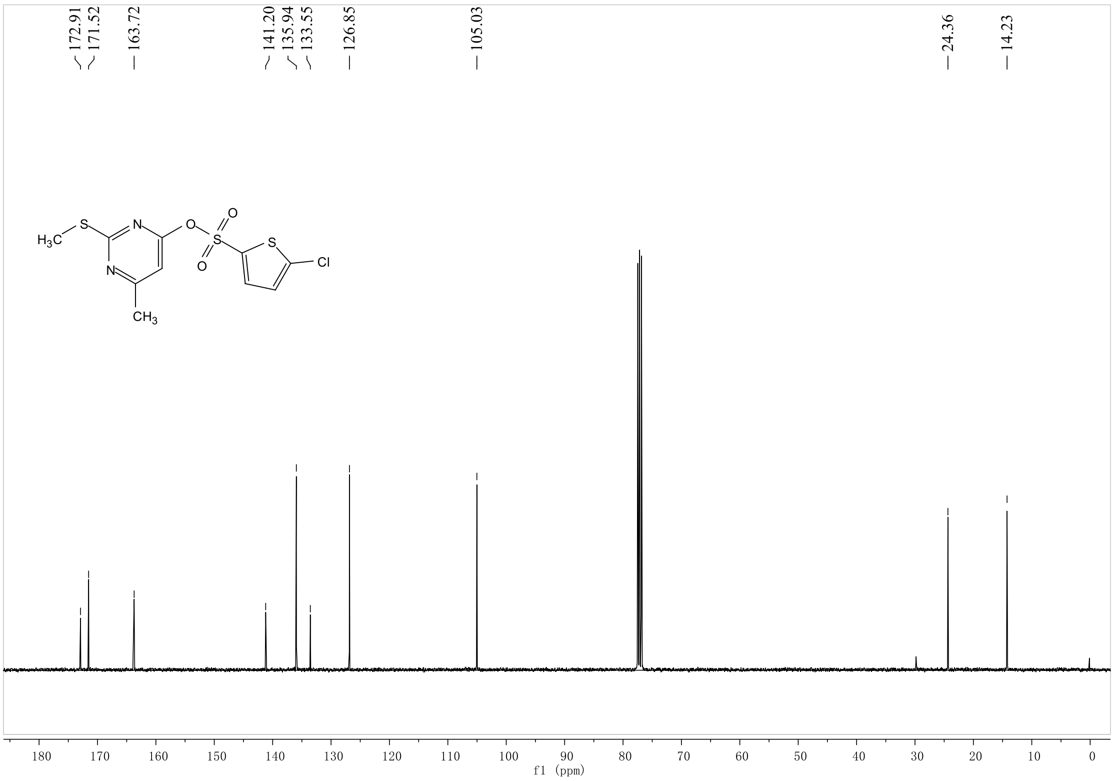

^{13}C NMR of compound A21

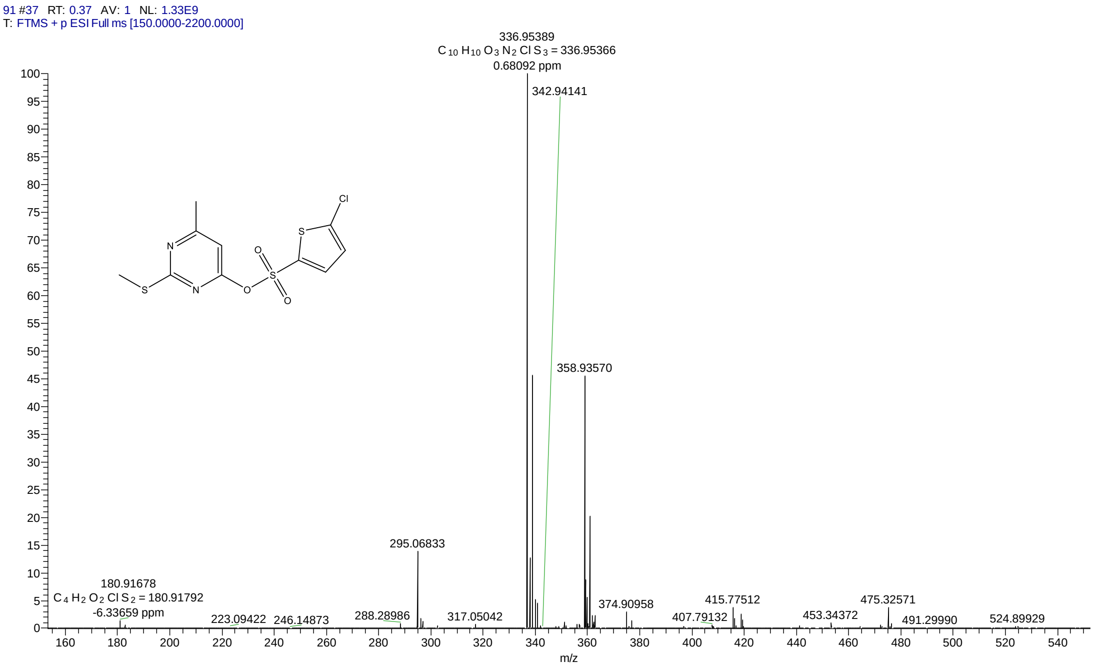

HRMS NMR of compound A21

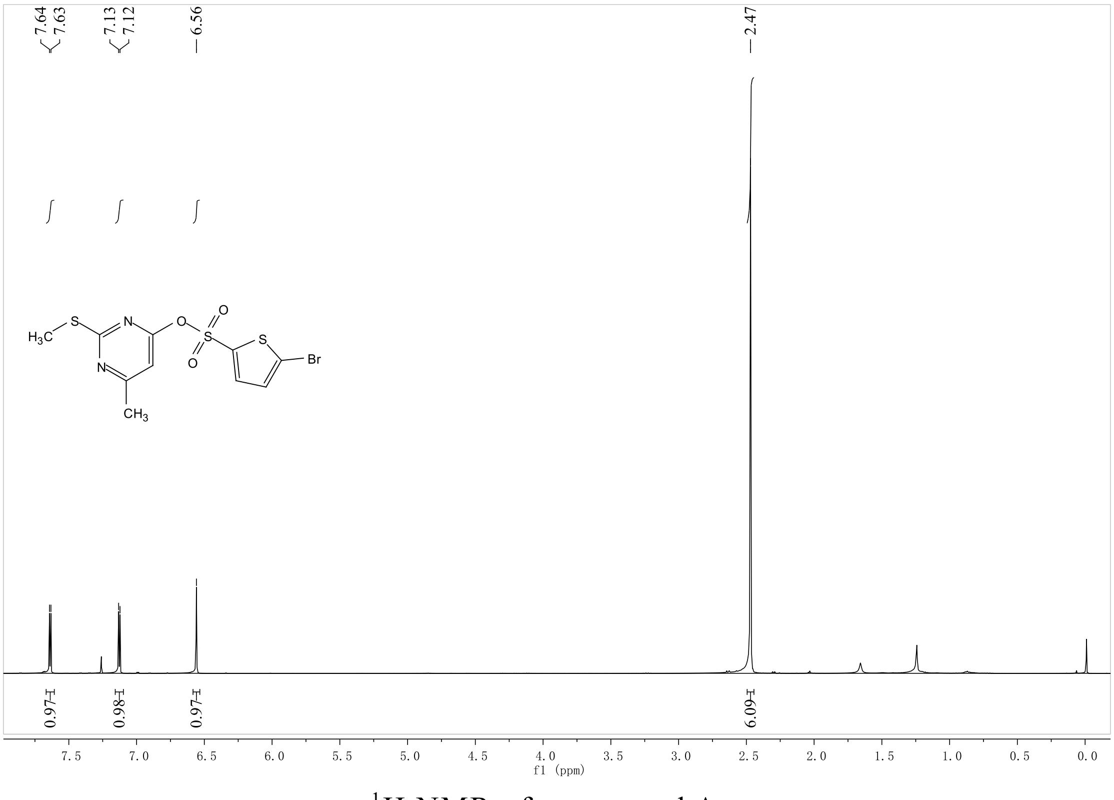

^{1}H NMR of compound A22

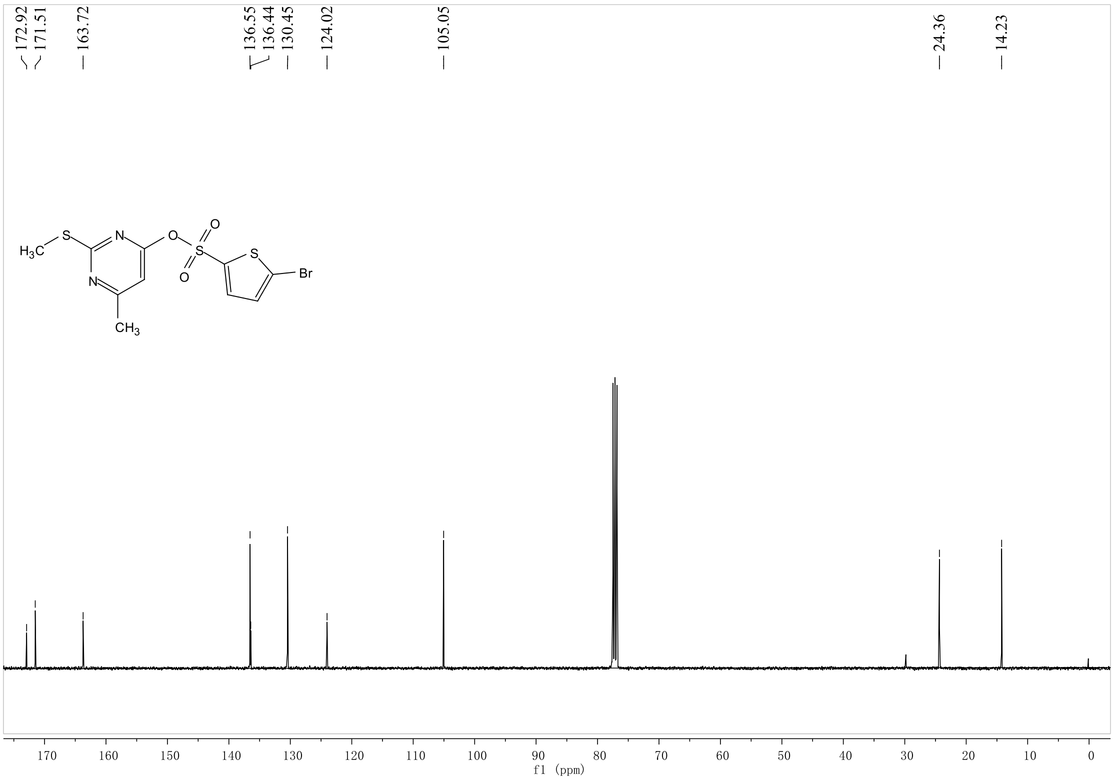

^{13}C NMR of compound A22

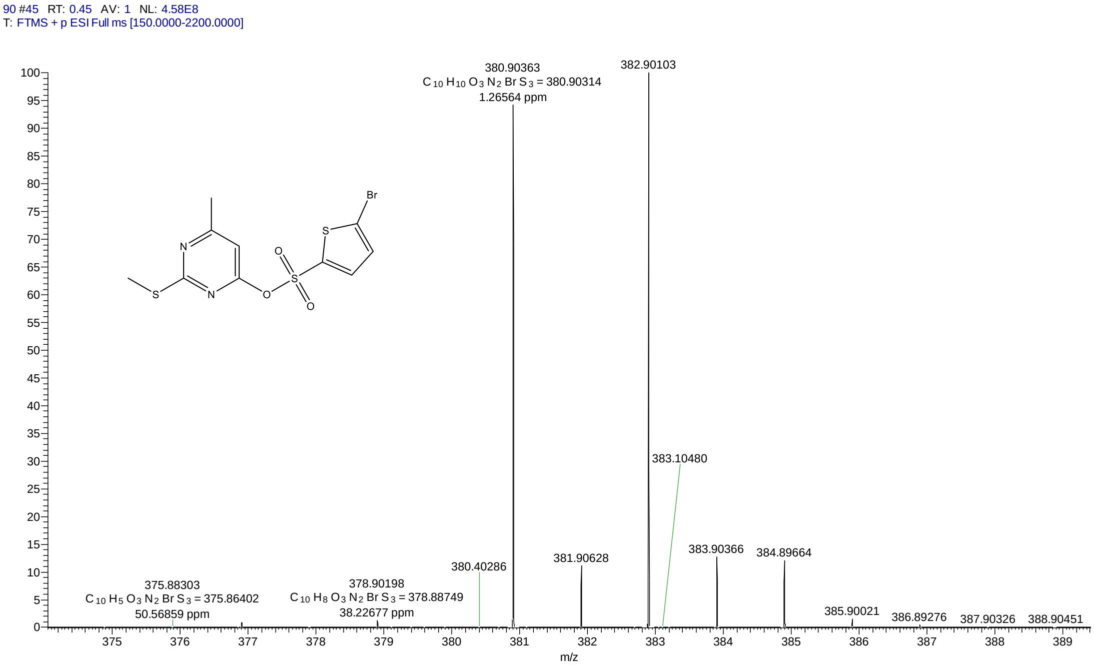

HRMS NMR of compound A22

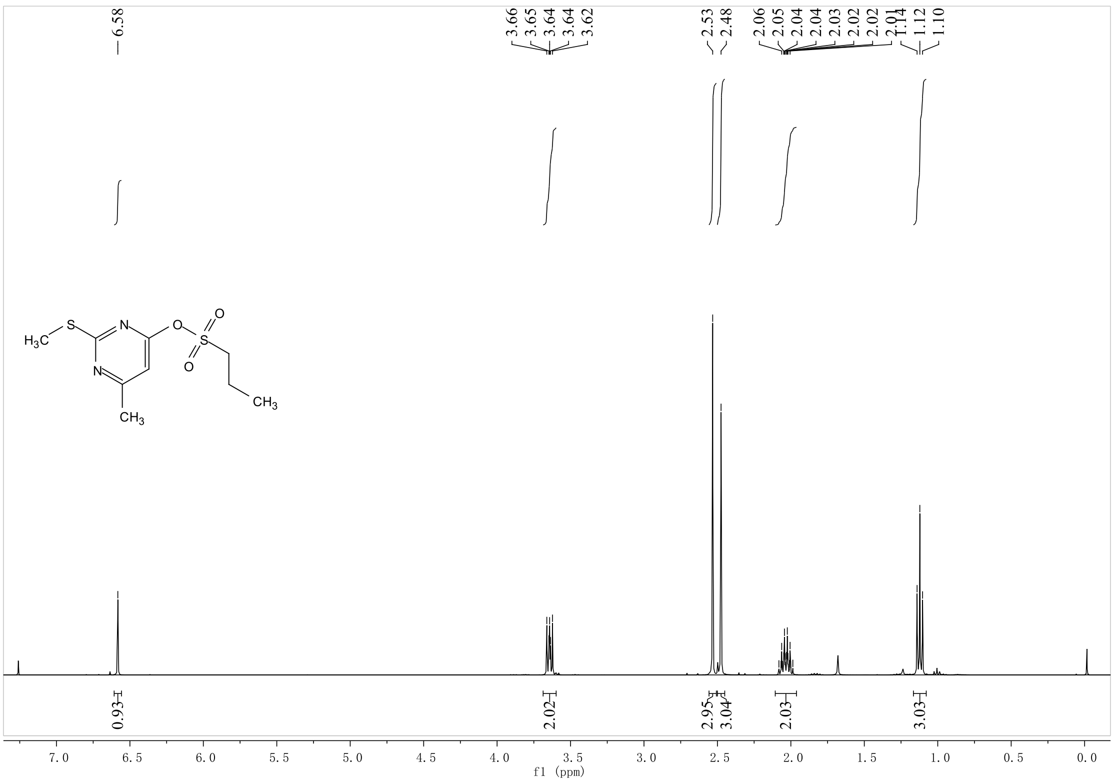

^{1}H NMR of compound A23

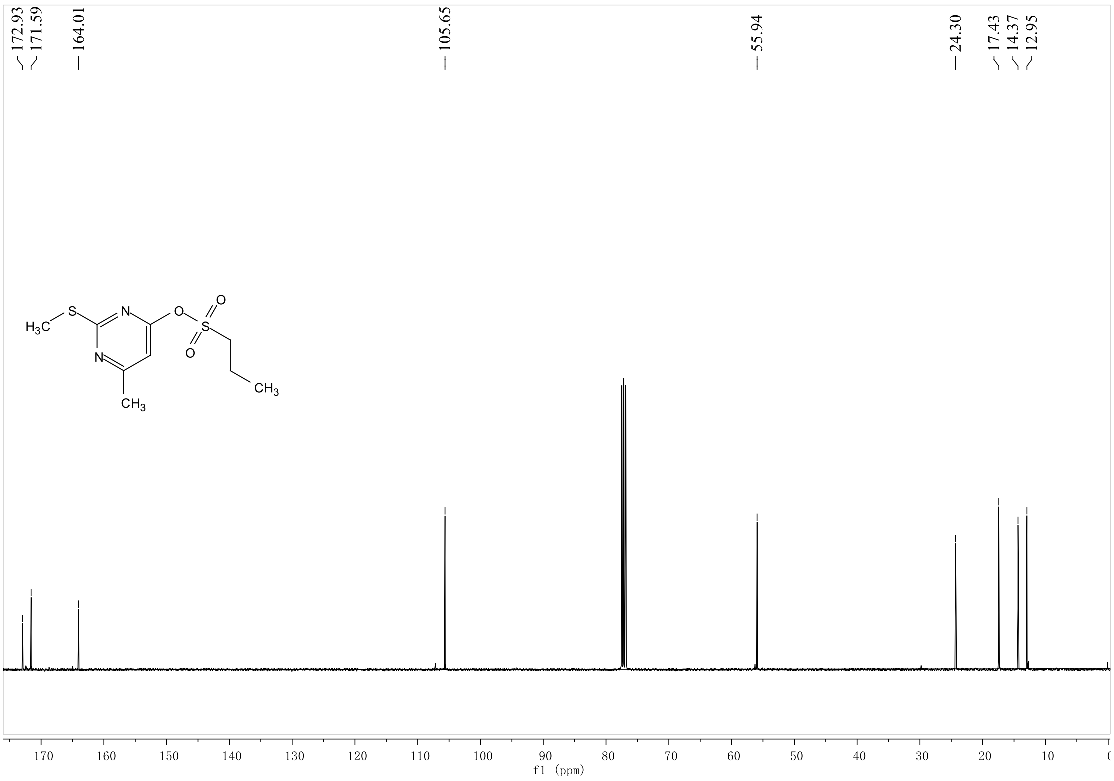

^{13}C NMR of compound A23

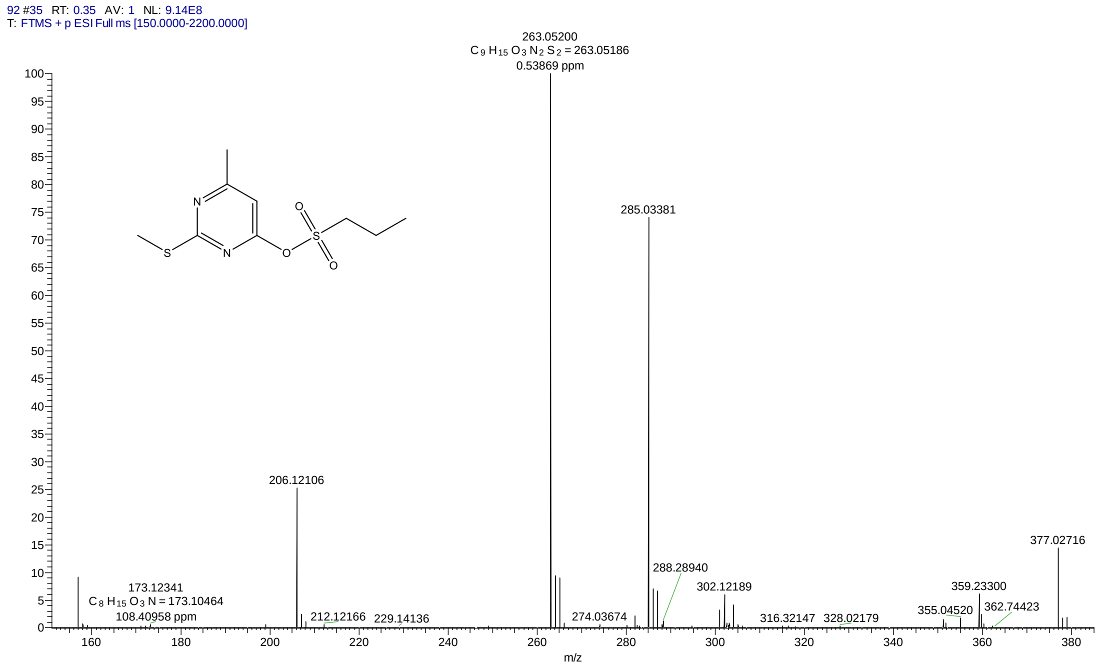

HRMS of compound A23

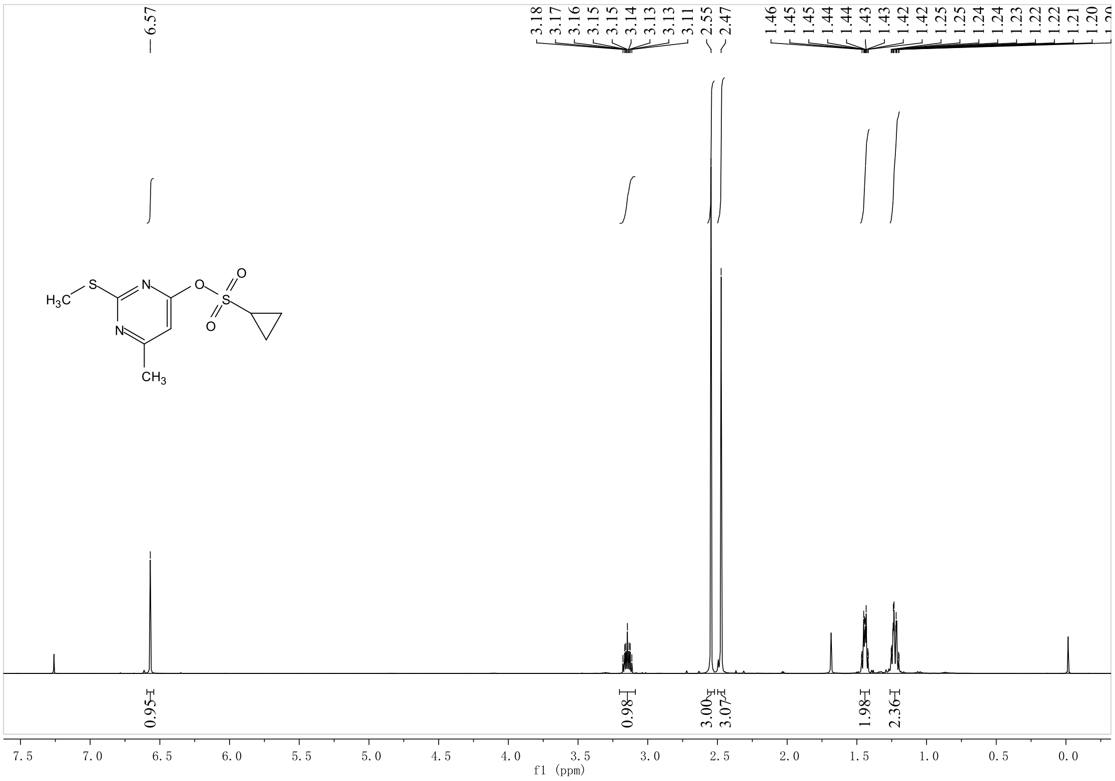

^{1}H NMR of compound A24

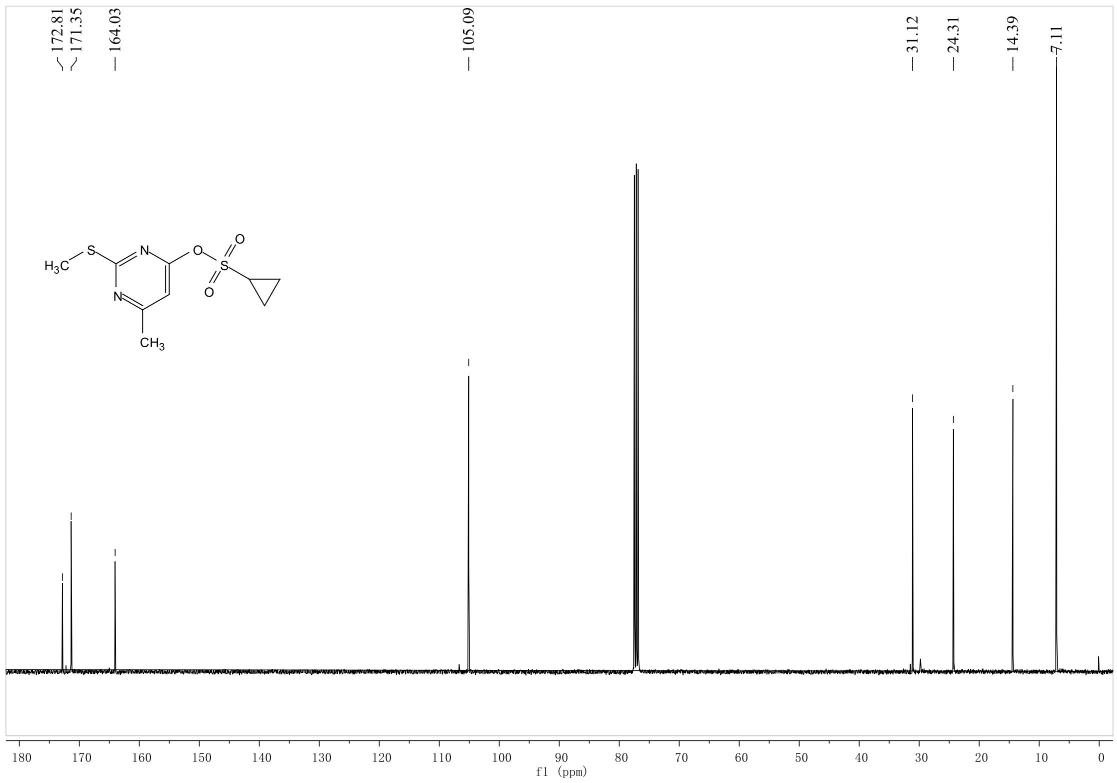

^{13}C NMR of compound A24

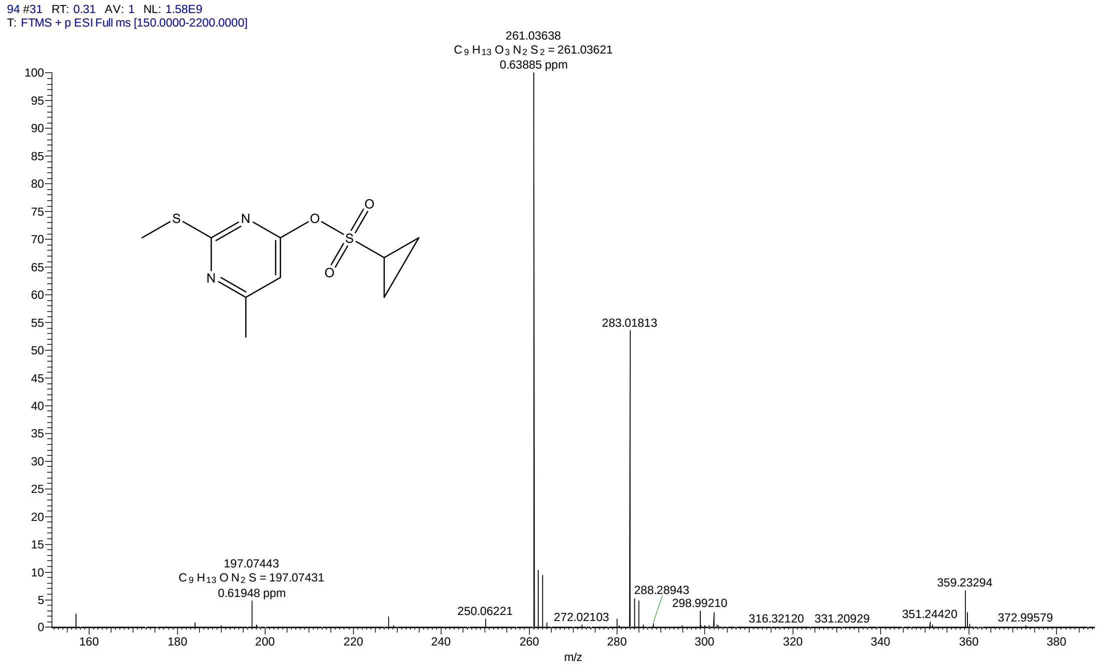

HRMS NMR of compound A24

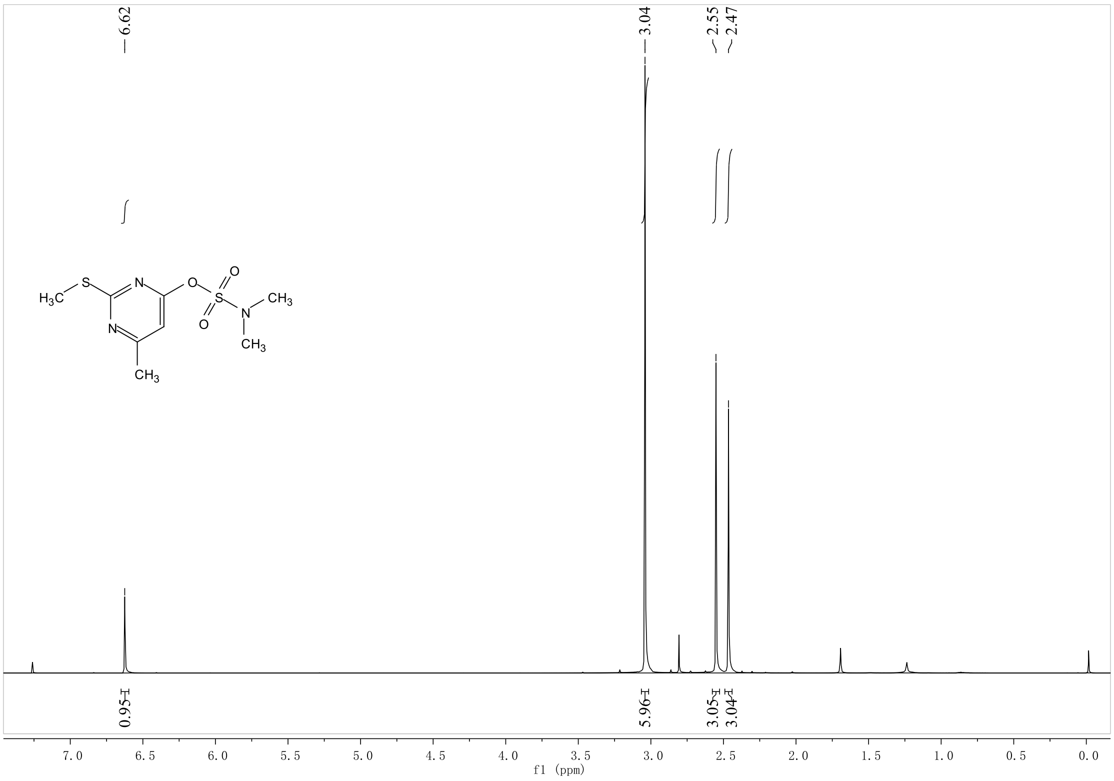

^{1}H NMR of compound A25

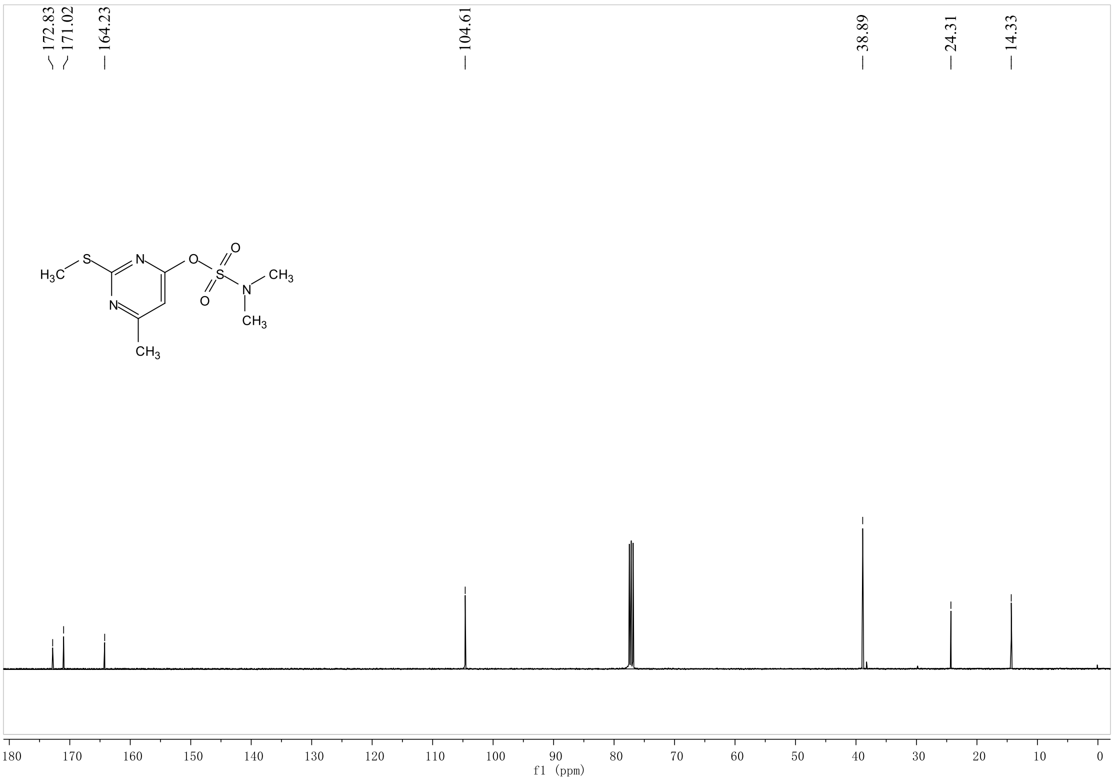

^{13}C NMR of compound A25

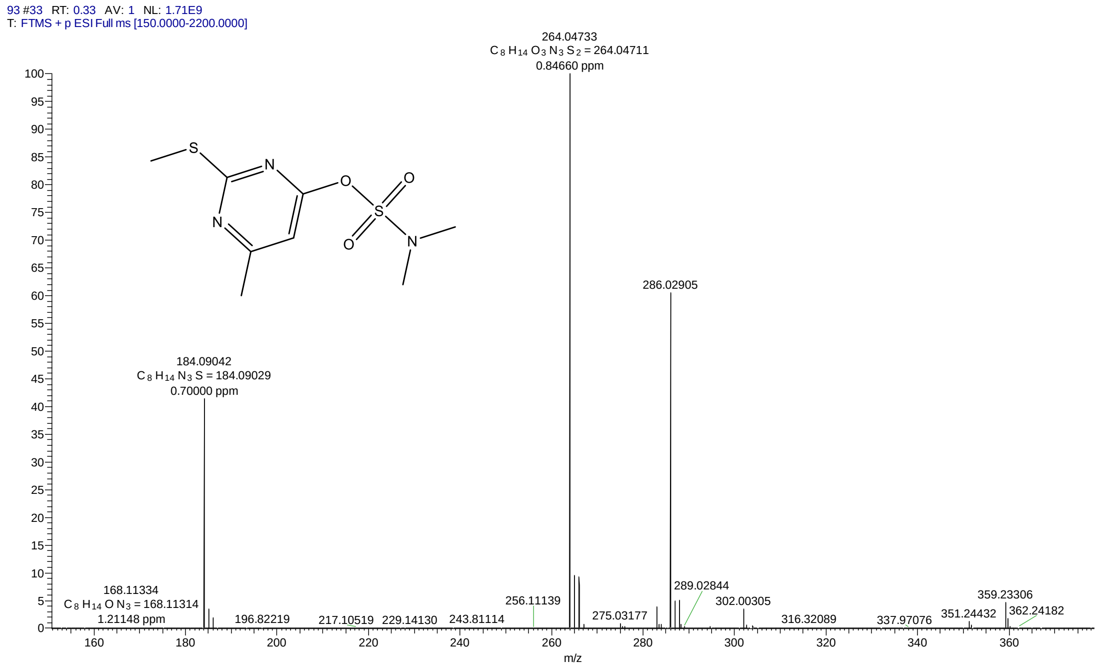

HRMS of compound A25

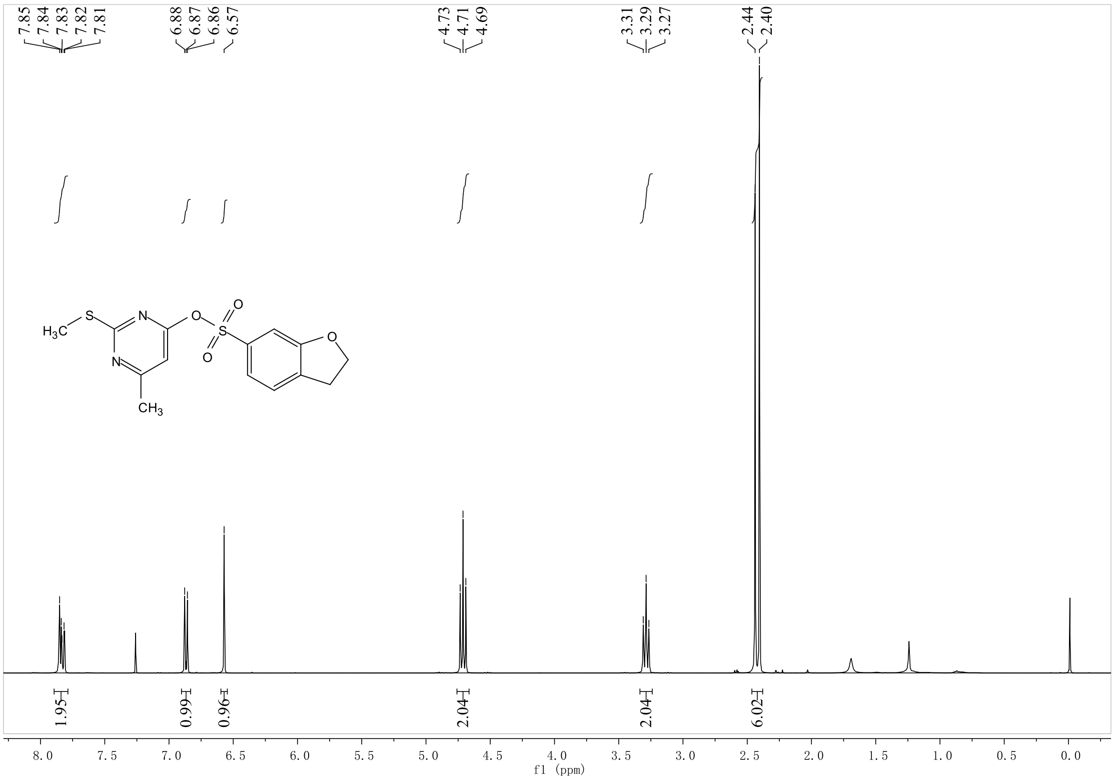

^{1}H NMR of compound A26

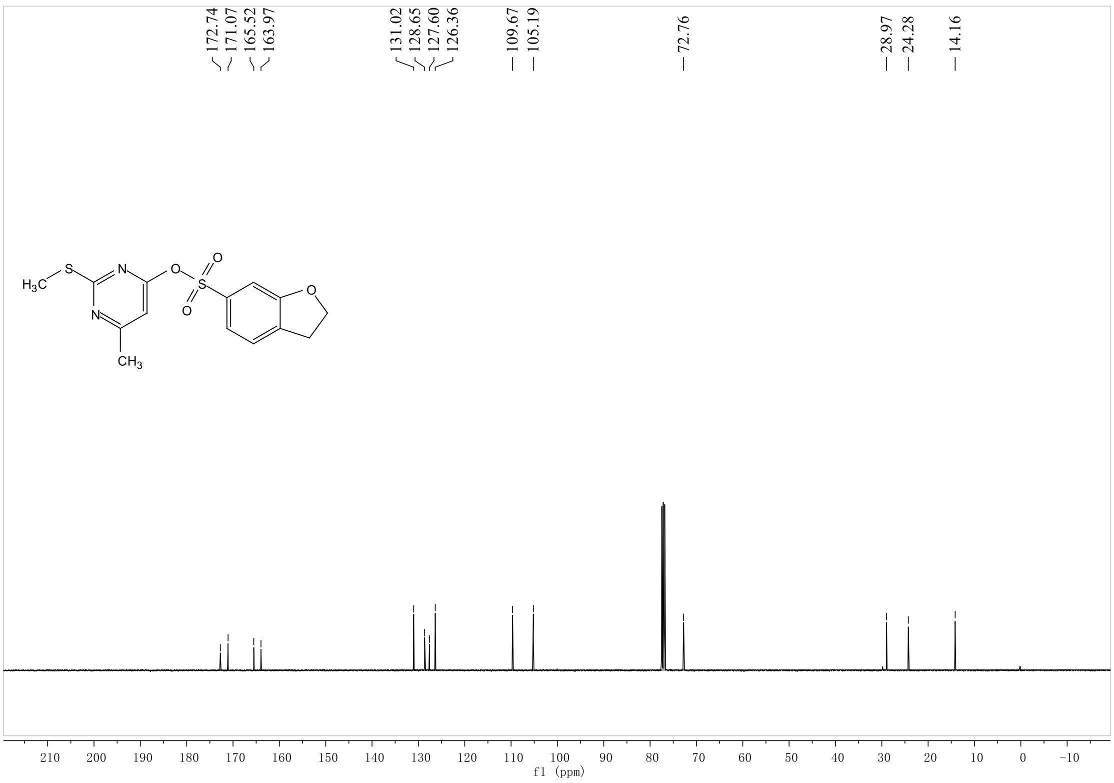

^{13}C NMR of compound A26

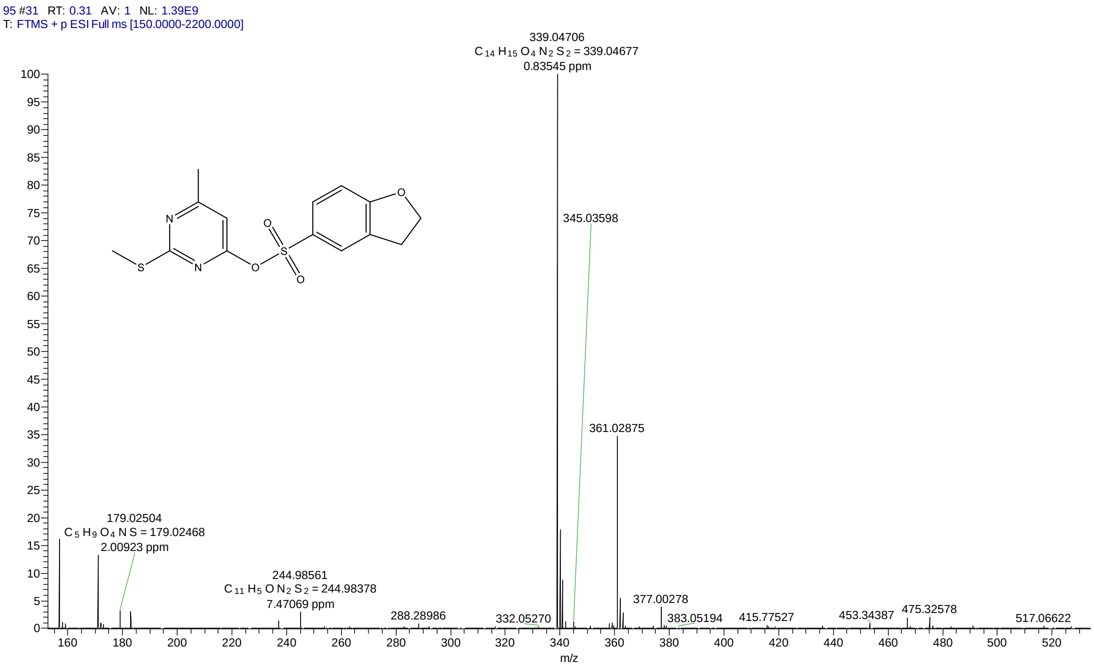

HRMS of compound A26

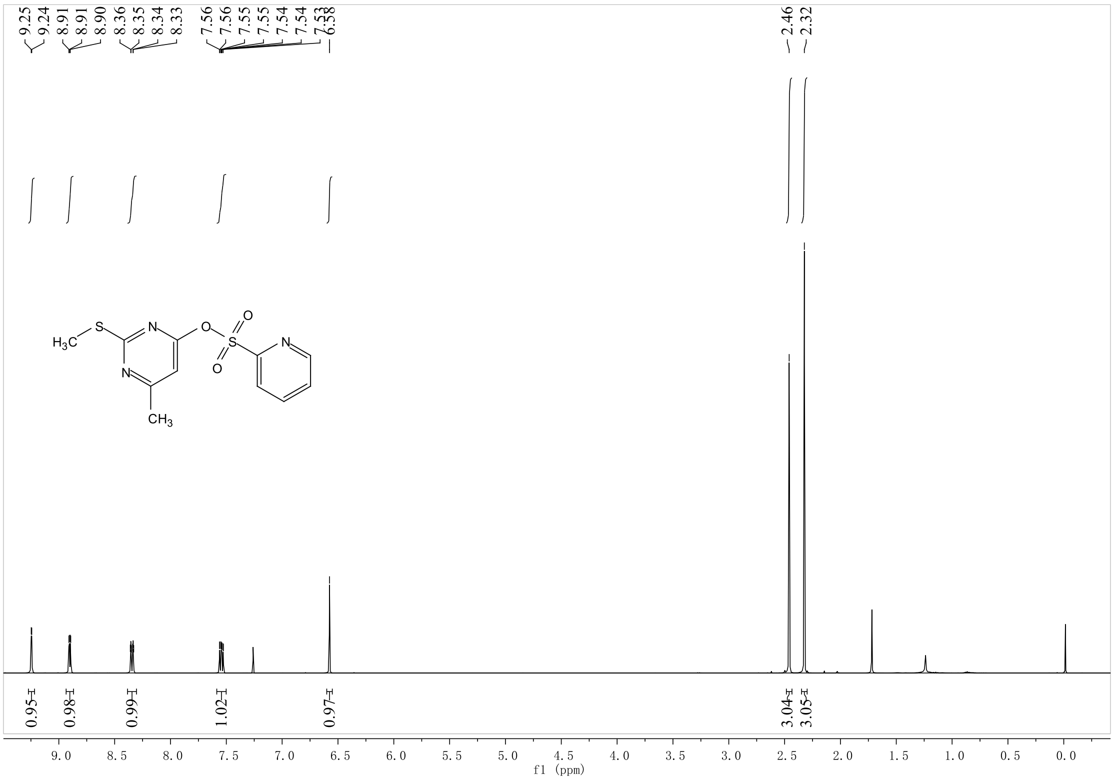

^{1}H NMR of compound A27

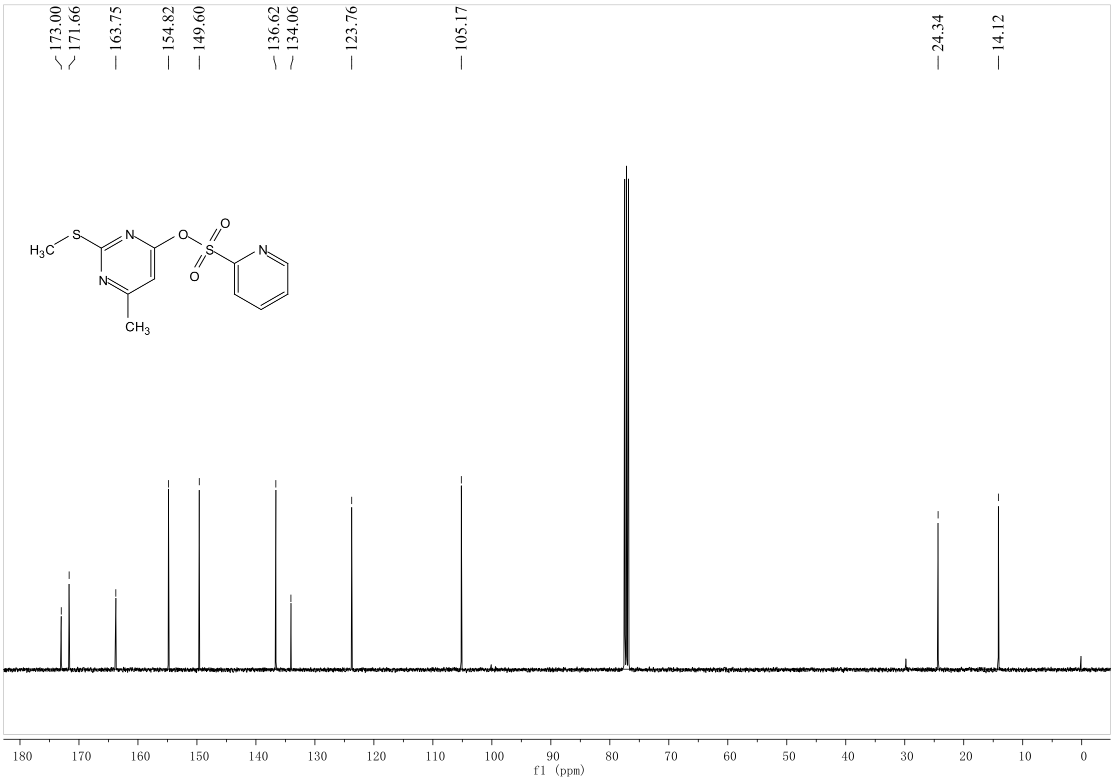

^{13}C NMR of compound A27

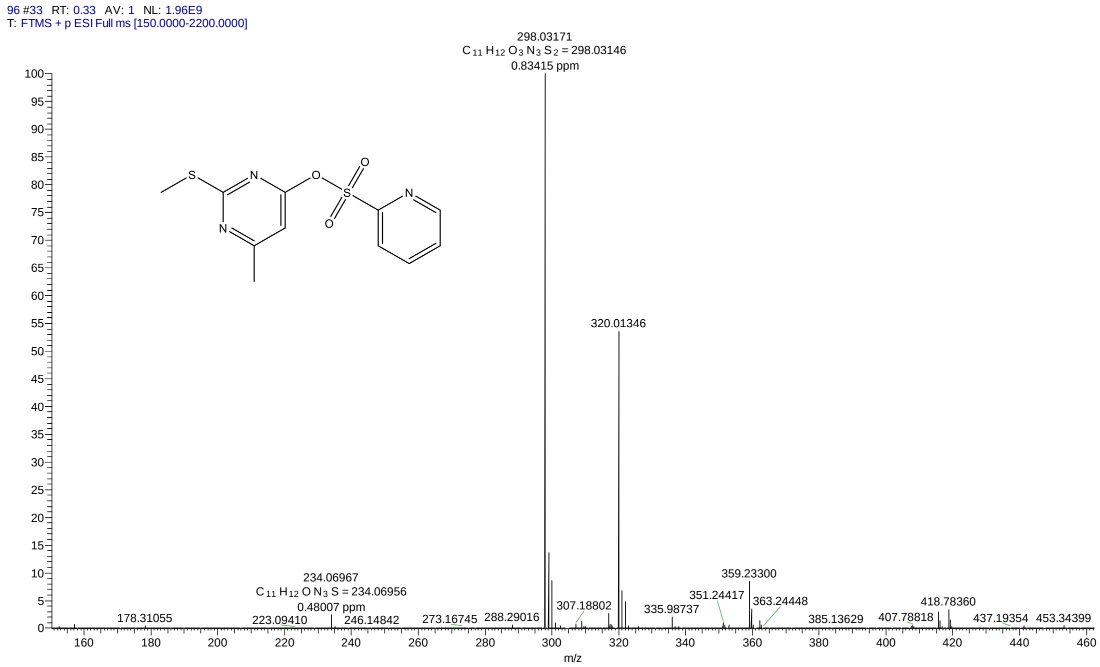

HRMS of compound A_{27}

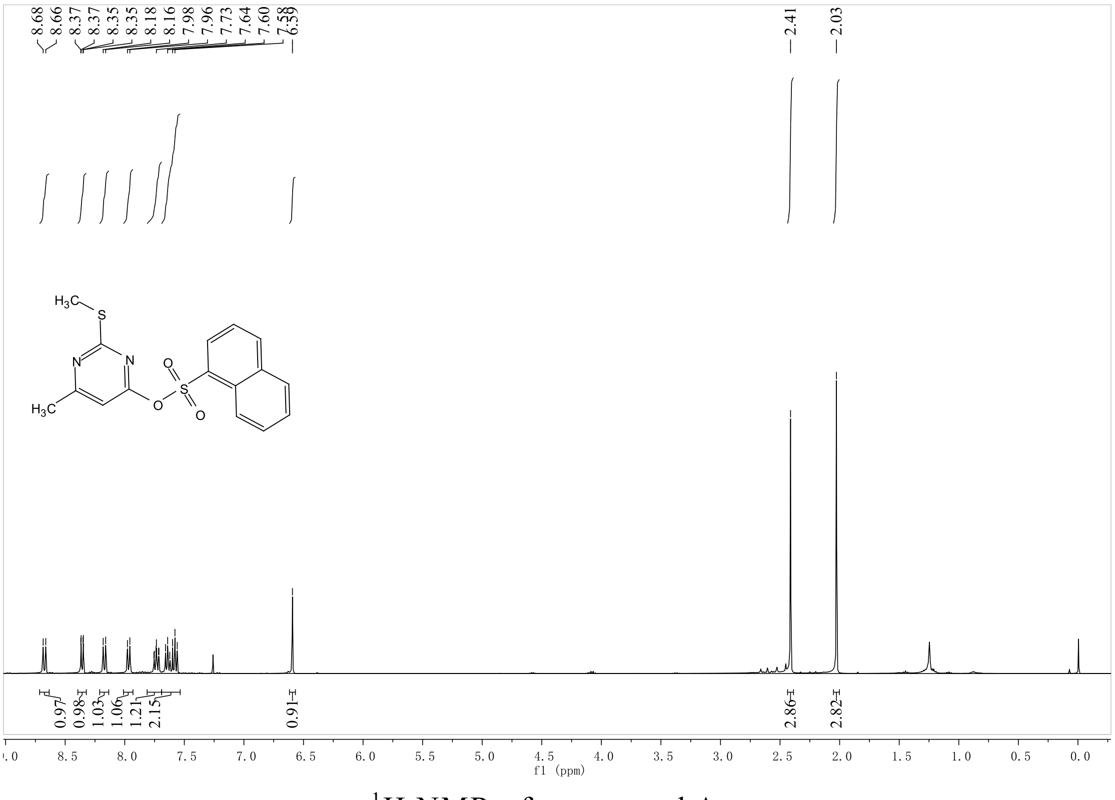

^{1}H NMR of compound A_{28}

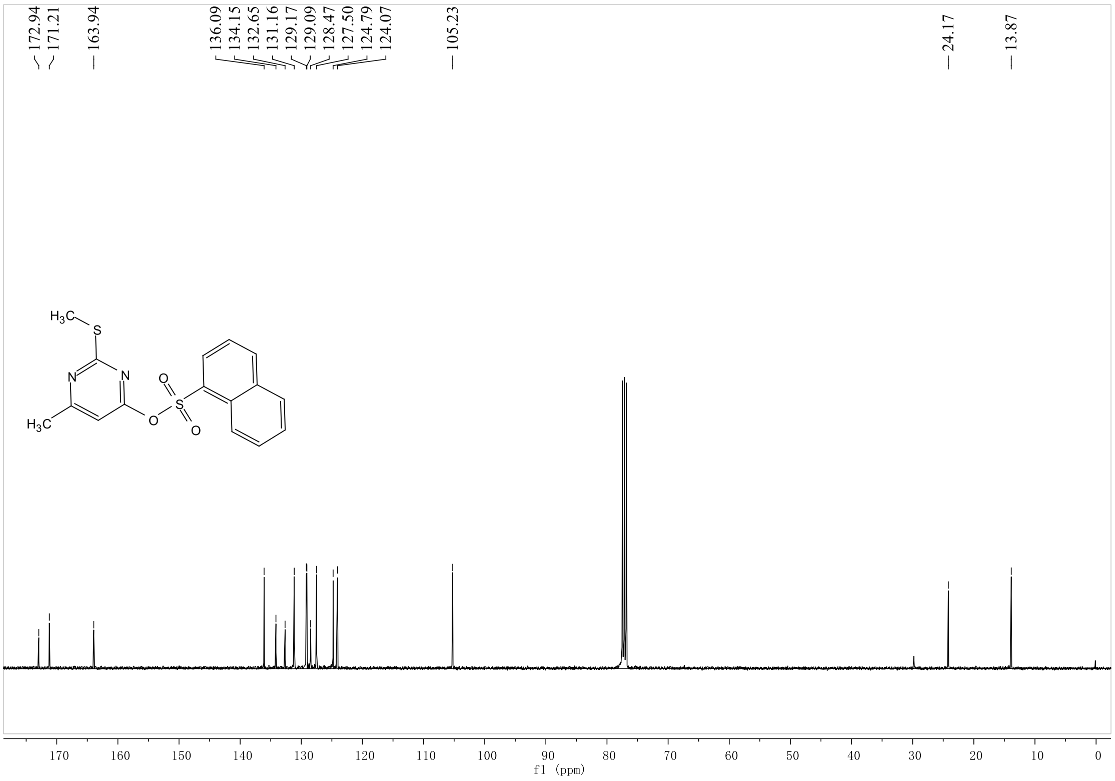

^{13}C NMR of compound A_{28}

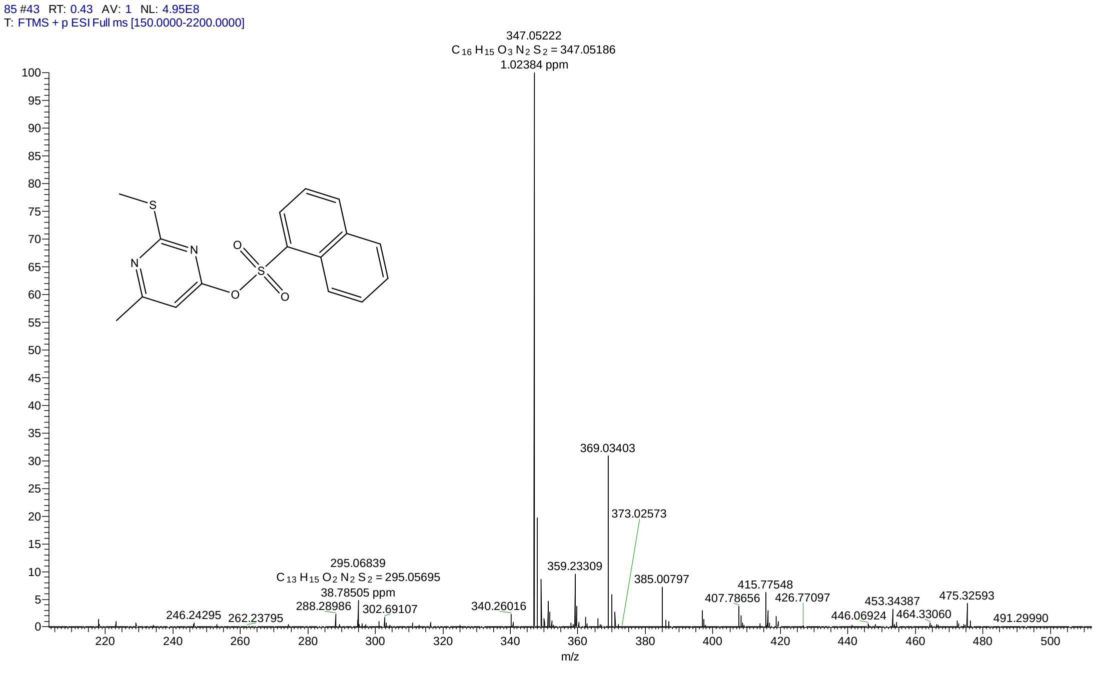

HRMS of compound A_{28}

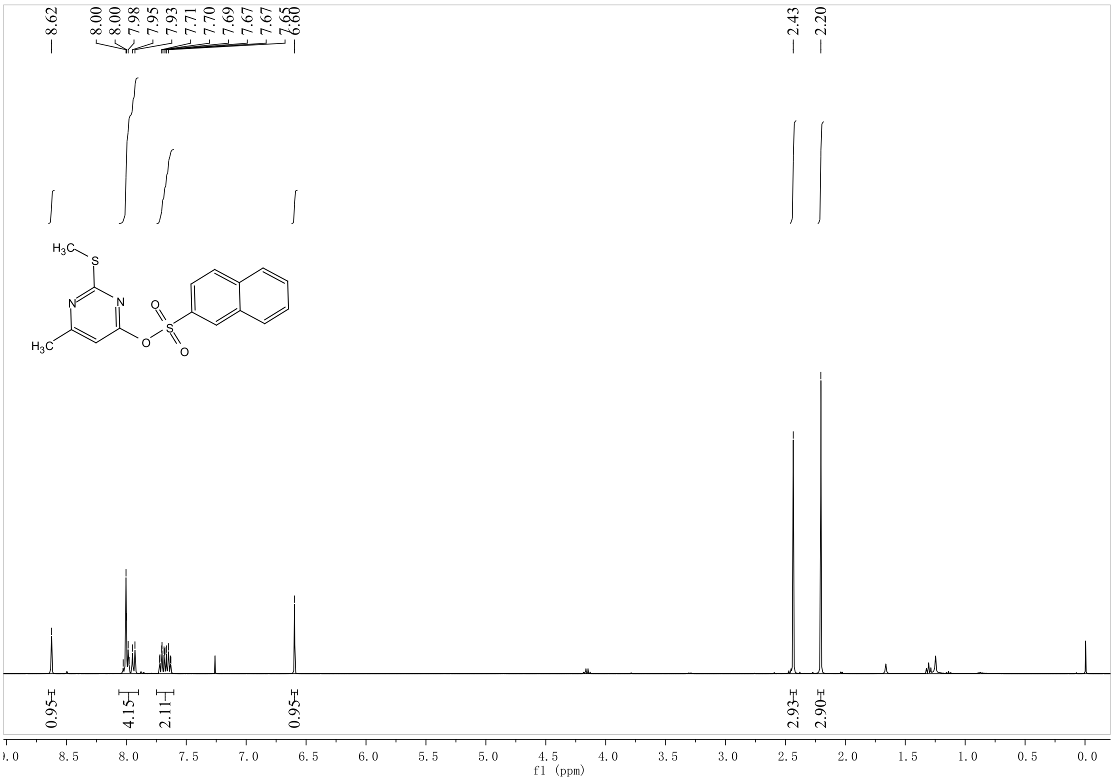

^{1}H NMR of compound A29

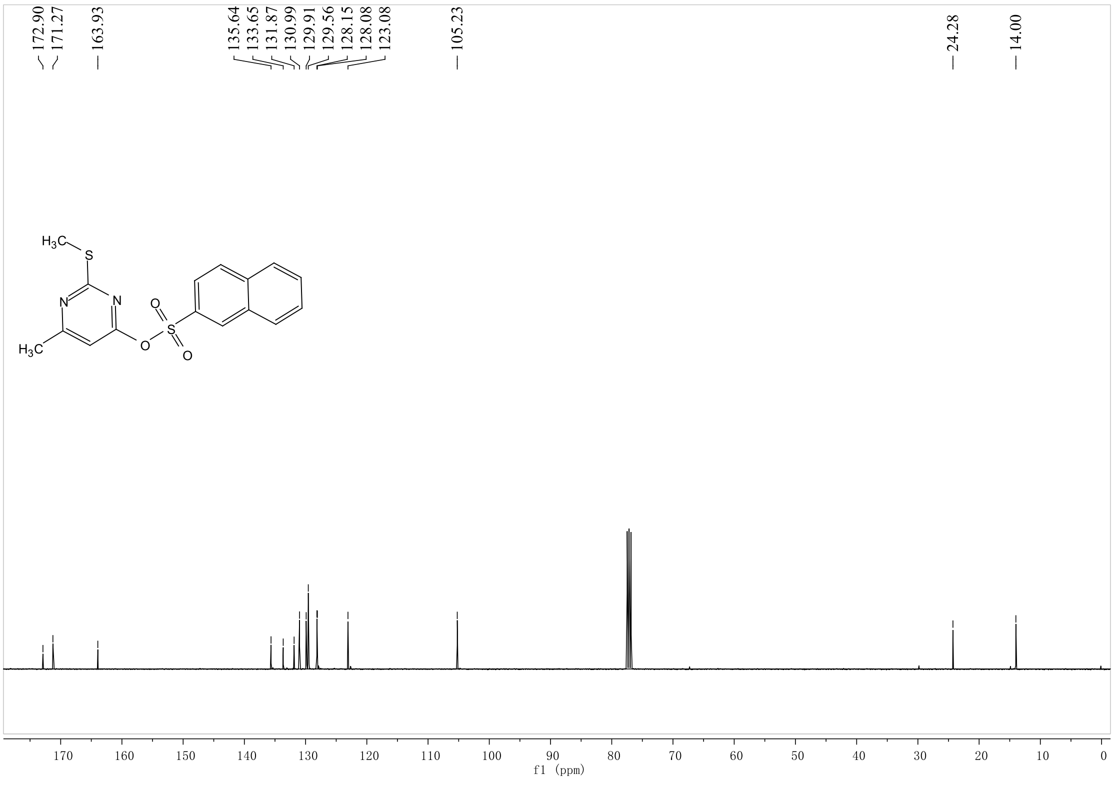

^{13}C NMR of compound A29

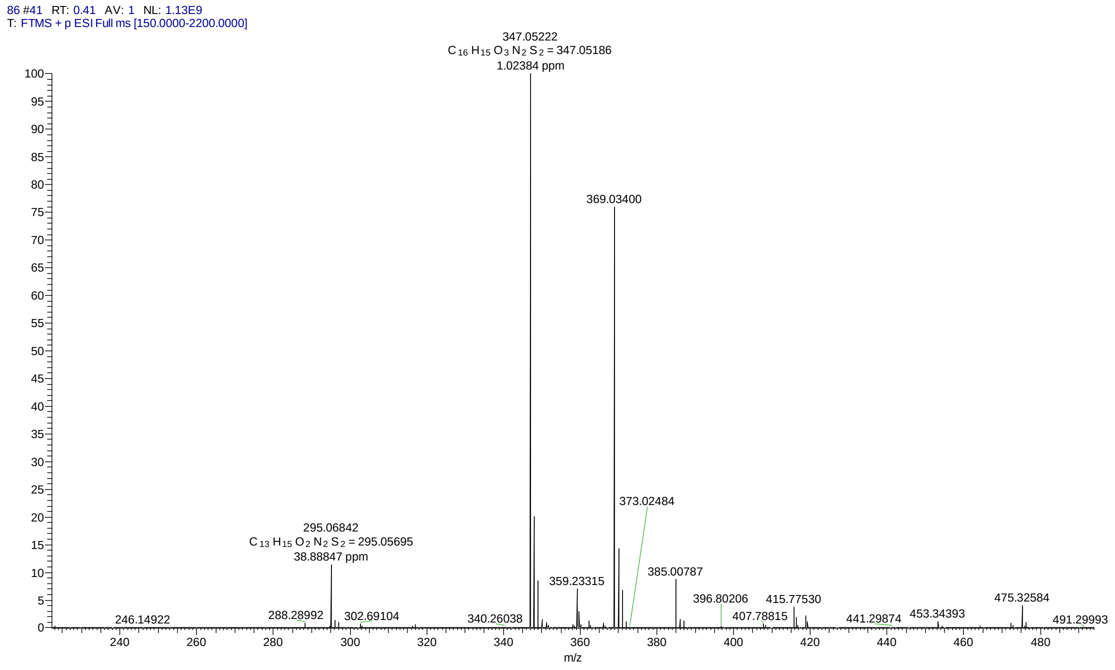

HRMS of compound A29

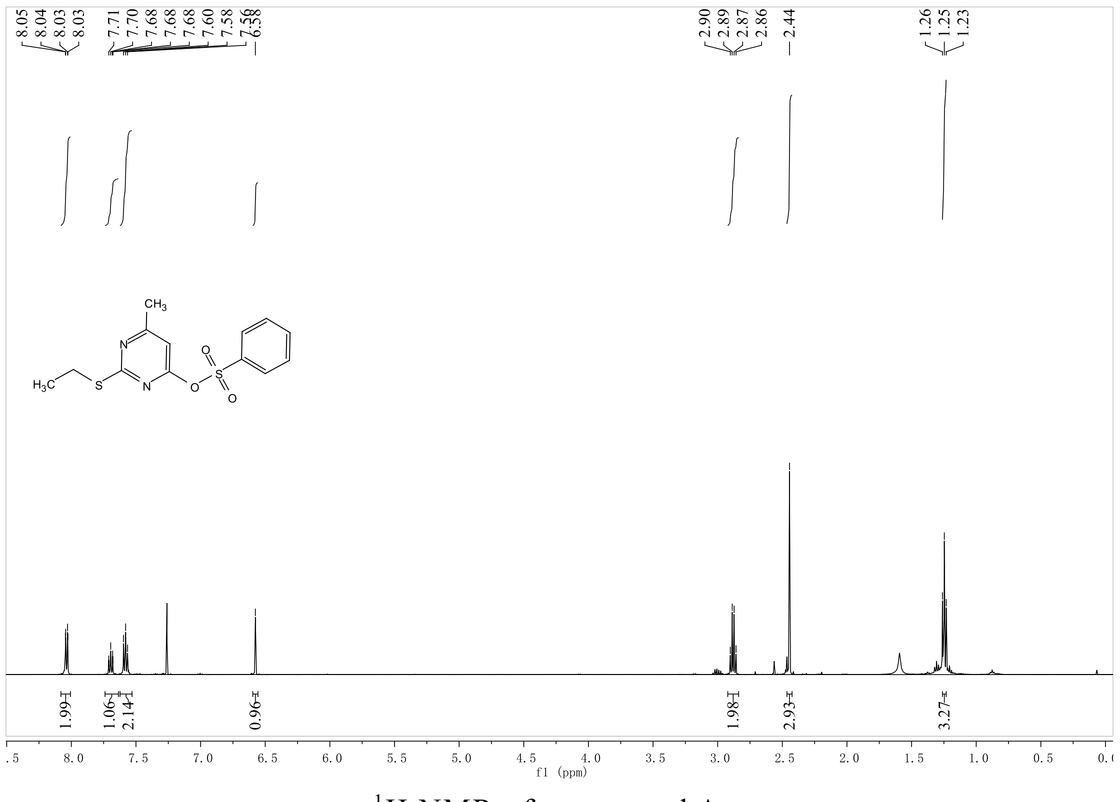

^{1}H NMR of compound A30

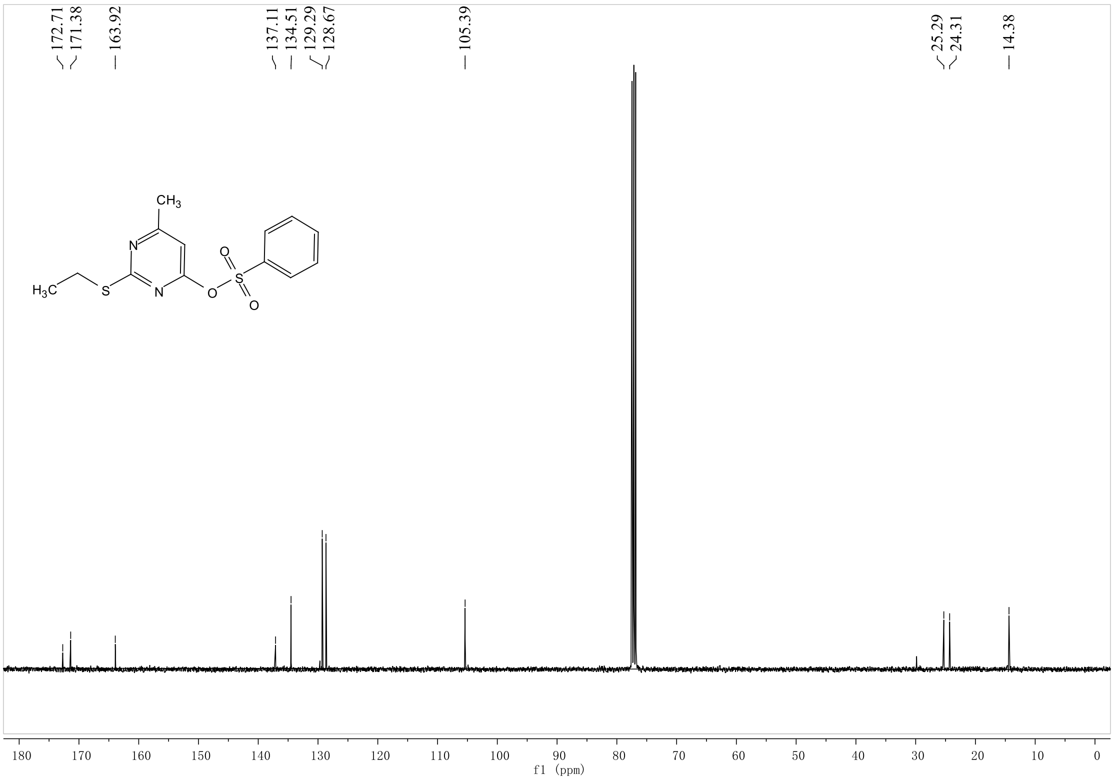

^{13}C NMR of compound A30

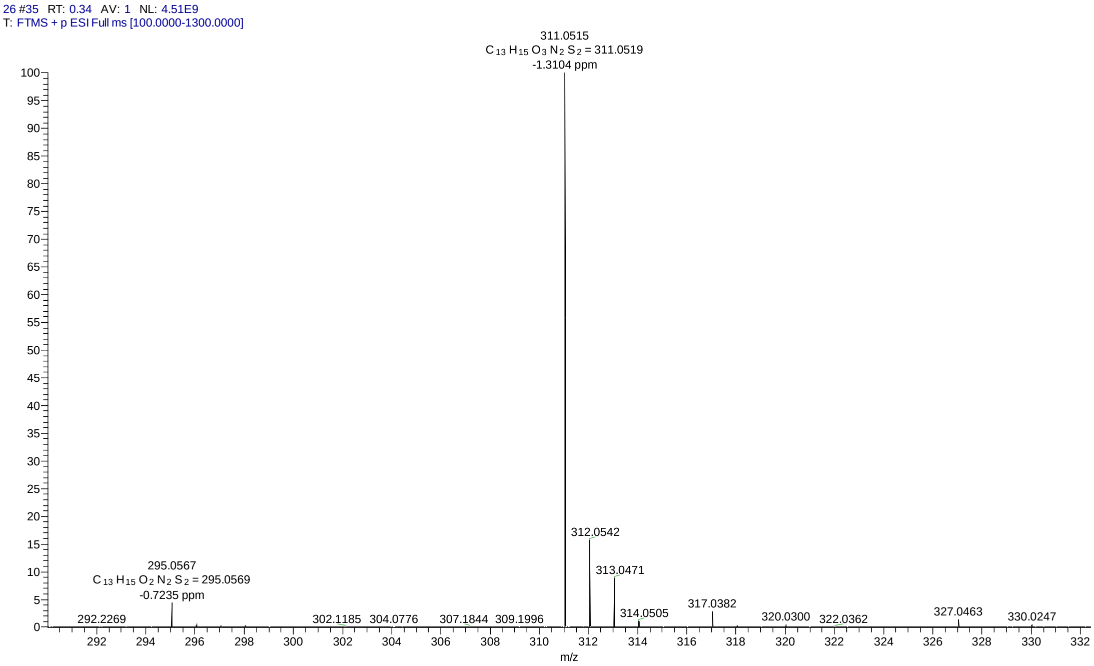

HRMS of compound A30

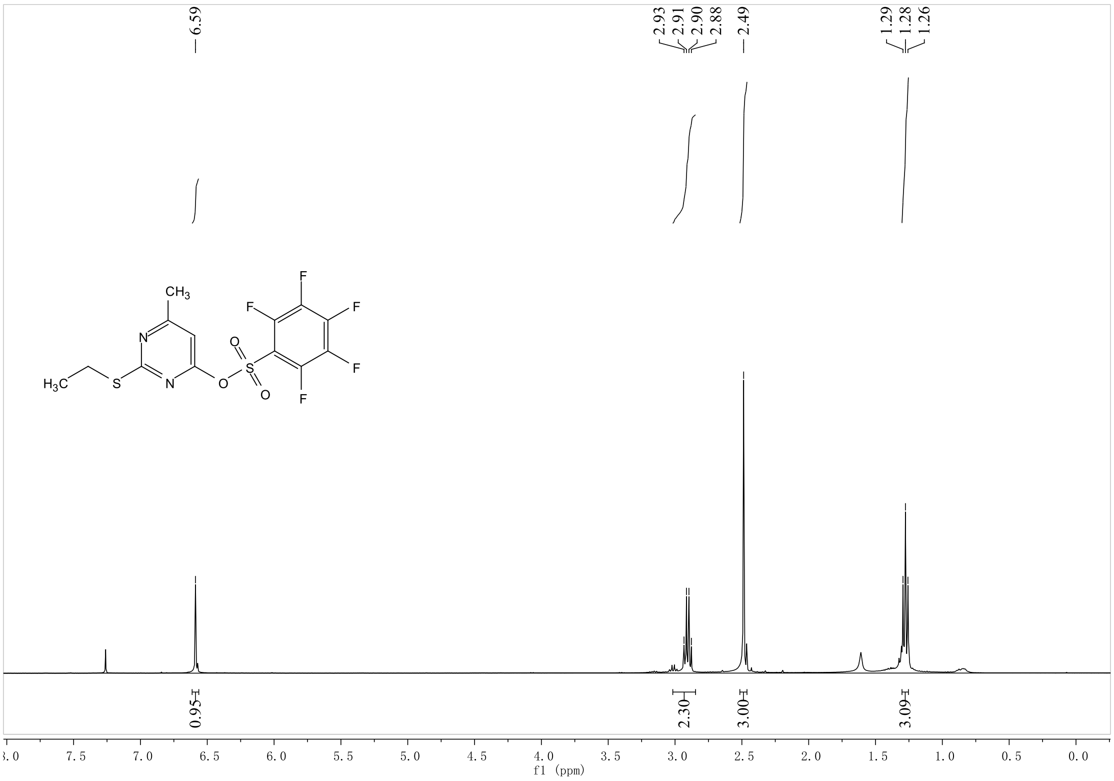

^{1}H NMR of compound A31

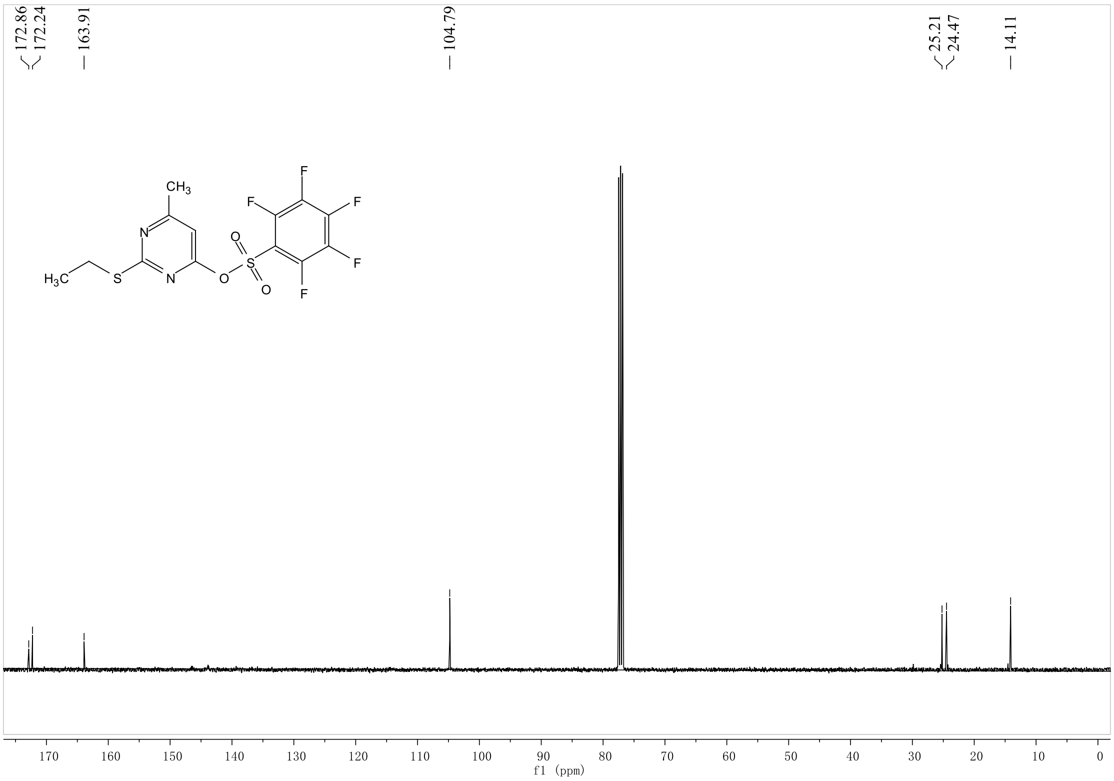

^{13}C NMR of compound A31

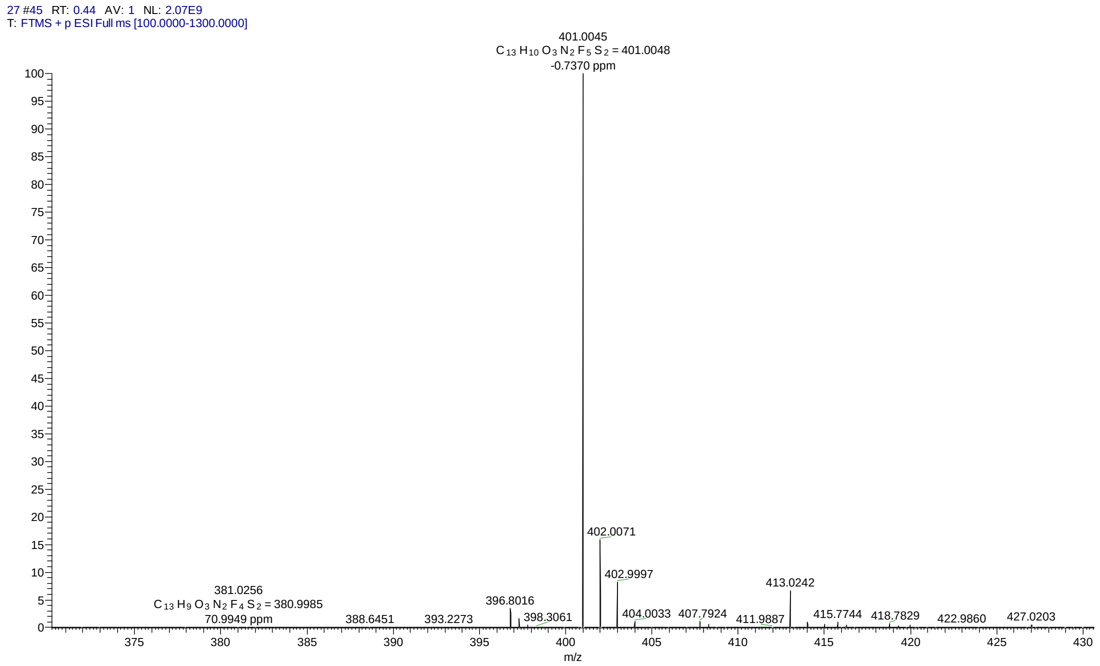

HRMS of compound A31

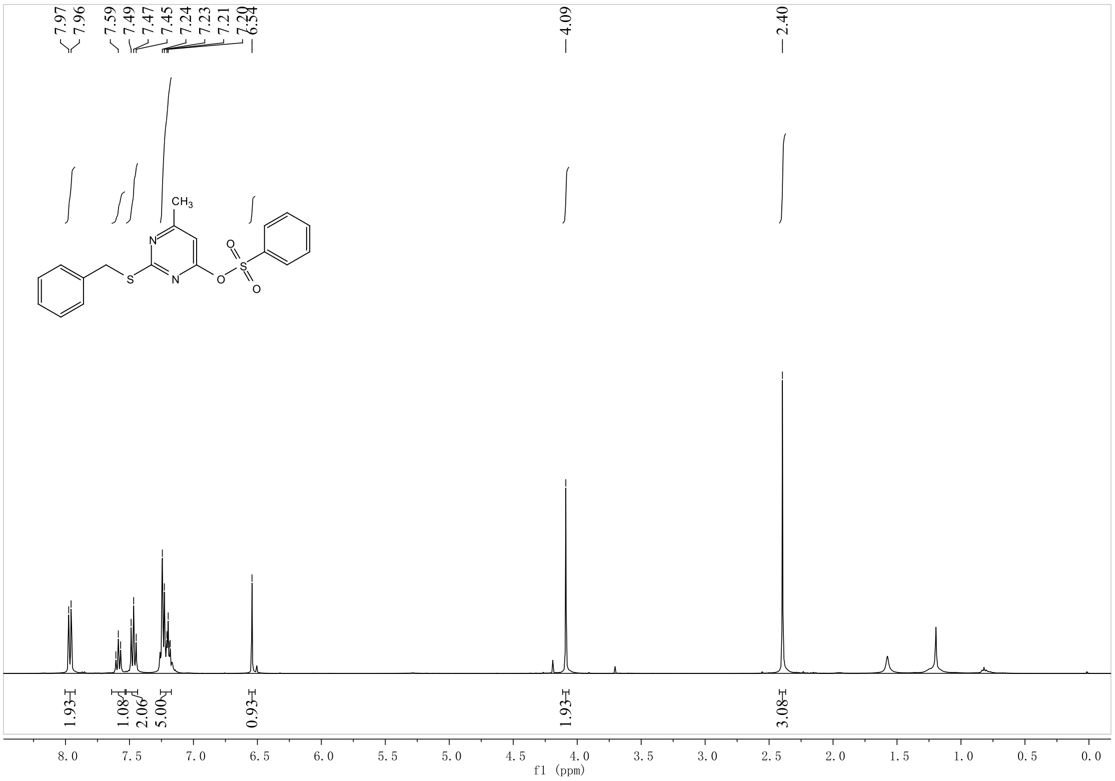

^{1}H NMR of compound A32

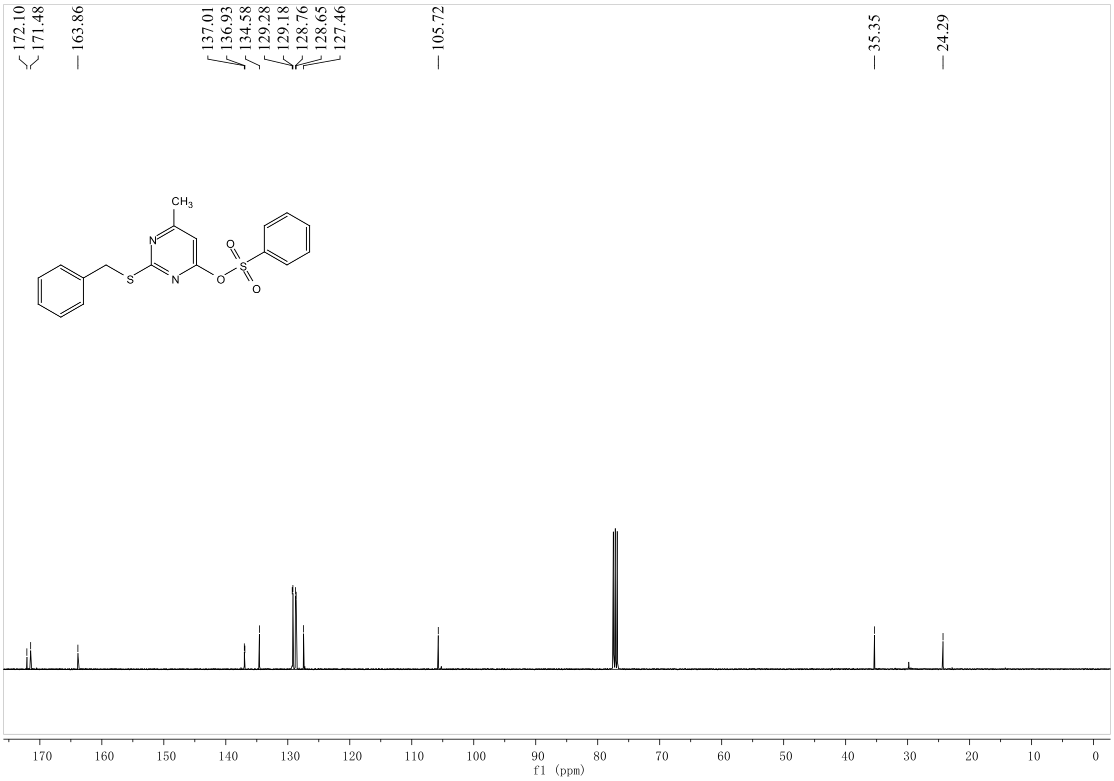

^{13}C NMR of compound A32

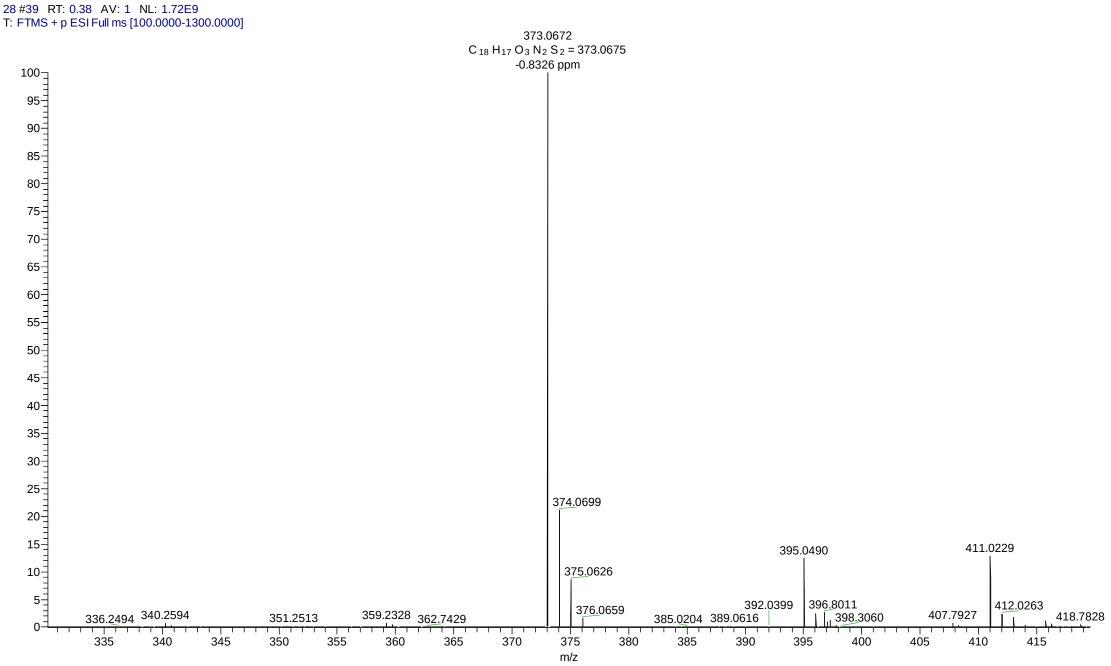

HRMS of compound A32

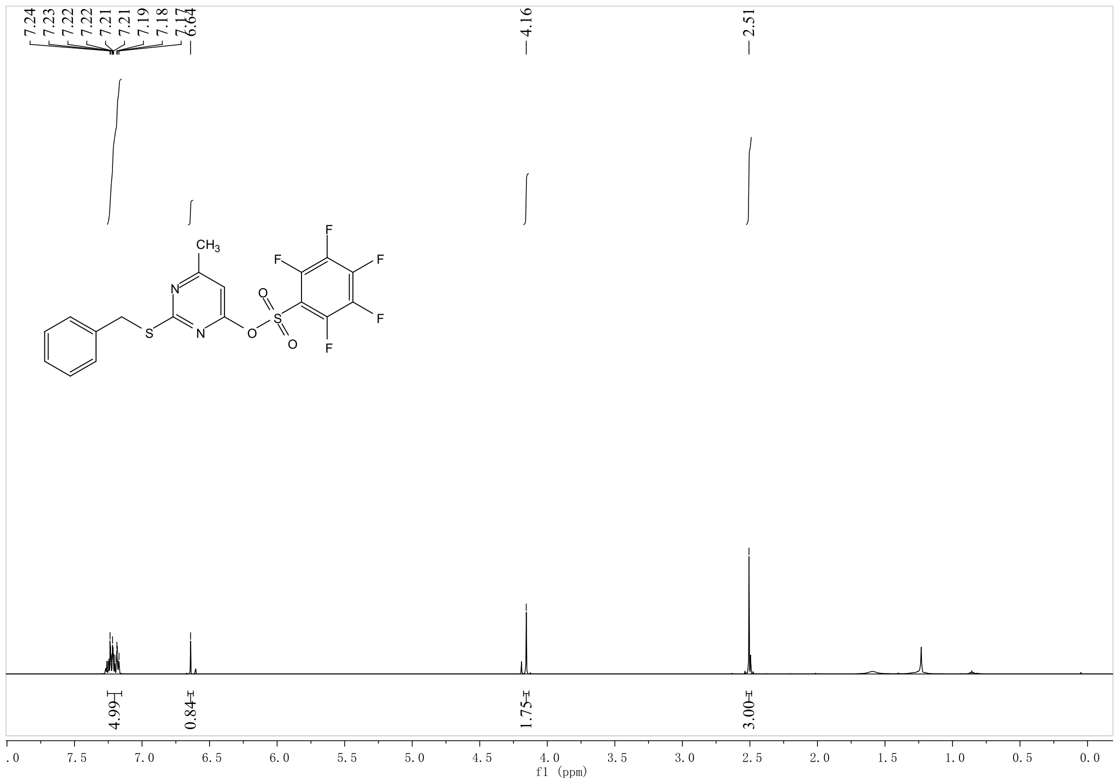

^{1}H NMR of compound A33

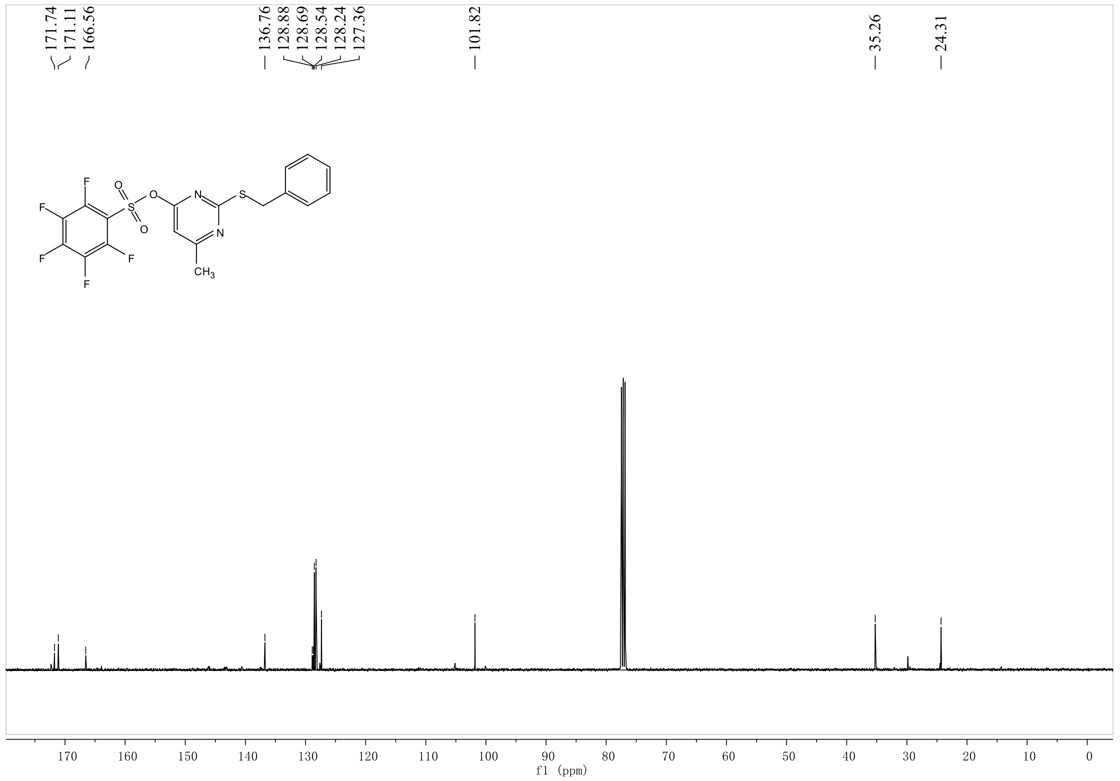

^{13}C NMR of compound A33

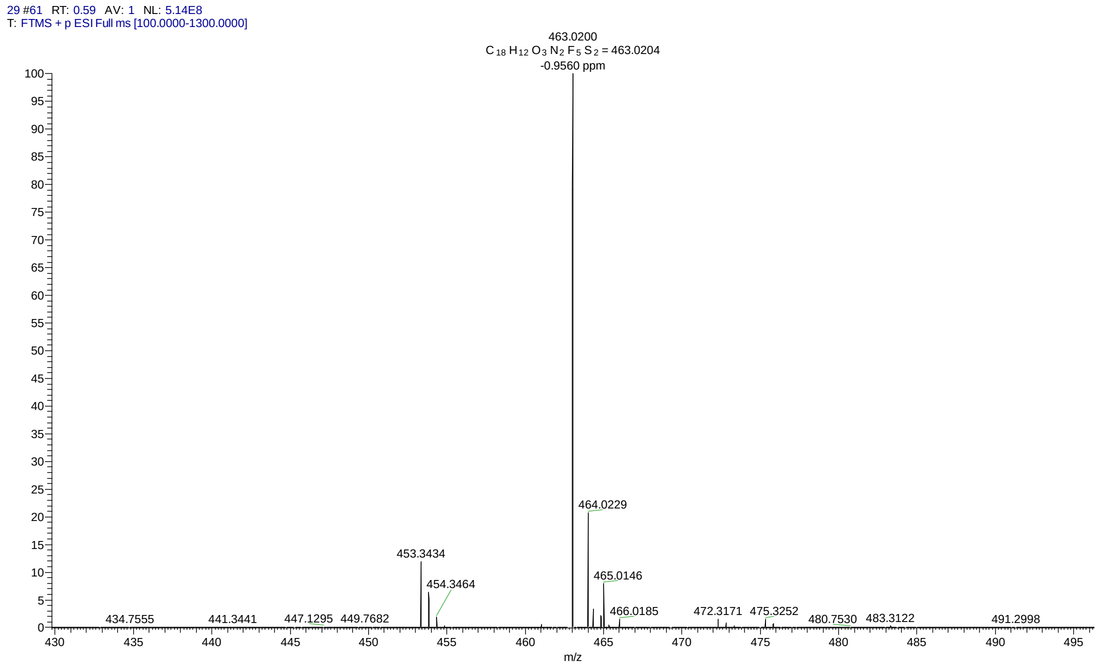

HRMS of compound A33
